# Ultrasound-assisted-one-pot synthesis and antiplasmodium evaluation of 3-substituted-isoindolin-1-ones†

**DOI:** 10.1039/d3ra02829a

**Published:** 2023-09-01

**Authors:** Muhammad Idham Darussalam Mardjan, Muhamad Fadhly Hariadi, Chessy Rima Mustika, Hamzah Shiddiq Saifurofi', Eko Sri Kunarti, Bambang Purwono, Laurent Commeiras

**Affiliations:** a Department of Chemistry, Faculty of Mathematics and Natural Sciences, Universitas Gadjah Mada Yogyakarta 55281 Indonesia idham.darussalam@ugm.ac.id; b Aix Marseille Univ, CNRS, Centrale Marseille, iSm2 Marseille France laurent.commeiras@univ-amu.fr

## Abstract

As the attempts to control malaria through chemotherapy strategies are restricted, we have prepared a small library of 3-substituted-isoindolinones from (*Z*)-3-benzylideneisobenzofuran-1(3*H*)-ones in one-pot fashion under ultrasound irradiation. The one-pot reaction was scalable and efficiently produced the desired products (1a–m) in high yields in a short reaction time. Evaluation of their *in vitro* antiplasmodium assay against the 3D7 (chloroquine-sensitive) and FCR3 (chloroquine-resistant) strains of *Plasmodium falciparum* demonstrated that they displayed moderate to strong antiplasmodium activities (the IC_50_ values ranging from 4.21–34.80 μM) and low resistance indices. The *in silico* prediction of ADME and physicochemical properties showed that the synthesized compounds met the drug-likeliness requirements and featured low toxicity effects. Based on the evaluation of the antiplasmodium profiles, 3-substituted-isoindolinone derivatives of 1a, 1d, 1h, and 1l may become potential antiplasmodium candidates.

## Abstract 2

A library of 3-substituted-isoindolin-1-ones has been synthesized from 3-alkylidenephtalides and primary amines through one-pot reaction under ultrasonic irradiation. Four isoindolin-1-ones have great potential to be antiplasmodium candidates.

## Graphical abstract

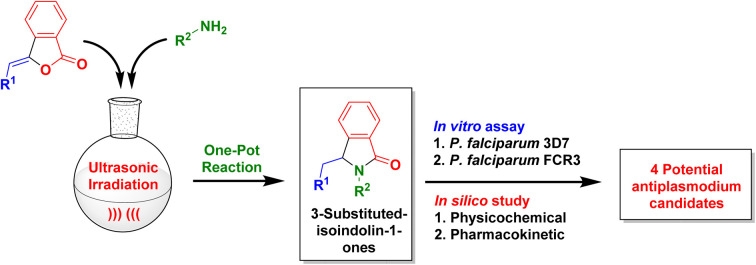

## Introduction

Malaria is one of severe infectious diseases which threaten the global population both in tropical and sub-tropical countries. This disease is transmitted by the female *Anopheles* mosquito where the protozoan parasite of *Plasmodium falciparum* is the most predominant species which is responsible for the mortality.^1^ The efforts to control malaria through chemotherapy are restricted since *Plasmodium falciparum* has developed resistance to artemisinin combination therapies (ACT) and other antimalaria drugs such as chloroquine, quinine, mefloquine and halofantrine.^2,3^ Therefore, the development of more effective antimalaria agents with good resistance profile is urgently required.

**Fig. 1:**
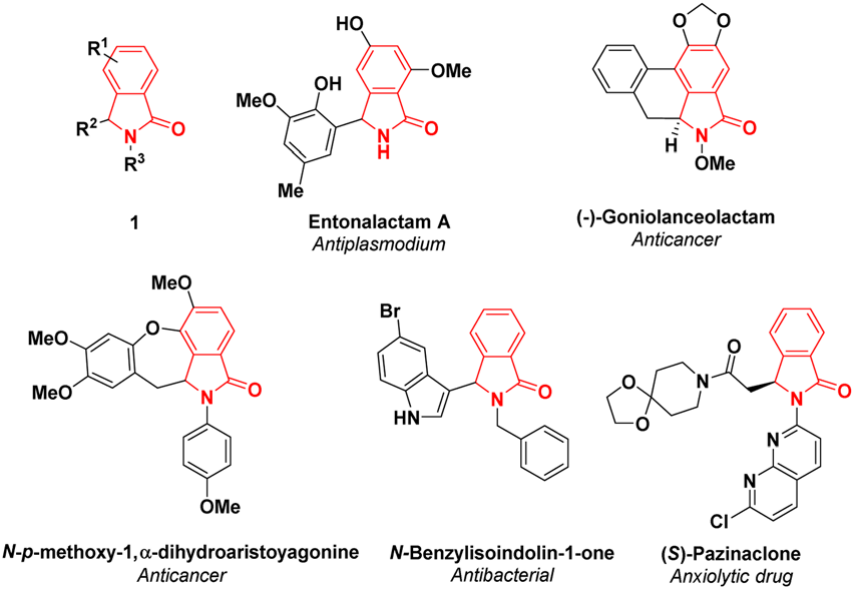
3-Substituted isoindolin-1-ones and their pharmaceutical applications.

Isoindolin-1-ones, particularly 3-substituted-isoindolin-1-ones 1, are one of the most privileged scaffolds found in natural products,^4^ such as entonalactam A^5^ and (−)-goniolanceolactam.^6^ The synthetic γ-lactams have been reported to exhibit various pharmaceutical applications including *N-p*-methoxy-1,α-dihydroaristoyagonine^7^ as anticancer and *N*-benzylisoindolin-1-one^8^ as antibacterial. Moreover, isoindolin-1-one derivatives as exemplified by pazinaclone and pagoclone are commercial anxiolytic drugs.^9^ Due to their interesting applications in medicinal chemistry, a considerable attention has been devoted to produce 3-substituted-isoindolin-1-ones through simple and facile synthetic strategies (Fig. 1).

Isoindolin-1-ones 1 have been synthesized through intramolecular hydroamidation of *ortho*-vinylbenzamides.^10^ The construction of isoindolinone moiety can be carried out through the treatment of isobenzofuran-1(3*H*)-ones with primary amines.^11,12^ Other groups have carried out the *ortho*-alkenylation of substituted benzamides to generate *ortho*-vinybenzamides in the presence of transition metal catalysts such as Co,^13,14^ Ru^15^ and Rh.^16^ Then, the *in situ* generated synthetic intermediates may undergo subsequent intramolecular amidation to furnish lactam 1. Another application of transition metal catalyst in the synthesis of isoindolin-1-ones is Rh-catalyzed-three component reaction using benzamides, ketones and hydrazines.^17^ The formation of isoindolin-1-ones 1 has been achieved through the addition of Grignard reagent to 2-(*N*-sulfinylimines)benzoate esters followed by the intramolecular lactamization.^18–20^ Other strategies involved the modification of other isoindolin-1-one moieties such as alkylation of isoindolin-1-ones at C3 position,^21,22^ hydrogenation of 3-alkylideneisoindolin-1-ones,^23,24^ or reduction of 3-hydroxyisoindolin-1-ones.^25,26^ In spite of these robust methodologies, a facile, rapid and straightforward approach to efficiently generate biologically active isoindolin-1-ones 1 should be still developed.

Our recent study reported an efficient synthesis of 3-hydroxyisoindolin-1-ones 7 through nucleophilic addition reaction of primary amines 3 into (*Z*)-3-benzylideneisobenzofuran-1(3*H*)-ones 2. We demonstrated that the reactivity of 3-hydroxyisoindolin-1-ones 7 can be further extended to give various motifs of isoindolin-1-ones. On reaction of 3-benzyl-3-hydroxyisoindolin-1-one with hydride source (NaBH_3_CN) under acidic condition, 3-benzylisoindolin-1-one was generated through the formation of *N*-acyliminium intermediate (NAI). We also found that ultrasound irradiation may accelerate the rate of those reactions.^27^

Our strategy was driven to meet the green chemistry principles as an efficient approach for 3-isoindolin-1-ones 1. Based on the promising preliminary results, we envisioned that performing both reactions based on ultrasound-assisted-one-pot reaction would efficiently generate 3-isoindolin-1-ones 1. One-pot reaction is well known as an efficient and straightforward strategy in chemical synthesis and has been widely applied in synthesis of drugs.^28^ Application of ultrasound in chemical process has been well documented for the synthesis of various heterocyclic scaffolds. Performing organic reaction under ultrasound irradiation has been found to be more superior than conventional heating or stirring. This green protocol is featured with higher reaction yields, shorter reaction time, energy efficiency and mild conditions.^29–33^

We present, herein, one-pot synthesis of 3-substituted-isoindolin-1-ones 1 under ultrasound irradiation, evaluation of their cytotoxicity against *Plasmodium falciparum* FCR3 (chloroquine-resistant) and 3D7 (chloroquine-sensitive) strains as well as prediction of their physicochemical and pharmacokinetic properties.

## Results and discussion

### Chemistry

The one-pot synthesis of 3-substituted-isoindolin-1-ones 1 was initially investigated by performing the nucleophilic addition reaction between (*Z*)-3-benzylideneisobenzofuran-1(3*H*)-one 2a (prepared from 2-iodobenzoic acid and terminal alkynes *via* ultrasound-assisted-Sonogashira coupling^27^) and butylamine 3a in iso-propanol at 50 °C under ultrasound irradiation. This reaction *in situ* produced the synthetic intermediate of 3-hydroxyisoindolin-1-ones 7. After completion of the reaction, *p*-TSA as the acid catalyst (10 equiv.) and NaBH_3_CN (3 equiv.) as the hydride source were introduced to the subsequent NAI reaction in the same flask without isolating the intermediates 7.

The use of high amount of *p*-TSA was required to completely convert 3-hydroxyisoindolin-1-ones 7 into the corresponding NAI intermediates 8. The reaction was further irradiated at 50 °C for more 1 h (Table 1, entry 1). To our delight, we observed the formation of the desired 3-benzyl-2-butylisoindolin-1-one 1a as a racemic mixture in 63% yield. Attempt to increase the number of hydride source may improve the reaction yields (entries 2–4). The product 1a was furnished in 86% yield when using 10 equiv. of NaBH_3_CN (entry 3). Further survey of solvents (entries 5–7) and catalysts (entries 8–10) demonstrated that the excellent yield of 1a was obtained when the reaction proceeded in acetonitrile in the presence of trifluoroacetic acid (entry 9). The control experiment demonstrated that the lactam 1a was obtained in lower yields when the one-pot reaction was carried out using conventional heating for 10 h (entries 9 *vs.* 11). The increase of both reaction yields and rates of the ultrasound-assisted-one-pot reaction was because the reaction medium received additional energy which was supplied by the mechanical effect of cavitation phenomenon generated from ultrasound waves.^34–36^

**Table 1:** Optimization of ultrasound-assisted-one-pot synthesis of 3-substituted-isoindolin-1-ones^a^

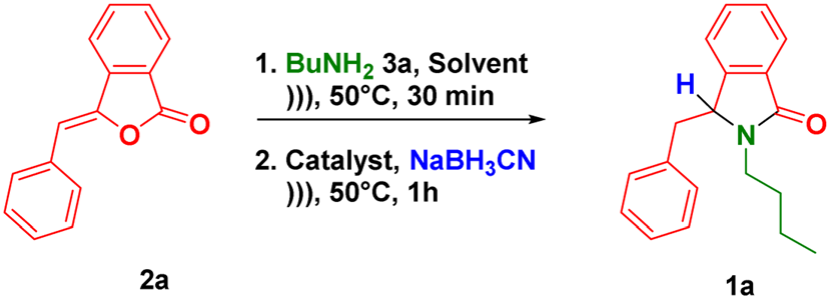				
Entry	[NaBH_3_CN] (equiv.)	Solvent	Catalyst	Yield^b^ (%)
1	3	i-PrOH	*p*-TSA	63
2	5	i-PrOH	*p*-TSA	70
3	10	i-PrOH	*p*-TSA	86
4	15	i-PrOH	*p*-TSA	75
5	10	DMSO	*p*-TSA	79
6	10	MeCN	*p*-TSA	90
7	10	THF	*p*-TSA	77
8	10	MeCN	HCl	72
9	10	MeCN	TFA	93
10	10	MeCN	BF_3_·OEt_2_	80
11^c^	10	MeCN	TFA	77

a The experiments were performed using 0.5 mmol of 2a and 1.0 mmol of 3a under ultrasonic irradiation (40 kHz, 350 W) for 30 min (1st step) + 60 min (2nd step).

b Isolated yields.

c The reaction was performed at 50 °C for 5 h + 5 h using conventional heating.

**Scheme 1:**
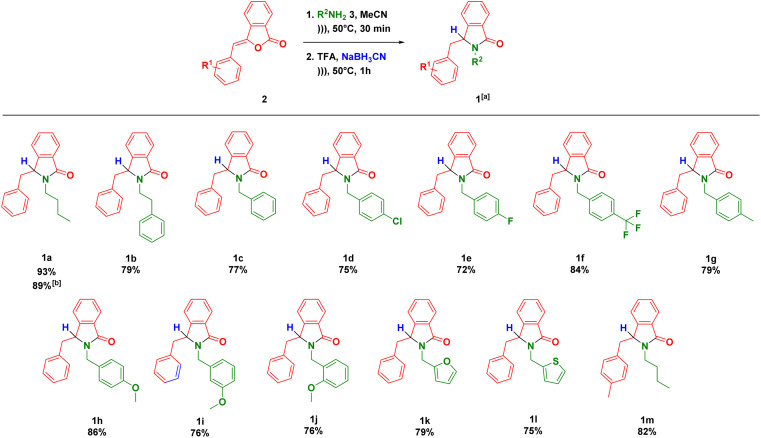
Scopes of reaction, ^*a*^reaction conditions: 2 (0.5 mmol), 3 (1 mmol, 2 equiv.), MeCN (1 mL), under ultrasonic irradiation (40 kHz, 350 W) at 50 °C for 30 min (1st step) + 60 min (2nd step); ^*b*^the reaction was conducted using 10 mmol of 2.

The scopes of one-pot synthesis of 3-substituted-isoindolin-1-ones 1 have been examined under the optimized reaction conditions (Scheme 1). The reaction with phenethylamine afforded the desired product 1b in 79% yield. It should be noted that we did not observe the side product coming from the intramolecular Friedel–Crafts reaction towards the NAI intermediate 8 as previously reported.^37^ The ultrasound-assisted-one-pot reaction also worked well (1c) when benzylamine was used as substrate.

The nature of substituent at benzylamine was then evaluated under the optimal reaction conditions. One of strategies to enhance antiplasmodium activity is by incorporating halogen, trifluoromethyl and methoxy groups to the lead compounds.^38,39^ Therefore, we decided to install both electron withdrawing (Cl, F and CF_3_) and donating (Me and OMe) groups at *para* position of benzylamines. These groups were tolerated to the reaction conditions, delivering the products (1d–h) in high yields. Furthermore, benzylamines with methoxy group at the *ortho* and *meta* positions smoothly reacted to give the corresponding isoindolin-1-ones 1i–j. Heterocyclic amines such as furfurylamine and 2-thiophenemethylamine could be subjected to our methodology leading to the direct formation of the products 1k–l in good yields. As exemplified by 1m, we also managed to employ the precursor of (*Z*)-3-(4-methylbenzylidene)isobenzofuran-1(3*H*)-one in the developed one-pot reaction.

Encouraged by the scopes of the reaction, we then evaluated the scalability of the one-pot reaction. Performing the gram scale-reaction using 10 mmol of (*Z*)-3-benzylideneisobenzofuran-1(3*H*)-one 2a allowed us to obtain the product 1a in 89% yield (Scheme 1).

**Scheme 2:**
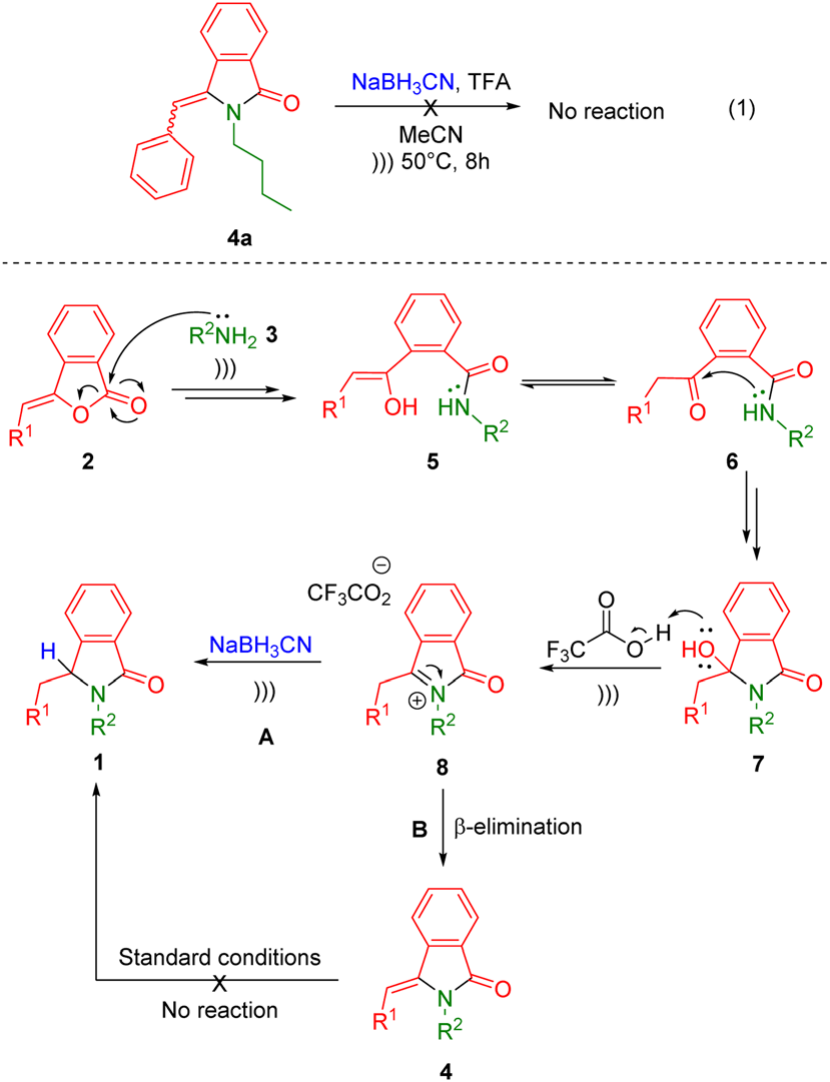
Proposed reaction mechanism.

The plausible reaction mechanism is depicted in Scheme 2. Initial nucleophilic addition of primary amines 3 into (*Z*)-3-benzylideneisobenzofuran-1(3*H*)-ones 2 results in enols 5, which subsequently transformed into the stable keto form 6. Then, the intramolecular nucleophilic addition of ketoamides 6 produces 3-hydroxyisoindolin-1-ones 7. The *in situ* generated lactams 7 reacts with acid catalyst of trifluoroacetic acid leading to the formation the reactive *N*-acyliminium ions (NAI) 8.^40–42^ Then, two possible reaction mechanism can be considered. The first one is the trapping of the reactive ketimines 8 with hydride source (NaBH_3_CN) to directly furnish the isoindolin-1-ones 1 (Scheme 2, route A). In the second pathway, the NAI intermediates 8 may undergo subsequent β-elimination and hydrogenation reactions (Scheme 2, route B). To examine the mechanistic pathway, a control experiment was conducted by introducing 3-benzylideneisoindolin-1-one 4a to the second reaction under the standard condition (Scheme 2, eqn (1)). However, there was no lactam 1 generated even after 8 h of ultrasound irradiation, which let us omit the second scenario. Therefore, the ultrasound-assisted-one-pot reaction of isoindolin-1-ones 1 may proceed through nucleophilic addition or trapping of the NAI intermediates 8 with hydride sources.

### *In vitro* antiplasmodium assay

Having prepared a small library of racemic 3-substituted-isoindolin-1-ones, we then turned our attention to the evaluation of their antiplasmodium activities. These products 1a–m were tested *in vitro* against chloroquine-sensitive (3D7) and -resistant (FCR3) strains of *Plasmodium falciparum*.^43^ The antiplasmodium activities were presented as mean of IC_50_ values.

The results of the antiplasmodium assay are presented in Table 2. We were pleased to observe that the *N*-butylisoindolin-1-one 1a displayed great activities of 8.18 and 5.98 μM against chloroquine-sensitive (3D7) and -resistant (FCR3) strains, respectively. The antiplasmodium activity was reduced when phenylethyl group was installed in the isoindolin-1-one skeleton 1b. Introducing halogen substituents, such as Cl, F and CF_3_, on the *N*-benzyl moiety at 4-position gave positive influence on antiplasmodium activity if compared with hydrogen substituent (1c*versus*1d–f). However, a slight reduction of activity against 3D7 strain was detected for 1d derivative. The presence of halogen substituents on the bioactive compounds may improve their pharmacological properties (for example lipophilicity, metabolic stability, and membrane permeation) and may block metabolic pathway such as cytochrome P450 oxidation.^44,45^ The effect of electron donating groups, such as Me and OMe, was evaluated and enhanced activities against both strains was observed (1c*versus*1g–h). Change in the position of methoxy groups to 3- and 2- positions reduced their antiplasmodium activity, signifying the importance of substituent at 4-position (1g*versus*1h–i). The position of substituent may direct the orientation of molecules and affect the binding ability and energy towards the target proteins of *Plasmodium* parasites.^46^ Interestingly, heterocycle-substituted-isoindolin-1-ones 1k–l exhibited high activities against both strains. However, the decreased activities on 3D7 and FCR3 strains were observed when methyl group was installed at *C*-benzyl moiety (1a*versus*1m).

**Table 2:** *In vitro* data and calculated physical properties of isoindolin-1-ones 1

Compound	IC_50_ (μM)		Resistance indices	Molecular weight (g mol^−1^)	Log *P*	HBD	HBA
	3D7	FCR3					
1a	8.18	5.98	0.73	279.38	3.81	0	1
1b	32.60	26.90	0.83	327.42	4.34	0	1
1c	15.90	27.53	1.73	313.39	4.03	0	1
1d	15.89	7.48	0.47	347.84	4.59	0	1
1e	8.73	10.45	1.20	331.38	4.36	0	2
1f	13.85	4.21	0.30	381.39	5.07	0	4
1g	8.25	6.77	0.82	327.42	4.40	0	1
1h	12.28	14.38	1.17	343.42	4.03	0	2
1i	27.58	28.23	1.02	343.42	4.07	0	2
1j	34.80	30.37	0.87	343.42	4.01	0	2
1k	13.08	5.22	0.40	303.35	3.35	0	2
1l	9.50	6.19	0.65	319.42	4.04	0	1
1m	33.96	9.31	0.27	293.40	4.17	0	1
CQ	0.01	0.11	11	319.87	4.15	1	2

The antiplasmodium activity can be classified into 5 categories, including very active (IC_50_ < 1 μM), active (IC_50_ 1–20 μM), moderate (IC_50_ 20–100 μM), low (IC_50_ 100–200 μM), and not active (IC_50_ > 200 μM). By considering the IC_50_ values (Table 2), most of the isoindolin-1-one derivatives can be classified as active antiplasmodium agents against both parasite strains. Some of tested compounds showed moderate activities (for instance 1b, 1i, 1j, 1m against 3D7 strain and 1b, 1c, 1i, 1j against FCR3 strain).

For all tested compounds, the corresponding resistance indice (RI) was also calculated (ratio IC_50_(FCR3)/IC_50_(3D7)). The high value of RI correlates to the high level of resistance, while low RI value indicates that the compounds have potential as antiplasmodium agents.^47,48^ It should be noted that the RI values of all products were relatively low and in the range of 0.27–1.73. Despite the antiplasmodium activity of chloroquine against both strains were higher than the tested compounds, the RI values of the isoindolin-1-ones 1a–m were 6–40 times lower than that of chloroquine.

### Physicochemical and pharmacokinetic properties

The physicochemical properties were calculated through http://www.swissadme.ch/ to predict the drug likeliness of the products based on Lipinski “rule of five”.^49,50^ The calculation of the tested compounds (Table 1) showed that (a) the molecular weight was lower than 500; (b) log *P* value was lower than 5; (c) number of hydrogen bond donor (HBD) was lower than 5 and (d) number of hydrogen acceptor was lower than 10. All the tested compounds were compliant to the Lipinski rule, indicating that isoindolin-1-ones 1a–m may be easily absorbed, have good permeability and promising potential as antiplasmodium agents.

The *in silico* pharmacokinetic properties of synthesized compounds were also predicted using the online web tools (Tables 3 and 4). The screening of pharmacokinetic properties like absorption, distribution, metabolism, and toxicity parameters were evaluated using the PreADMET web (http:/preadmet.bmdrc.org), while the excretion parameters were calculated using the pkCSM web (https://biosig.lab.uq.edu.au/pkcsm/). The absorption of drugs depends on the human intestinal absorption (HIA), CaCo-2 cell permeability and Madin–Darby canine kidney (MDCK) cell permeability. The results showed that all lactams 1a–m were predicted to have more than 70% HIA values, suggesting that they had good human intestinal absorption. For CaCo-2 cell permeability, all isoindolin-1-ones 1a–m exhibited moderate permeability. On the MDCK cell permeability, five derivatives (1a, 1b, 1c, 1i and 1k) were anticipated to show moderate permeability and the other compounds were expected to display low permeability.

**Table 3:** Predicted absorption and distribution paramaters of isoindolin-1-ones 1

Compound	Absorption			Distribution	
	HIA (%)	Caco-2 cell permeability (nm s^−1^)	MDCK (nm s^−1^)	PPB (%)	Blood–brain barrier penetration (*C*^brain^/*C*^blood^)
1a	100	52.05	34.49	97.18	1.31
1b	100	52.23	47.21	94.75	1.89
1c	100	51.04	32.47	94.85	2.20
1d	100	54.23	3.23	100	1.05
1e	100	54.27	2.97	99.80	0.64
1f	100	41.86	0.04	99.08	4.65
1g	100	29.52	23.99	85.81	10.36
1h	100	53.31	3.49	93.15	0.21
1i	100	53.31	53.31	93.10	0.21
1j	100	53.25	13.25	92.98	0.21
1k	99.24	56.11	223.93	94.82	3.18
1l	97.48	53.23	23.77	99.51	2.29
1m	100	52.15	16.49	97.21	2.21
Required parameters	>20	>4	>25	>90	>0.1

**Table 4:** Predicted metabolism and excretion paramaters of isoindolin-1-ones 1

Compound	Metabolism						Excretion
	CYP2C19 inhibition	CYP2C9 inhibition	CYP2D6 inhibition	CYP2D6 substrate	CYP3A4 inhibition	CYP3A4 substrate	Total clearance (log ml min^−1^ kg^−1^)
1a	None	None	Inhibitor	None	None	Substrate	0.35
1b	None	None	Inhibitor	None	None	Substrate	0.30
1c	None	None	Inhibitor	None	None	Substrate	0.27
1d	None	None	Inhibitor	None	None	Substrate	−0.05
1e	None	None	Inhibitor	None	None	Substrate	1.63
1f	None	None	Inhibitor	None	None	Substrate	0.06
1g	Inhibitor	Inhibitor	Inhibitor	Substrate	None	Substrate	0.27
1h	None	None	None	None	None	Substrate	0.29
1i	None	None	None	None	None	Substrate	0.29
1j	None	None	None	None	None	Substrate	0.28
1k	None	None	None	None	None	Substrate	0.29
1l	None	None	Inhibitor	None	None	Substrate	0.11
1m	None	None	Inhibitor	None	None	Substrate	0.35

The blood–brain barrier (BBB) penetration and plasma protein binding (PPB) are used to characterize the distribution of compounds. All compounds 1a–m exhibited higher BBB penetration values in the range 0.21–10.36, indicating that they can cross the BBB. For the plasma protein binding, values of % bound of all isoindolin-1-ones 1a–m were estimated more than 90%, indicating decreased excretion and increased half-life.^51^

Cytochrome P450 isoforms (CYP2C19, CYP2C9, CYP2D6 and CYP3A4) is an important enzyme for drug metabolism in liver. Metabolism is predicted based on the CYP models for substrate or inhibition. This investigation revealed that most of 3-substituted-isoindolin-1-ones were non-inhibitors for CYP2C19 and CYP2C9 and were not substrates for CYP2D6, except 1g. Only a few compounds (1i–k) were not inhibitor for CYP2D6. The compound 1a–m were non-inhibitors and substrate of the CYP3A4 enzyme.

Excretion is predicted based on the total clearance. The higher the total clearance value of the compound, the faster the excretion process.^52^ The prediction showed that the total clearance of 1e was the highest and followed by 1a, 1m, 1b, 1h, 1i, 1k, 1j, 1c, 1g, 1l, 1f and 1d.

The toxicity of the compounds 1a–m was predicted by the Ames toxicity (mutagenicity) and carcinogenicity mouse and rat. The toxicity prediction showed that all compounds act as mutagen. Only compound 1e and 1f had positive carcinogenicity in mouse while only compound 1k had positive for rat.

## Conclusion

Numbers of substituted 3-substitutedisoindolin-1-ones 1 has been conveniently and directly accessed from various (*Z*)-3-benzylideneisobenzofuran-1(3*H*)-ones 2 and primary amines 3 thanks to ultrasound-assisted-one-pot reaction strategy. The developed methodology provided the desired compounds 1a–m as a racemic mixture in good to excellent yields and can be scaled up using 10 mmol scale. The results showed that introducing aliphatic, halogen, methyl, methoxy and heterocyclic groups on isoindolin-1-one scaffold could enhance their antiplasmodium activities against chloroquine-sensitive (3D7) and -resistant (FCR3) strains of *Plasmodium falciparum*. In addition, the presence of substituent at 4-position of *N*-benzyl moiety played important role in antiplasmodium activity. Based on the *in vitro* antiplasmodium activity, the resistance indice value, the prediction of physicochemical and pharmacokinetic properties, 4 out of 13 tested compounds, namely 1a, 1d, 1h, and 1l, may become promising antiplasmodium agents.

## Materials and methods

### Chemistry

All the experiments were carried out under argon atmosphere in an ultrasonic bath (Powersonic 505, 40 kHz, 350 W). The solvents for the synthesis were initially distilled from calcium hydride. The chemicals used in this study were purchased from Merck and used without any further purification. The reaction was monitored using thin layer chromatography on Merck Kieselgel 60 F254 plates and was observed under UV light (254 nm). The purification of product was carried out using column chromatography on Merck Kiesgel 60 (0.040–0.063 nm).

The melting point was determined using the electrothermal apparatus (Electrothermal 9100). The NMR spectra were recorded on JEOL JNM-ECZ500R (at 500 MHz for ^1^H and 125 MHz for ^13^C). The chemical shifts were reported in parts per million (ppm) relative to the internal solvent signal of CDCl_3_ (*δ*_H_ 7.26 ppm and *δ*_C_ 77.16 pm). Multiplicity is indicated as follows: s (singlet), d (doublet), t (triplet), q (quartet), m (multiplet), ddd (doublet of doublet of doublet), dd (doublet of doublet), dt (doublet of triplet), and td (triplet of doublet). The additional NMR analysis was conducted using DEPT, COSY and HMQC. The high-resolution mass spectrometry experiments were conducted using a QSTAR Elite mass spectrometer (Applied Biosystems SCIEX) or a SYNAPT G2 HDMS mass spectrometer (Waters) equipped with an electrospray ionization source operated in the positive ion mode. The IR spectra were recorded from Shimadzu Prestige-21, where the samples were prepared as KBr pellets.

### General synthesis of 3-alkylisoindolin-1-ones (1)

(*Z*)-3-benzylideneisobenzofuran-1(3*H*)-one derivatives 2 (0.5 mmol, 1 equiv.) and primary amines 3 (1 mmol, 2 equiv.) was dissolved in 1 mL of acetonitrile. The flask was placed in the pre-heated ultrasonic bath (50 °C) and the reaction was carried out for 30 min. The reaction mixture was placed in the ice bath, followed with the addition of NaBH_3_CN (314 mg, 5 mmol, 10 equiv.) and trifluoroacetic acid (0.39 mL, 5 mmol, 10 equiv.). The reaction was continued under ultrasonic irradiation at 50 °C for 60 min. The reaction was quench with the addition of the saturated aqueous solution of NaHCO_3_, followed with the extraction with dichloromethane (3 × 5 mL). The combined organic layer was washed with brine, dried over Na_2_SO_4_, filtered and removed under vacuum. The crude product was purified by column chromatography using the eluent of *n*-hexane/ethyl acetate (9 : 1).

#### 3-Benzyl-2-butylisoindolin-1-one (1a)

Small scale: white solid; yield: 130 mg (93%); mp: 88–91 °C. Scaled up synthesis: white solid; 2.48 g (89%); mp: 89–92 °C. IR (KBr): 3024, 2970, 2924, 2862, 1681, 1604, 1458, 1419, 1273, 1095, 702 cm^−1^; ^1^H-NMR (CDCl_3_, 500 MHz): *δ* = 7.78–7.76 (m, 1H, CH_Ar_), 7.40–7.36 (m, 2H, CH_Ar_), 7.28–7.22 (m, 3H, CH_Ar_), 7.09–7.07 (m, 2H, CH_Ar_), 6.92–6.90 (m, 1H, CH_Ar_), 4.80 (dd, *J* = 8 and 4.5 Hz, 1H, CH), 4.13–4.07 (m, 1H, CH_2_), 3.40 (dd, *J* = 14 and 4.5 Hz, 1H, CH_2_), 3.22 (ddd, *J* = 14, 8.5, 5.5 Hz, 1H, CH_2_), 2.84 (dd, *J* = 14 and 8 Hz, 1H, C*H*_2_), 1.71–1.59 (m, 2H, CH_2_), 1.40–1.32 (m, 2H, CH_2_), 0.96 (t, *J* = 7.5 Hz, 3H, CH_3_); ^13^C-NMR (CDCl_3_, 125 MHz): *δ* = 168.3 (C=O), 144.9 (C_Ar_), 136.1 (C_Ar_), 132.5 (C_Ar_), 130.8 (CH_Ar_), 129.6 (2CH_Ar_), 128.6 (2CH_Ar_), 128.2 (CH_Ar_), 127.1 (CH_Ar_), 123.6 (CH_Ar_), 123.0 (CH_Ar_), 60.0 (CH), 40.0 (CH_2_), 38.5 (CH_2_), 30.5 (CH_2_), 20.2 (CH_2_), 13.9 (CH_3_); HR-MS (ESI): *m*/*z* [M + Na]^+^ cald for C_19_H_21_NONa^+^: 302.1515, found: 302.1517.

#### 3-Benzyl-2-phenethylisoindolin-1-one (1b)

Yellow oil; yield: 130 mg (79%); IR (nujol): 3023, 2970, 2924, 2860, 1681, 1604, 1412, 1080, 756, 702 cm^−1^; ^1^H-NMR (CDCl_3_, 500 MHz): *δ* = 7.79–7.77 (m, 1H, CH_Ar_), 7.41–7.35 (m, 2H, CH_Ar_), 7.30–7.21 (m, 6H, CH_Ar_), 7.19–7.17 (m, 2H, CH_Ar_), 6.98–6.96 (m, 2H, CH_Ar_), 6.86–6.85 (m, 1H, CH_Ar_), 4.52 (dd, *J* = 7.5 and 5.0 Hz, 1H, CH), 4.33–4.27 (m, 1H, CH_2_), 3.44–3.34 (m, 1H, CH_2_), 3.25 (dd, *J* = 14.0 and 5.0 Hz, 1H, CH_2_), 3.01–2.90 3.44–3.34 (m, 2H, CH_2_), 2.76 (dd, *J* = 14.0 and 8.0 Hz, 1H, CH_2_); ^13^C-NMR (CDCl_3_, 125 MHz): *δ* = 168.4 (C=O), 145.1 (C_Ar_), 139.1 (C_Ar_), 136.2 (C_Ar_), 132.3 (C_Ar_), 131.0 (CH_Ar_), 129.5 (2CH_Ar_), 128.9 (2CH_Ar_), 128.8 (2CH_Ar_), 128.6 (2CH_Ar_), 128.2 (CH_Ar_), 127.1 (CH_Ar_), 126.7 (CH_Ar_), 123.6 (CH_Ar_), 122.9 (CH_Ar_), 60.8 (CH), 42.2 (CH_2_), 38.6 (CH_2_), 35.0 (CH_2_); HR-MS (ESI): *m*/*z* [M + H]^+^ cald for C_23_H_22_NO^+^: 328.1696, found: 328.1693.

#### 2,3-Dibenzylisoindolin-1-one (1c)

Light yellow solid; mp: 98–101 °C; yield: 121 mg (77%); IR (KBr): 3024, 2970, 2924, 2860, 1681, 1604, 1435, 1072, 972, 694 cm^−1^; ^1^H-NMR (CDCl_3_, 500 MHz): *δ* = 7.85–7.83 (m, 1H, CH_Ar_), 7.43–7.37 (m, 2H, CH_Ar_), 7.34–7.28 (m, 3H, CH_Ar_), 7.25–7.21 (m, 5H, CH_Ar_), 7.00–6.98 (m, 2H, CH_Ar_), 6.87 (d, *J* = 7.5 Hz, 1H, CH_Ar_), 5.46 (d, *J* = 15.0 Hz, 1H, CH_2_), 4.57 (dd, *J* = 8.0 and 5.0 Hz, 1H, CH), 4.22 (d, *J* = 15.0 Hz, 1H, CH_2_), 3.37 (dd, *J* = 14.0 and 5.5 Hz, 1H, CH_2_), 2.83 (dd, *J* = 14.0 and 8.0 Hz, 1H, CH_2_); ^13^C-NMR (CDCl_3_, 125 MHz): *δ* = 168.6 (C=O), 145.2 (C_Ar_), 137.2 (C_Ar_), 136.2 (C_Ar_), 132.1 (C_Ar_), 131.2 (CH_Ar_), 129.6 (2CH_Ar_), 128.9 (2CH_Ar_), 128.7 (2CH_Ar_), 128.3 (CH_Ar_), 128.2 (2CH_Ar_), 127.8 (CH_Ar_), 127.1 (CH_Ar_), 123.9 (CH_Ar_), 123.1 (CH_Ar_), 59.7 (CH), 44.4 (CH_2_), 38.5 (CH_2_); HR-MS (ESI): *m*/*z* [M + H]^+^ cald for C_22_H_20_NO^+^: 314.1539, found: 314.1539.

#### 3-Benzyl-2-(4-chlorobenzyl)isoindolin-1-one (1d)

Yellow solid; mp: 108–110 °C; yield: 131 mg (75%); IR (KBr): 3020, 2975, 2925, 2860, 1680, 1603, 1404, 1072, 879, 702 cm^−1^; ^1^H-NMR (CDCl_3_, 500 MHz): *δ* = 7.85–7.83 (m, 1H, CH_Ar_), 7.45–7.39 (m, 2H, CH_Ar_), 7.31–7.24 (m, 5H, CH_Ar_), 7.12–7.11 (m, 2H, CH_Ar_), 7.02–7.00 (m, 2H, CH_Ar_), 6.91 (d, *J* = 8.0 Hz, 1H, CH_Ar_), 5.36 (d, *J* = 15.0 Hz, 1H, CH_2_), 4.57 (dd, *J* = 7.5 and 5.0 Hz, 1H, CH), 4.18 (d, *J* = 15.0 Hz, 1H, CH_2_), 3.30 (dd, *J* = 14.0 and 5.5 Hz, 1H, CH_2_), 2.86 (dd, *J* = 14.0 and 7.5 Hz, 1H, C*H*_2_); ^13^C-NMR (CDCl_3_, 125 MHz): *δ* = 168.6 (C=O), 145.2 (C_Ar_), 136.2 (C_Ar_), 135.8 (C_Ar_), 131.9 (C_Ar_), 131.4 (CH_Ar_), 129.7 (C_Ar_), 129.5_9_ (2CH_Ar_), 129.5_6_ (2CH_Ar_), 129.1 (2CH_Ar_), 128.7 (2CH_Ar_), 128.4 (CH_Ar_), 127.2 (CH_Ar_), 124.0 (CH_Ar_), 123.1 (CH_Ar_), 59.8 (CH), 43.8 (CH_2_), 38.9 (CH_2_); HR-MS (ESI): *m*/*z* [M + H]^+^ cald for C_22_H_19_ClNO^+^: 348.1150, found: 348.1146.

#### 3-Benzyl-2-(4-fluorobenzyl)isoindolin-1-one (1e)

Yellow solid; mp: 108–112 °C; yield: 119 mg (72%); IR (KBr): 3024, 2975, 2924, 2865, 1682, 1603, 1404, 1219, 1072, 764, 702 cm^−1^; ^1^H-NMR (CDCl_3_, 500 MHz): *δ* = 7.85–7.83 (m, 1H, CH_Ar_), 7.44–7.39 (m, 2H, CH_Ar_), 7.28–7.23 (m, 3H, CH_Ar_), 7.18–7.15 (m, 2H, CH_Ar_), 7.02–6.98 (m, 4H, CH_Ar_), 6.90 (d, *J* = 8.0 Hz, 1H, CH_Ar_), 5.37 (d, *J* = 15.0 Hz, 1H, CH_2_), 4.56 (dd, *J* = 8.0 and 5.0 Hz, 1H, CH), 4.19 (d, *J* = 15.0 Hz, 1H, CH_2_), 3.32 (dd, *J* = 14.0 and 5.5 Hz, 1H, CH_2_), 2.86 (dd, *J* = 14.0 and 7.5 Hz, 1H, CH_2_); ^13^C-NMR (CDCl_3_, 125 MHz): *δ* = 168.6 (C=O), 162.4 (d, *J* = 245 Hz, C_Ar_), 145.2 (C_Ar_), 136.2 (C_Ar_), 133.0 (d, *J* = 3.0 Hz, C_Ar_), 132.0 (C_Ar_), 131.4 (CH_Ar_), 129.9 (d, *J* = 8.0 Hz, 2CH_Ar_), 129.6 (2CH_Ar_), 128.7 (2CH_Ar_), 128.4 (CH_Ar_), 127.2 (CH_Ar_), 124.0 (CH_Ar_), 123.1 (CH_Ar_), 115.7 (d, *J* = 21.5 Hz, 2CH_Ar_), 59.7 (CH), 43.7 (CH_2_), 38.8 (CH_2_); ^13^F-NMR (CDCl_3_, 470 MHz): *δ* = −114.7; HR-MS (ESI): *m*/*z* [M + H]^+^ cald for C_22_H_19_FNO^+^: 332.1445, found: 332.1443.

#### 3-Benzyl-2-(4-(trifluoromethyl)benzyl)isoindolin-1-one (1f)

Yellow oil; yield: 160 mg (84%); IR (nujol): 3022, 2974, 2924, 2860, 1683, 1404, 1217, 1072, 764, 702 cm^−1^; ^1^H-NMR (CDCl_3_, 500 MHz): *δ* = 7.87–7.85 (m, 1H, CH_Ar_), 7.55 (d, *J* = 8.0 Hz, 2H, CH_Ar_), 7.46–7.42 (m, 2H, CH_Ar_), 7.28–7.25 (m, 5H, CH_Ar_), 7.02–7.00 (m, 2H, CH_Ar_), 6.95–6.96 (m, 1H, CH_Ar_), 5.41 (d, *J* = 15.0 Hz, 1H, CH_2_), 4.59 (dd, *J* = 7.5 and 5.0 Hz, 1H, CH), 4.28 (d, *J* = 15.0 Hz, 1H, CH_2_), 3.28 (dd, *J* = 14.0 and 5.5 Hz, 1H, CH_2_), 2.91 (dd, *J* = 14.0 and 7.5 Hz, 1H, CH_2_); ^13^C-NMR (CDCl_3_, 125 MHz): *δ* = 168.8 (C=O), 145.2 (C_Ar_), 141.5 (C_Ar_), 136.1 (C_Ar_), 131.8 (C_Ar_), 131.6 (CH_Ar_), 130.1 (q, *J* = 32.5 Hz, C_Ar_), 129.5 (2CH_Ar_), 128.8 (2CH_Ar_), 128.5 (CH_Ar_), 128.4 (2CH_Ar_), 127.4 (CH_Ar_), 125.9 (CH_Ar_), 125.8 (CH_Ar_), 124.1 (CH_Ar_), 123.1 (CH_Ar_), 122.1 (q, *J* = 270.0 Hz, CF_3_), 60.0 (CH), 44.1 (CH_2_), 39.0 (CH_2_); ^13^F-NMR (CDCl_3_, 470 MHz): *δ* = −62.5; HR-MS (ESI): *m*/*z* [M + H]^+^ cald for C_23_H_19_F_3_NO^+^: 382.1413, found: 382.1415.

#### 3-Benzyl-2-(4-methylbenzyl)isoindolin-1-one (1g)

White solid; mp: 118–120 °C; yield: 130 mg (79%); IR (KBr): 3025, 2973, 2924, 2865, 1681, 1404, 1072, 702 cm^−1^; ^1^H-NMR (CDCl_3_, 500 MHz): *δ* = 7.81 (d, *J* = 8.0 Hz, 1H, CH_Ar_), 7.41–7.34 (m, 2H, CH_Ar_), 7.24–7.21 (m, 3H, CH_Ar_), 7.14–7.10 (m, 4H, CH_Ar_), 7.00–6.97 (m, 2H, CH_Ar_), 6.83 (d, *J* = 8.0 Hz, 1H, CH_Ar_), 5.42 (d, *J* = 15.0 Hz, 1H, CH_2_), 4.54 (dd, *J* = 8.0 and 5.0 Hz, 1H, CH), 4.16 (d, *J* = 15.0 Hz, 1H, CH_2_), 3.36 (dd, *J* = 14.0 and 5.0 Hz, 1H, CH_2_), 2.80 (dd, *J* = 14.0 and 8.0 Hz, 1H, CH_2_), 2.32 (s, 3H, CH_3_); ^13^C-NMR (CDCl_3_, 125 MHz): *δ* = 168.5 (C=O), 145.2 (C_Ar_), 137.5 (C_Ar_), 136.2 (C_Ar_), 134.3 (C_Ar_), 132.2 (C_Ar_), 131.2 (CH_Ar_), 129.6 (2CH_Ar_), 129.5 (2CH_Ar_), 128.6 (2CH_Ar_), 128.33 (2CH_Ar_), 128.32 (CH_Ar_), 127.1 (CH_Ar_), 123.9 (CH_Ar_), 123.1 (CH_Ar_), 59.6 (CH), 44.1 (CH_2_), 38.5 (CH_2_), 21.3 (CH_3_); HR-MS (ESI): *m*/*z* [M + H]^+^ cald for C_23_H_22_NO^+^: 328.1696, found: 328.1693.

#### 3-Benzyl-2-(4-methoxybenzyl)isoindolin-1-one (1h)

White solid; mp: 92–98 °C; yield: 148 mg (86%); IR (KBr): 3025, 2973, 2924, 2860, 1682, 1512, 1404, 1242, 1180, 1072, 756, 702 cm^−1^; ^1^H-NMR (CDCl_3_, 500 MHz): *δ* = 7.82 (d, *J* = 7.0 Hz, 1H, CH_Ar_), 7.41 (td, *J* = 7.0 and 1 Hz, 1H, CH_Ar_), 7.37 (td, *J* = 7.0 and 1 Hz, 1H, CH_Ar_), 7.25–7.23 (m, 3H, CH_Ar_), 7.16 (d, *J* = 8.0 Hz, 2H, CH_Ar_), 7.01 (d, *J* = 8.0 Hz, 1H, CH_Ar_), 7.00 (d, *J* = 7.0 Hz, 1H, CH_Ar_), 6.86–6.84 (m, 1H, CH_Ar_), 6.84 (d, *J* = 8.0 Hz, 2H, CH_Ar_), 5.41 (d, *J* = 15.0 Hz, 1H, CH_2_), 4.55 (dd, *J* = 8.0 and 5.0 Hz, 1H, CH), 4.15 (d, *J* = 15.0 Hz, 1H, CH_2_), 3.79 (s, 3H, CH_3_) 3.37 (dd, *J* = 13.5 and 5.0 Hz, 1H, CH_2_), 2.82 (dd, *J* = 13.5 and 8.0 Hz, 1H, CH_2_); ^13^C-NMR (CDCl_3_, 125 MHz): *δ* = 168.5 (C=O), 159.2 (C_Ar_), 145.2 (C_Ar_), 136.3 (C_Ar_), 132.3 (C_Ar_), 131.2 (CH_Ar_), 129.6 (4CH_Ar_), 129.3 (C_Ar_), 128.7 (2CH_Ar_), 128.3 (CH_Ar_), 127.1 (CH_Ar_), 123.9 (CH_Ar_), 123.1 (CH_Ar_), 114.3 (2CH_Ar_), 59.6 (CH), 55.4 (CH_3_), 43.7 (CH_2_), 38.5 (CH_2_); HR-MS (ESI): *m*/*z* [M + H]^+^ cald for C_23_H_22_NO_2_^+^: 344.1645, found: 344.1645.

#### 3-Benzyl-2-(3-methoxybenzyl)isoindolin-1-one (1i)

Yellow oil; yield: 131 mg (76%); IR (nujol): 3025, 2973, 2924, 2860, 1682, 1512, 1411, 1257, 1049, 702 cm^−1^; ^1^H-NMR (CDCl_3_, 500 MHz): *δ* = 7.83 (d, *J* = 6.5 Hz, 1H, CH_Ar_), 7.42 (td, *J* = 6.5 and 1 Hz, 1H, CH_Ar_), 7.38 (td, *J* = 6.5 and 1 Hz, 1H, CH_Ar_), 7.25–7.22 (m, 4H, CH_Ar_), 7.00 (dd, *J* = 7.5 and 2 Hz, 2H, CH_Ar_), 6.87 (d, *J* = 6.5 Hz, 1H, CH_Ar_), 6.83 (d, *J* = 2 Hz, 1H, CH_Ar_), 6.81 (d, *J* = 2 Hz, 1H, CH_Ar_), 6.76 (t, *J* = 2 Hz, 1H, CH_Ar_), 5.43 (d, *J* = 15.0 Hz, 1H, CH_2_), 4.60 (dd, *J* = 8.0 and 5.0 Hz, 1H, CH), 4.19 (d, *J* = 15.0 Hz, 1H, CH_2_), 3.77 (s, 3H, CH_3_), 3.37 (dd, *J* = 14.0 and 5.0 Hz, 1H, CH_2_), 2.83 (dd, *J* = 14.0 and 8.0 Hz, 1H, CH_2_); ^13^C-NMR (CDCl_3_, 125 MHz): *δ* = 168.5 (C=O), 160.1 (C_Ar_), 145.2 (C_Ar_), 138.8 (C_Ar_), 136.2 (C_Ar_), 132.1 (C_Ar_), 131.2 (CH_Ar_), 129.9 (CH_Ar_), 129.6 (2CH_Ar_), 128.7 (2CH_Ar_), 128.3 (CH_Ar_), 127.1 (CH_Ar_), 124.0 (CH_Ar_), 123.1 (CH_Ar_), 120.6 (CH_Ar_), 113.8 (CH_Ar_), 113.3 (CH_Ar_), 59.7 (CH), 55.4 (CH_3_), 44.3 (CH_2_), 38.6 (CH_2_); HR-MS (ESI): *m*/*z* [M + H]^+^ cald for C_23_H_22_NO_2_^+^: 344.1645, found: 344.1646.

#### 3-Benzyl-2-(2-methoxybenzyl)isoindolin-1-one (1j)

Yellow oil; yield: 130 mg (76%); IR (nujol): 3032, 2973, 2924, 2860, 1682, 1412, 1103, 756, 702 cm^−1^; ^1^H-NMR (CDCl_3_, 500 MHz): *δ* = 7.77 (d, *J* = 7.0 Hz, 1H, CH_Ar_), 7.37–7.32 (m, 2H, CH_Ar_), 7.28–7.20 (m, 5H, CH_Ar_), 6.99–6.98 (m, 2H, CH_Ar_), 6.91–6.90 (m, 2H, CH_Ar_), 6.78 (d, *J* = 7 Hz, 1H, CH_Ar_), 5.23 (d, *J* = 15.0 Hz, 1H, CH_2_), 4.59 (m, 1H and 1H, CH and CH_2_), 3.87 (s, 3H, CH_3_) 3.56–3.53 (m, 1H, CH_2_), 2.80–2.75 (m, 1H, CH_2_); ^13^C-NMR (CDCl_3_, 125 MHz): *δ* = 168.6 (C=O), 157.5 (C_Ar_), 145.3 (C_Ar_), 136.4 (C_Ar_), 132.4 (C_Ar_), 130.9 (CH_Ar_), 130.5 (CH_Ar_), 129.7 (2CH_Ar_), 129.1 (CH_Ar_), 128.5 (2CH_Ar_), 128.1 (CH_Ar_), 127.0 (CH_Ar_), 125.5 (C_Ar_), 123.7 (CH_Ar_), 123.1 (CH_Ar_), 121.1 (CH_Ar_), 110.6 (CH_Ar_), 60.2 (CH), 55.6 (CH_3_), 38.6 (CH_2_), 38.1 (CH_2_); HR-MS (ESI): *m*/*z* [M + H]^+^ cald for C_23_H_22_NO_2_^+^: 344.1645, found: 344.1642.

#### 3-Benzyl-2-(furan-2-ylmethyl)isoindolin-1-one (1k)

Orange solid; mp: 98–101 °C; yield: 120 mg (79%); IR (KBr): 3032, 2973, 2932, 2860, 1682, 1466, 1420, 1211, 1103, 756, 710 cm^−1^; ^1^H-NMR (CDCl_3_, 500 MHz): *δ* = 7.81–7.79 (m, 1H, CH_Ar_), 7.41–7.35 (m, 3H, CH_Ar_), 7.28–7.22 (m, 3H, CH_Ar_), 7.06–7.04 (m, 2H, CH_Ar_), 6.87–6.85 (m, 1H, CH_Ar_), 6.33 (dd, *J* = 3.5 and 1.0 Hz, 1H, CH_Ar_), 6.27 (d, *J* = 3.5 Hz, 1H, CH_Ar_), 5.33 (d, *J* = 16.0 Hz, 1H, CH_2_), 4.67 (dd, *J* = 8.0 and 5.0 Hz, 1H, CH), 4.30 (d, *J* = 15.0 Hz, 1H, CH_2_), 3.47 (dd, *J* = 14.0 and 5.0 Hz, 1H, CH_2_), 2.82 (dd, *J* = 14.0 and 8.5 Hz, 1H, CH_2_); ^13^C-NMR (CDCl_3_, 125 MHz): *δ* = 168.2 (C=O), 150.6 (C_Ar_), 145.2 (C_Ar_), 142.6 (CH_Ar_), 136.2 (C_Ar_), 132.0 (C_Ar_), 131.3 (CH_Ar_), 129.7 (2CH_Ar_), 128.7 (2CH_Ar_), 128.3 (CH_Ar_), 127.2 (CH_Ar_), 123.9 (CH_Ar_), 123.1 (CH_Ar_), 110.6 (CH_Ar_), 108.8 (CH_Ar_), 60.3 (CH), 38.4 (CH_2_), 37.2 (CH_2_); HR-MS (ESI): *m*/*z* [M + H]^+^ cald for C_20_H_18_NO_2_^+^: 304.1332, found: 304.1330.

#### 3-Benzyl-2-(thiophen-2-ylmethyl)isoindolin-1-one (1l)

White solid; mp: 106–108 °C; yield: 120 mg (75%); IR (KBr): 3032, 2973, 2928, 2860, 1682, 1420, 1080, 694, 570 cm^−1^; ^1^H-NMR (CDCl_3_, 500 MHz): *δ* = 7.82 (d, *J* = 8.5 Hz, 1H, CH_Ar_), 7.43–7.38 (m, 2H, CH_Ar_), 7.29–7.23 (m, 4H, CH_Ar_), 7.07 (d, *J* = 7.0 Hz, 2H, CH_Ar_), 6.97–6.95 (m, 2H, CH_Ar_), 6.89 (dd, *J* = 3.5 Hz, 1H, CH_Ar_), 6.27 (d, *J* = 3.5 Hz, 1H, CH_Ar_), 5.53 (d, *J* = 16.0 Hz, 1H, CH_2_), 4.71 (dd, *J* = 7.5 and 5.5 Hz, 1H, CH), 4.42 (d, *J* = 15.0 Hz, 1H, CH_2_), 3.41 (dd, *J* = 13.5 and 5.0 Hz, 1H, CH_2_), 2.88 (dd, *J* = 13.5 and 8.0 Hz, 1H, CH_2_); ^13^C-NMR (CDCl_3_, 125 MHz): *δ* = 168.3 (C=O), 145.3 (C_Ar_), 139.6 (C_Ar_), 136.2 (C_Ar_), 131.9 (C_Ar_), 131.4 (CH_Ar_), 129.6 (2CH_Ar_), 128.7 (2CH_Ar_), 128.4 (CH_Ar_), 127.2 (CH_Ar_), 127.1 (CH_Ar_), 126.9 (CH_Ar_), 125.8 (CH_Ar_), 124.0 (CH_Ar_), 123.1 (CH_Ar_), 59.6 (CH), 38.9 (CH_2_), 38.7 (CH_2_); HR-MS (ESI): *m*/*z* [M + H]^+^ cald for C_20_H_18_NOS^+^: 320.1104, found: 320.1102.

#### 2-Butyl-3-(4-methylbenzyl)isoindolin-1-one (1m)

White solid; mp: 96–101 °C; yield: 121 mg (82%); IR (KBr): 3031, 2973, 2925, 2865, 1682, 1404, 1072, 702 cm^−1^; ^1^H-NMR (CDCl_3_, 500 MHz): *δ* = 7.77–7.76 (m, 1H, CH_Ar_), 7.39–7.37 (m, 2H, CH_Ar_), 7.05 (d, *J* = 8 Hz, 2H, CH_Ar_), 6.95 (d, *J* = 8 Hz, 2H, CH_Ar_), 6.94–6.93 (m, 1H, CH_Ar_), 4.75 (dd, *J* = 8 and 5.0 Hz, 1H, CH), 4.10–4.04 (m, 1H, CH_2_), 3.34 (dd, *J* = 14 and 4.5 Hz, 1H, CH_2_), 3.20 (ddd, *J* = 14, 8.5, 5.5 Hz, 1H, CH_2_), 2.77 (dd, *J* = 14 and 8 Hz, 1H, CH_2_), 2.31 (s, 3H, CH_3_), 1.69–1.58 (m, 2H, CH_2_), 1.39–1.31 (m, 2H, CH_2_), 0.94 (t, *J* = 7.0 Hz, 3H, CH_3_); ^13^C-NMR (CDCl_3_, 125 MHz): *δ* = 168.4 (C=O), 145.1 (C_Ar_), 136.7 (C_Ar_), 133.0 (C_Ar_), 132.6 (C_Ar_), 130.8 (CH_Ar_), 129.4 (2CH_Ar_), 129.3 (2CH_Ar_), 128.2 (CH_Ar_), 123.6 (CH_Ar_), 123.0 (CH_Ar_), 60.1 (CH), 40.0 (CH_2_), 38.0 (CH_2_), 30.6 (CH_2_), 21.2 (CH_3_), 20.3 (CH_2_), 13.9 (CH_3_); HR-MS (ESI): *m*/*z* [M + H]^+^ cald for C_20_H_24_NO^+^: 294.1852, found: 294.1854.

### *In vitro* antiplasmodium assay

The cytotoxicity of products was evaluated using *Plasmodium falciparum* strains of 3D7 (chloroquine-sensitive) and FCR3 (chloroquine-resistant). The antiplasmodium assay was conducted using candle jar method.

For the *in vitro* assay, each synthesized compound was dissolved in DMSO and was prepared in a series of concentration, *i.e.* 10, 5, 2.5, 1.25 and 0.625 μg mL^−1^ in RPMI medium. A total of 100 μL of each series of concentrations was put into the 96-well microplate with three repetition and then 100 μL *Plasmodium* suspension was added. The culture was incubated at 37 °C for 72 h, a thin blood smear was made and treated with 20% Giemsa dyes. The percentage of parasitemia was determined by calculating the number of the infected erythrocytes for minimum 1000 erythrocytes and then used to calculate the inhibition percentage of *P. falciparum* growth. The antiplasmodium activities were presented as mean of IC_50_ values. The IC_50_ value was calculated by probit analysis using SPSS software.

### Physicochemical and pharmacokinetic properties

The pharmacokinetic properties including absorption, distribution, metabolism, and toxicity parameters were screened through the PreADMET web (http:/preadmet.bmdrc.org). Moreover, the excretion parameters were determined using the pkCSM web (https://biosig.lab.uq.edu.au/pkcsm/).

## Author contributions

Conceptualization, M. I. D. M. and L. C.; synthesis and analysis, M. F. H., H. S. S. and M. I. D. M.; antiplasmodium assay, C. R. M.; supervision, M. I. D. M., E. S. K. and B. P.; writing-draft preparation, M. I. D. M. and L. C.; writing-review and editing, M. I. D. M. and L. C. All authors have read the manuscript.

## Conflicts of interest

There are no conflicts to declare.

## Supplementary Material

RA-013-D3RA02829A-s001
