# Supplementary material for: Ultrasound-assisted-one-pot synthesis and antiplasmodium evaluation of 3-substituted-isoindolin-1-ones
Source: RSC Adv. 2023 Sep 1;13(37):25959–67. doi: 10.1039/d3ra02829a (PMC10472802; doi:10.1039/d3ra02829a)
Supplement: RA-013-D3RA02829A-s001 [file RA-013-D3RA02829A-s001.pdf]

Electronic Supplementary Material (ESI) for RSC Advances.
This journal is © The Royal Society of Chemistry 2023

Ultrasonic-assisted-one-pot Synthesis and Antiplasmodium Evaluation of 3-Alkylisoindolin-1-ones

Muhammad Idham Darussalam Mardjan,*[a] Muhamad Fadhly Hariadi, [a] Chessy Rima Mustika, [a] Hamzah Shiddiq Saifurofi', [a] Eko Sri Kunarti, [a] Bambang Purwono, [a] and Laurent Commeiras [b]

[a]Department of Chemistry, Faculty of Mathematics and Natural Sciences, Universitas Gadjah Mada, Yogyakarta 55281, Indonesia

[b]Aix Marseille Univ, CNRS, Centrale Marseille, iSm2, Marseille, France

e-mail: idham.darussalam@ugm.ac.id, laurent.commeiras@univ-amu.fr

General Method

All the experiments were carried out under argon atmosphere in an ultrasonic bath (Powersonic 505, 40 kHz, 350 W). The solvents for the synthesis were initially distilled from calcium hydride. The chemicals used in this study were purchased from Merck and used without any further purification. The reaction was monitored using thin layer chromatography on Merck Kieselgel 60 F254 plates and was observed under UV light (254 nm). The purification of product was carried out using column chromatography on Merck Kiesgel 60 (0.040-0.063 nm).

The melting point was determined using the electrothermal apparatus (Electrothermal 9100). The NMR spectra were recorded on JEOL JNM-ECZ500R (at 500 MHz for ^{1}H and 125 MHz for ^{13}C). The chemical shifts were reported in parts per million (ppm) relative to the internal solvent signal of $CDCl_3$ (δH 7.26 ppm and δC 77.16 pm). Multiplicity is indicated as follows: s (singlet), d (doublet), t (triplet), q (quartet), m (multiplet), ddd (doublet of doublet of doublet), dd (doublet of coublet), dt (doublet of triplet), and td (triplet of doublet). The additional NMR analysis was conducted using DEPT, COSY and HMQC. The high-resolution mass spectrometry experiments were conducted using a QSTAR Elite mass spectrometer (Applied Biosystems SCIEX) or a SYNAPT G2 HDMS mass spectrometer (Waters) equipped with an electrospray ionization source operated in the positive ion mode. The IR spectra were recorded from Shimadzu Prestige-21, where the samples were prepared as KBr pellets.

The cytotoxicity of products was evaluated using *Plasmodium falciparum* strains of 3D7 (chloroquine-sensitive) and FCR3 (chloroquine-resistant). The antiplasmodium assay was conducted using candle jar method. For the *in vitro* assay, each synthesized compound was dissolved in DMSO and was prepared in a series of concentration, i.e. 10, 5, 2.5, 1.25 and 0.625 µg/mL in RPMI medium. A total of 100 µL of each series of concentrations was put into the 96-well microplate with three repetition and then 100 µL *Plasmodium* suspension was added. The culture was incubated at 37 °C for 72 h, a thin blood smear was made and treated with 20% Giemsa dyes. The percentage of parasitemia was determined by calculating the number of the infected erythrocytes for minimum 1,000 erythrocytes and then used to calculate the inhibition percentage of *P. falciparum* growth. The antiplasmodium activities were presented as mean of IC_{50} values. The IC_{50} value was calculated by probit analysis using SPSS software.

The pharmacokinetic properties including absorption, distribution, metabolism, and toxicity parameters were screened through the PreADMET web (http:/preadmet.bmdrc.org). Moreover, the excretion parameters were determined using the pkCSM web (https://biosig.lab.uq.edu.au/pkcsm/).

General Synthesis of 3-Alkylisoindolin-1-ones (1)

(*Z*)-3-benzylideneisobenzofuran-1(3*H*)-one derivatives **2** (0.5 mmol, 1 equiv.) and primary amines **3** (1 mmol, 2 equiv.) was dissolved in 1 mL of acetonitrile. The flask was placed in the pre-heated ultrasonic bath (50 °C) and the reaction was carried out for 30 min. The reaction mixture was placed in the ice bath, followed with the addition of $NaBH_3CN$ (314 mg, 5 mmol, 10 equiv.) and trifluoroacetic acid (0.39 mL, 5 mmol, 10 equiv.). The reaction was continued under ultrasonic irradiation at 50 °C for 60 min. The reaction was quench with the addition of the saturated aqueous solution of $NaHCO_3$, followed with the extraction with dichloromethane (3x5 mL). The combined organic layer was washed with brine, dried over Na_2SO_4, filtered and removed under vacuum. The crude product was purified by column chromatography using the eluent of *n*-hexane/ethyl acetate (9:1).

Synthesis of 3-Benzyl-2-butylisoindolin-1-one (1a)

Following the procedure for the synthesis of 3-alkylisoindolin-1-ones **1** using (*Z*)-3-benzylideneisobenzofuran-1(3*H*)-one **2a** (111 mg, 0.5 mmol, 1 equiv.) and *n*-butylamine (0.099 mL, 1 mmol, 2 equiv.) with purification using column chromatography (*n*-hexane/ethyl acetate, 9:1), afforded the desired compound **1a.**

For scaled up synthesis of **1a**, the same procedure (small scale) of **1a** was conducted by using 2,22 g of (*Z*)-3-benzylideneisobenzofuran-1(3*H*)-one **2a** (10 mmol, 1 equiv.), 1,98 mL of *n*-butylamine (20 mmol, 2 equiv.), $NaBH_3CN$ (6,28 g, 100 mmol, 10 equiv.) and trifluoroacetic acid (7.6 mL, 100 mmol, 10 equiv.).

Small scale (conventional heating): white solid; yield: 107 mg (77%); mp: 89-92°C.

Small scale (ultrasound): white solid; yield: 130 mg (93%); mp: 88-91 °C.

Scaled up synthesis (ultrasound): white solid; 2.48 g (89%); mp: 89-92 °C.

IR (KBr): 3024, 2970, 2924, 2862, 1681, 1604, 1458, 1419, 1273, 1095, 702 cm^{-1}.

^{1}H-NMR ($CDCl_3$, 500 MHz): δ = 7.78-7.76 (m, 1H, CH_{Ar}), 7.40-7.36 (m, 2H, CH_{Ar}), 7.28-7.22 (m, 3H, CH_{Ar}), 7.09-7.07 (m, 2H, CH_{Ar}), 6.92-6.90 (m, 1H, CH_{Ar}), 4.80 (dd, *J* = 8 and 4.5 Hz, 1H, CH), 4.13-4.07 (m, 1H, CH_2), 3.40 (dd, *J* = 14 and 4.5 Hz, 1H, CH_2), 3.22 (ddd, *J* = 14, 8.5, 5.5 Hz, 1H, CH_2), 2.84 (dd, *J* = 14 and 8 Hz, 1H, CH_2), 1.71-1.59 (m, 2H, CH_2), 1.40-1.32 (m, 2H, CH_2), 0.96 (t, *J* = 7.5 Hz, 3H, CH_3).

^{13}C-NMR ($CDCl_3$, 125 MHz): δ = 168.3 (C=O), 144.9 (C_{Ar}), 136.1 (C_{Ar}), 132.5 (C_{Ar}), 130.8 (CH_{Ar}), 129.6 ($2CH_{Ar}$), 128.6 ($2CH_{Ar}$), 128.2 (CH_{Ar}), 127.1 (CH_{Ar}), 123.6 (CH_{Ar}), 123.0 (CH_{Ar}), 60.0 (CH), 40.0 (CH_2), 38.5 (CH_2), 30.5 (CH_2), 20.2 (CH_2), 13.9 (CH_3).

HR-MS (ESI): m/z $[M+Na]^+$ cald for $C_{19}H_{21}NONa^+$: 302.1515, found: 302.1517.

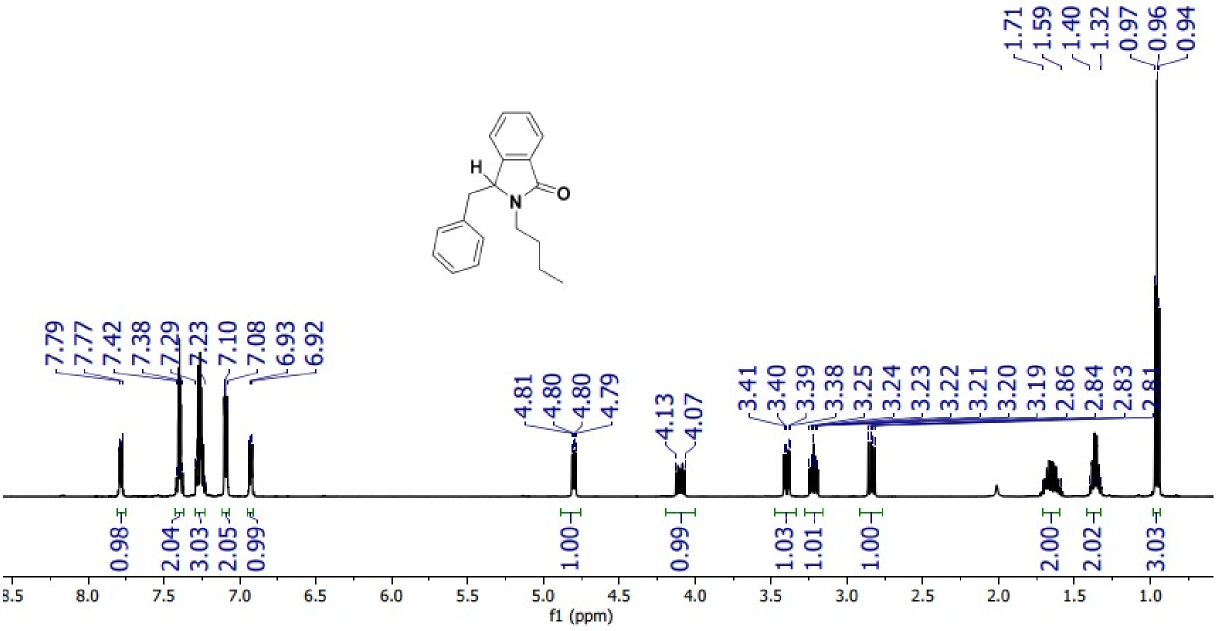

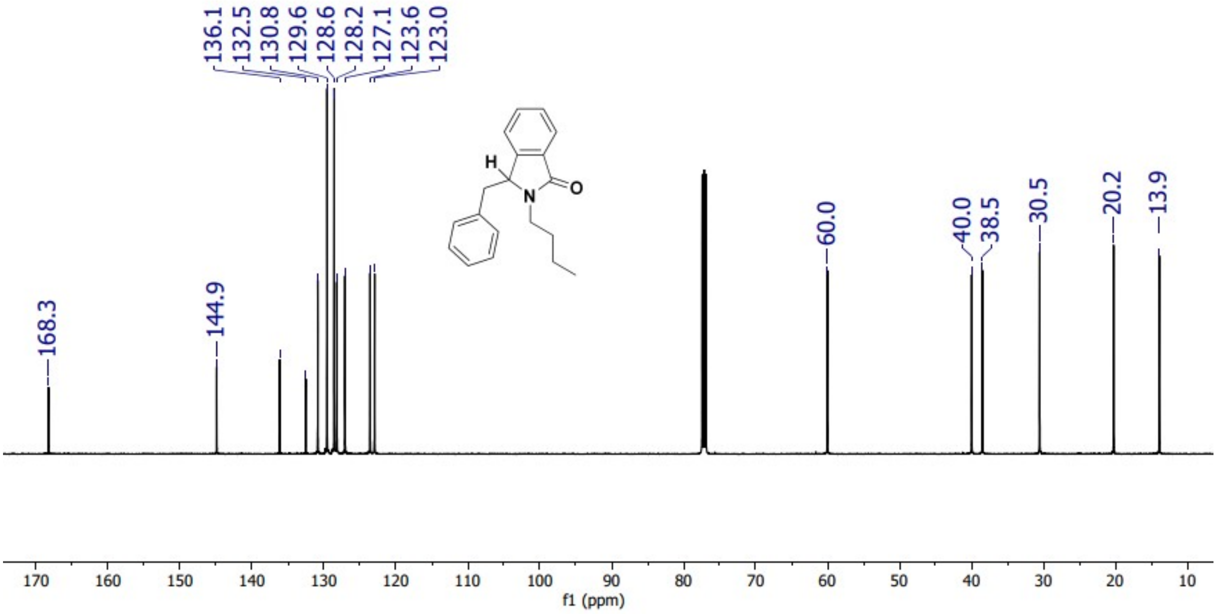
136.1
132.5
130.8
129.6
128.6
128.2
127.1
123.6
123.0
168.3
144.9
H
N
O
60.0
40.0
38.5
30.5
20.2
13.9
170 160 150 140 130 120 110 100 90 80 70 60 50 40 30 20 10
f1 (ppm)

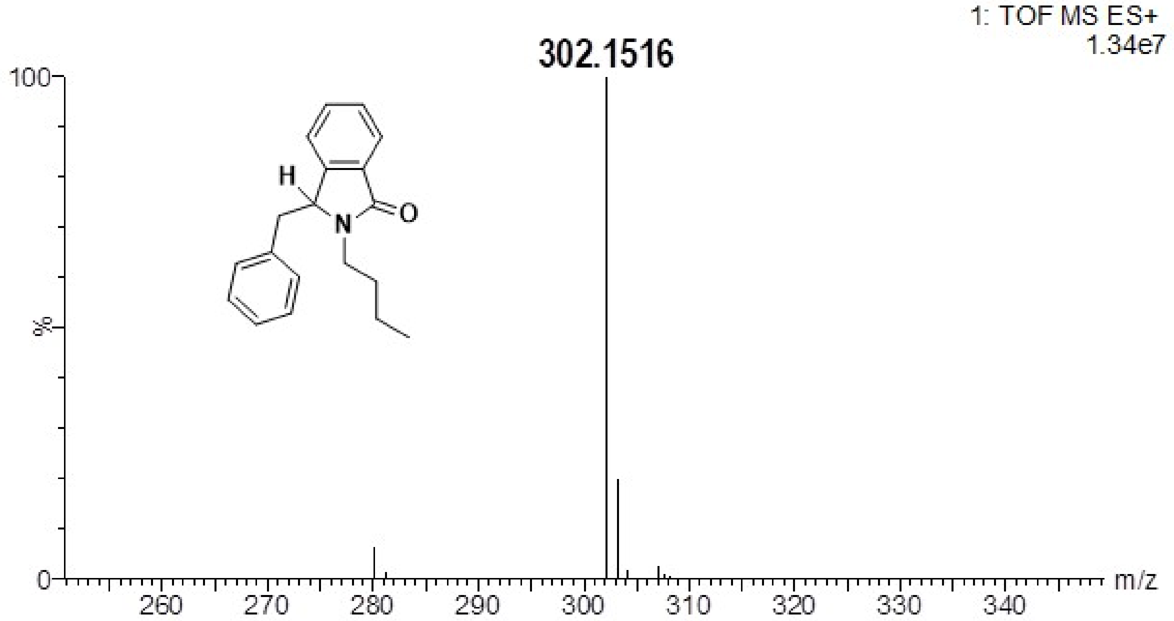
1: TOF MS ES+
1.34e7
302.1516
100
%
0
H
N
O
260 270 280 290 300 310 320 330 340
m/z

Synthesis of 3-Benzyl-2-phenethylisoindolin-1-one (1b)

Following the procedure for the synthesis of 3-alkylisoindolin-1-ones **1** using (*Z*)-3-benzylideneisobenzofuran-1(3*H*)-one **2a** (111 mg, 0.5 mmol, 1 equiv.) and 2-phenethylamine (0.126 mL, 1 mmol, 2 equiv.) with purification using column chromatography (*n*-hexane/ethyl acetate, 9:1), afforded the desired compound **1b.**

Yellow oil; yield: 130 mg (79%).

IR (nujol): 3023, 2970, 2924, 2860, 1681, 1604, 1412, 1080, 756, 702 cm^{-1}.

^{1}H-NMR ($CDCl_3$, 500 MHz): δ = 7.79-7.77 (m, 1H, CH_{Ar}), 7.41-7.35 (m, 2H, CH_{Ar}), 7.30-7.21 (m, 6H, CH_{Ar}), 7.19-7.17 (m, 2H, CH_{Ar}), 6.98-6.96 (m, 2H, CH_{Ar}), 6.86-6.85 (m, 1H, CH_{Ar}), 4.52 (dd, *J* = 7.5 and 5.0 Hz, 1H, CH), 4.33-4.27 (m, 1H, CH_2), 3.44-3.34 (m, 1H, CH_2), 3.25 (dd, *J* = 14.0 and 5.0 Hz, 1H, CH_2), 3.01-2.90 3.44-3.34 (m, 2H, CH_2), 2.76 (dd, *J* = 14.0 and 8.0 Hz, 1H, CH_2)
.

^{13}C-NMR ($CDCl_3$, 125 MHz): δ = 168.4 (C=O), 145.1 (C_{Ar}), 139.1 (C_{Ar}), 136.2 (C_{Ar}), 132.3 (C_{Ar}), 131.0 (CH_{Ar}), 129.5 (2CH_{Ar}), 128.9 (2CH_{Ar}), 128.8 (2CH_{Ar}), 128.6 (2CH_{Ar}), 128.2 (CH_{Ar}), 127.1 (CH_{Ar}), 126.7 (CH_{Ar}), 123.6 (CH_{Ar}), 122.9 (CH_{Ar}), 60.8 (CH), 42.2 (CH_2), 38.6 (CH_2), 35.0 (CH_2).

HR-MS (ESI): m/z $[M+H]^+$ cald for $C_{23}H_{22}NO^+$: 328.1696, found: 328.1693.

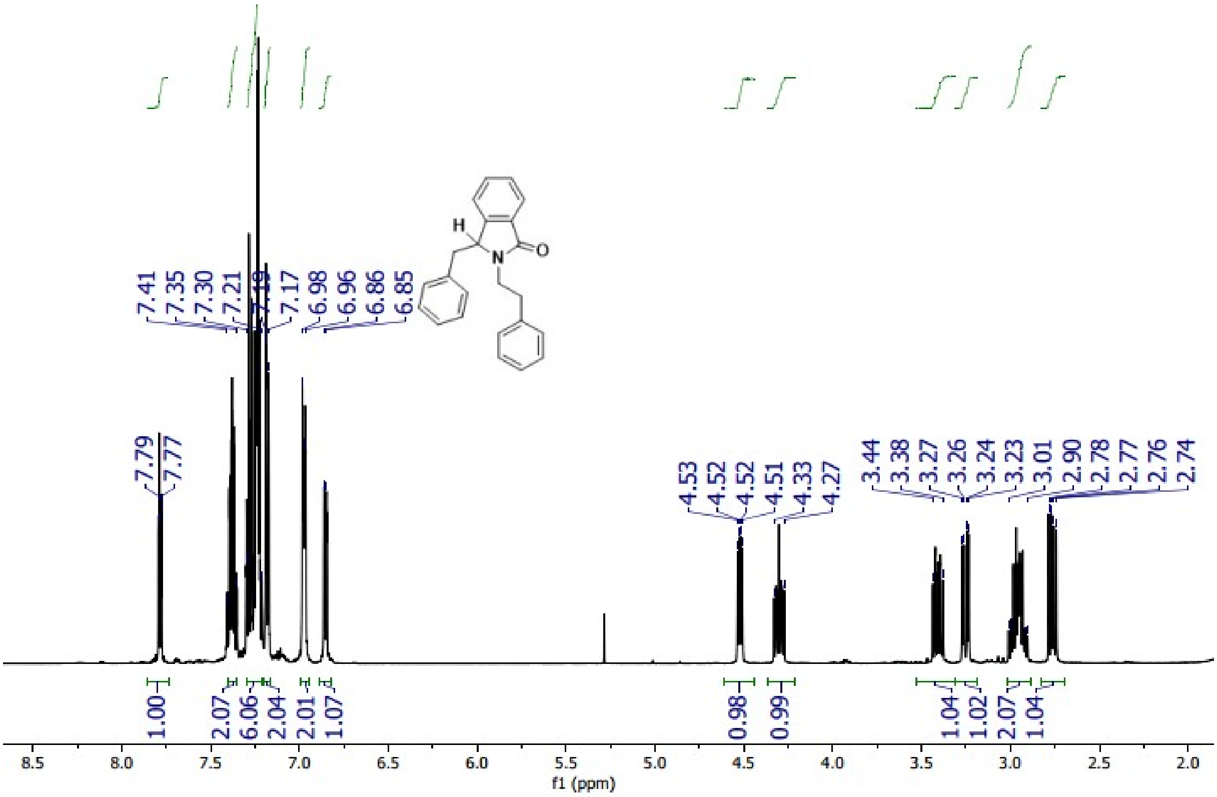

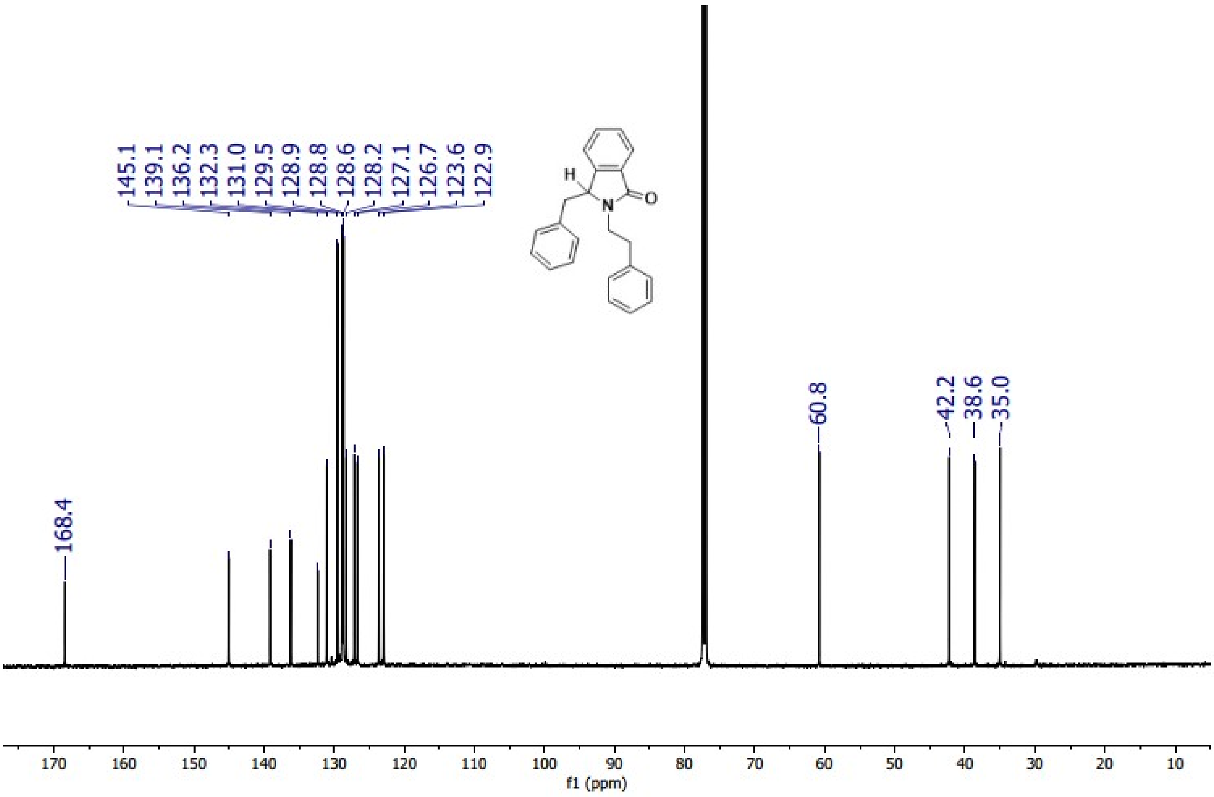
145.1
139.1
136.2
132.3
131.0
129.5
128.9
128.8
128.6
128.2
127.1
126.7
123.6
122.9
168.4
60.8
42.2
38.6
35.0
H
N
O
170
160
150
140
130
120
110
100
90
80
70
60
50
40
30
20
10
f1 (ppm)

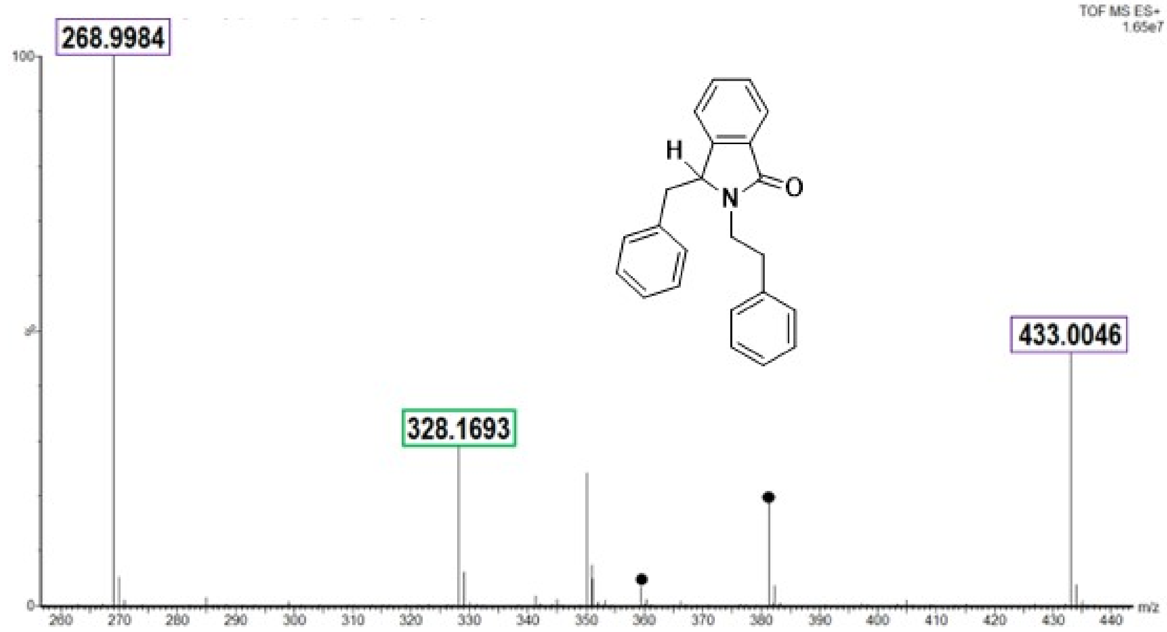
TOF MS ES+
1.65e7
268.9984
328.1693
433.0046
H
N
O
100
%
0
m/z
260
270
280
290
300
310
320
330
340
350
360
370
380
390
400
410
420
430
440

Synthesis of 2,3-Dibenzylisoindolin-1-one (1c)

Following the procedure for the synthesis of 3-alkylisoindolin-1-ones **1** using (*Z*)-3-benzylideneisobenzofuran-1(3*H*)-one **2a** (111 mg, 0.5 mmol, 1 equiv.) and benzylamine (0.11 mL, 1 mmol, 2 equiv.) with purification using column chromatography (*n*-hexane/ethyl acetate, 9:1), afforded the desired compound **1c.**

Light yellow solid; mp: 98-101°C; yield: 121 mg (77%).

IR (KBr): 3024, 2970, 2924, 2860, 1681, 1604, 1435, 1072, 972, 694 cm^{-1}.

^{1}H-NMR ($CDCl_3$, 500 MHz): δ = 7.85-7.83 (m, 1H, CH_{Ar}), 7.43-7.37 (m, 2H, CH_{Ar}), 7.34-7.28 (m, 3H, CH_{Ar}), 7.25-7.21 (m, 5H, CH_{Ar}), 7.00-6.98 (m, 2H, CH_{Ar}), 6.87 (d, *J* = 7.5 Hz, 1H, CH_{Ar}), 5.46 (d, *J* = 15.0 Hz, 1H, CH_2), 4.57 (dd, *J* = 8.0 and 5.0 Hz, 1H, CH), 4.22 (d, *J* = 15.0 Hz, 1H, CH_2), 3.37 (dd, *J* = 14.0 and 5.5 Hz, 1H, CH_2), 2.83 (dd, *J* = 14.0 and 8.0 Hz, 1H, CH_2).

^{13}C-NMR ($CDCl_3$, 125 MHz): δ = 168.6 (C=O), 145.2 (C_{Ar}), 137.2 (C_{Ar}), 136.2 (C_{Ar}), 132.1 (C_{Ar}), 131.2 (CH_{Ar}), 129.6 (2CH_{Ar}), 128.9 (2CH_{Ar}), 128.7 (2CH_{Ar}), 128.3 (CH_{Ar}), 128.2 (2CH_{Ar}), 127.8 (CH_{Ar}), 127.1 (CH_{Ar}), 123.9 (CH_{Ar}), 123.1 (CH_{Ar}), 59.7 (CH), 44.4 (CH_2), 38.5 (CH_2).

HR-MS (ESI): m/z $[M+H]^+$ cald for $C_{22}H_{20}NO^+$: 314.1539, found: 314.1539.

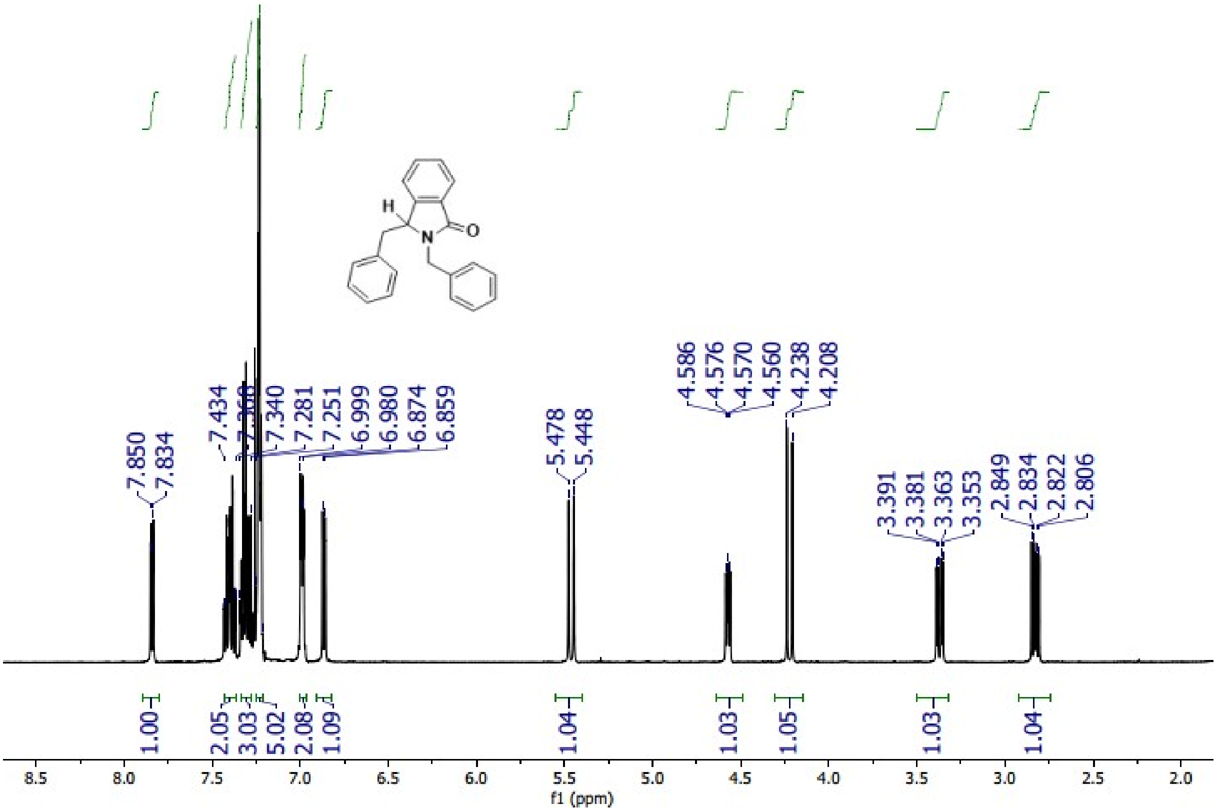

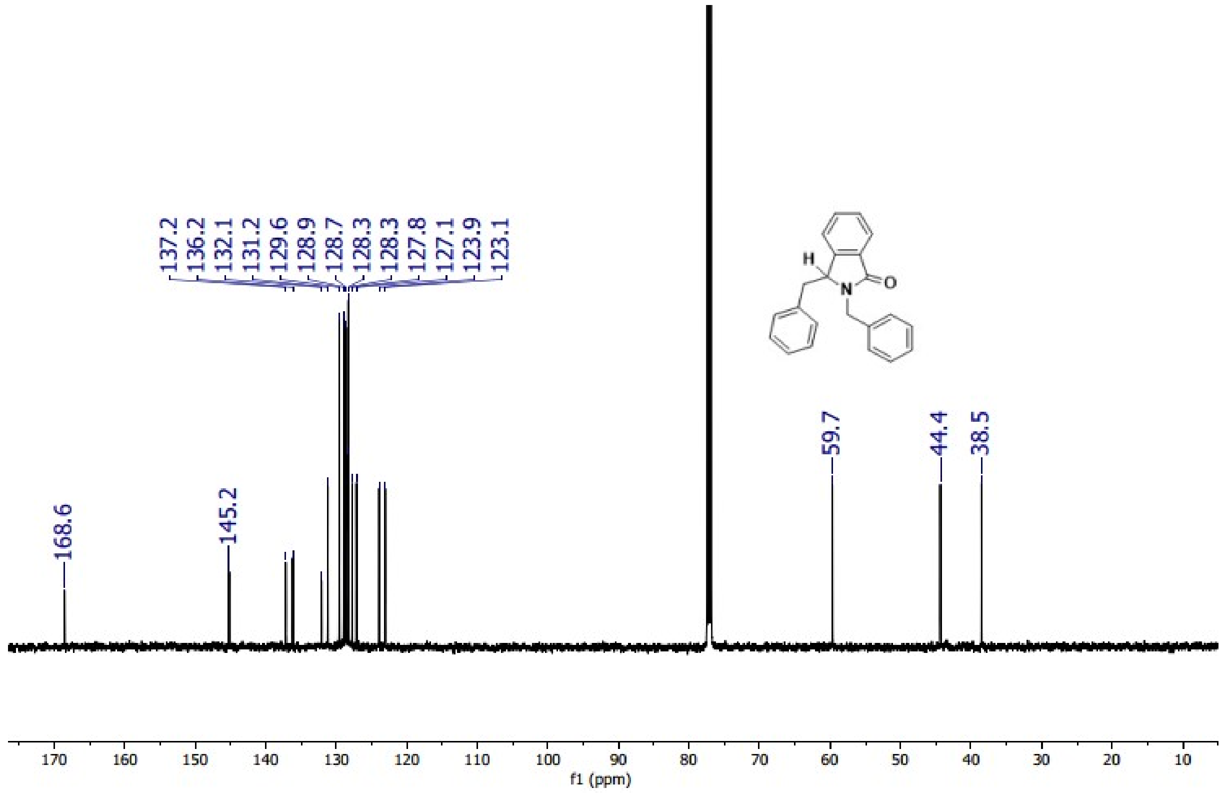

137.2
136.2
132.1
131.2
129.6
128.9
128.7
128.3
128.3
127.8
127.1
123.9
123.1
168.6
145.2
59.7
44.4
38.5
H
N
O
170 160 150 140 130 120 110 100 90 80 70 60 50 40 30 20 10
f1 (ppm)

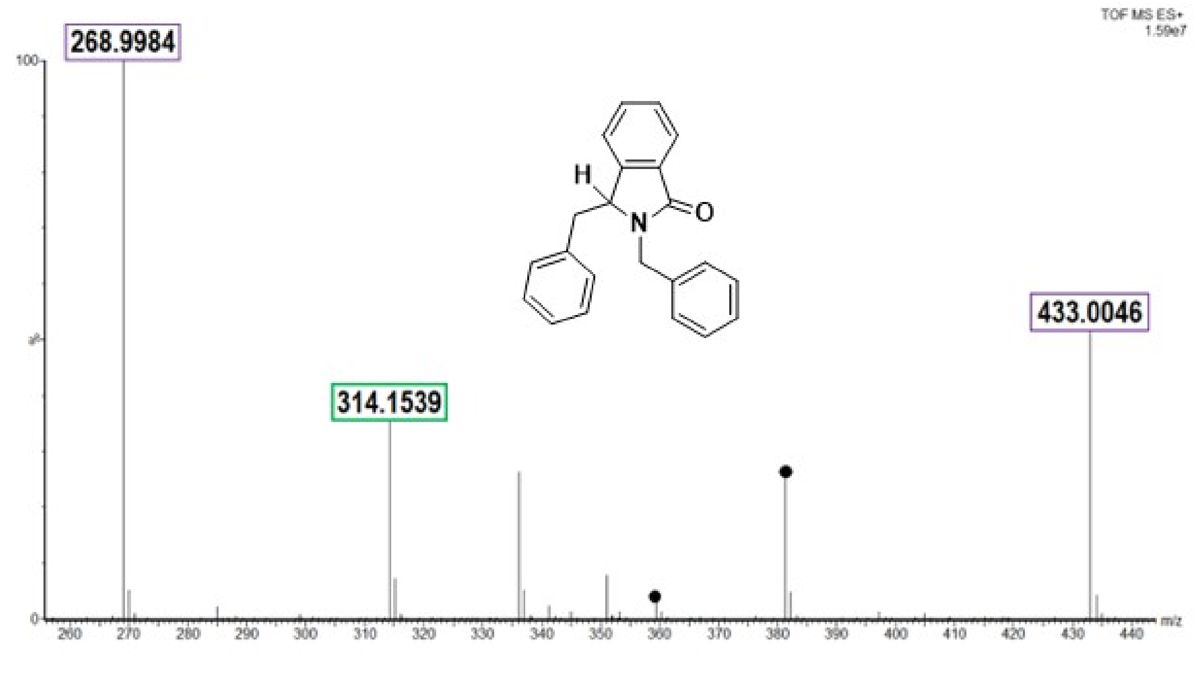

TOF MS ES+
1.59e7
268.9984
314.1539
433.0046
H
N
O
100
%
0
m/z
260 270 280 290 300 310 320 330 340 350 360 370 380 390 400 410 420 430 440

Synthesis of 3-Benzyl-2-(4-chlorobenzyl)isoindolin-1-one (1d)

Following the procedure for the synthesis of 3-alkylisoindolin-1-ones **1** using (*Z*)-3-benzylideneisobenzofuran-1(3*H*)-one **2a** (111 mg, 0.5 mmol, 1 equiv.) and 4-chlorobenzylamine (0.122 mL, 1 mmol, 2 equiv.) with purification using column chromatography (*n*-hexane/ethyl acetate, 9:1), afforded the desired compound **1d.**

Yellow solid; mp: 108-110°C; yield: 131 mg (75%).

IR (KBr): 3020, 2975, 2925, 2860, 1680, 1603, 1404, 1072, 879, 702 cm^{-1}.

^{1}H-NMR ($CDCl_3$, 500 MHz): δ = 7.85-7.83 (m, 1H, CH_{Ar}), 7.45-7.39 (m, 2H, CH_{Ar}), 7.31-7.24 (m, 5H, CH_{Ar}), 7.12-7.11 (m, 2H, CH_{Ar}), 7.02-7.00 (m, 2H, CH_{Ar}), 6.91 (d, *J* = 8.0 Hz, 1H, CH_{Ar}), 5.36 (d, *J* = 15.0 Hz, 1H, CH_2), 4.57 (dd, *J* = 7.5 and 5.0 Hz, 1H, CH), 4.18 (d, *J* = 15.0 Hz, 1H, CH_2), 3.30 (dd, *J* = 14.0 and 5.5 Hz, 1H, CH_2), 2.86 (dd, *J* = 14.0 and 7.5 Hz, 1H, CH_2).

^{13}C-NMR ($CDCl_3$, 125 MHz): δ = 168.6 (C=O), 145.2 (C_{Ar}), 136.2 (C_{Ar}), 135.8 (C_{Ar}), 131.9 (C_{Ar}), 131.4 (CH_{Ar}), 129.7 (C_{Ar}), 129.5$_9$ (2CH_{Ar}), 129.5$_6$ (2CH_{Ar}), 129.1 (2CH_{Ar}), 128.7 (2CH_{Ar}), 128.4 (CH_{Ar}), 127.2 (CH_{Ar}), 124.0 (CH_{Ar}), 123.1 (CH_{Ar}), 59.8 (CH), 43.8 (CH_2), 38.9 (CH_2).

HR-MS (ESI): m/z $[M+H]^+$ cald for $C_{22}H_{19}ClNO^+$: 348.1150, found: 348.1146.

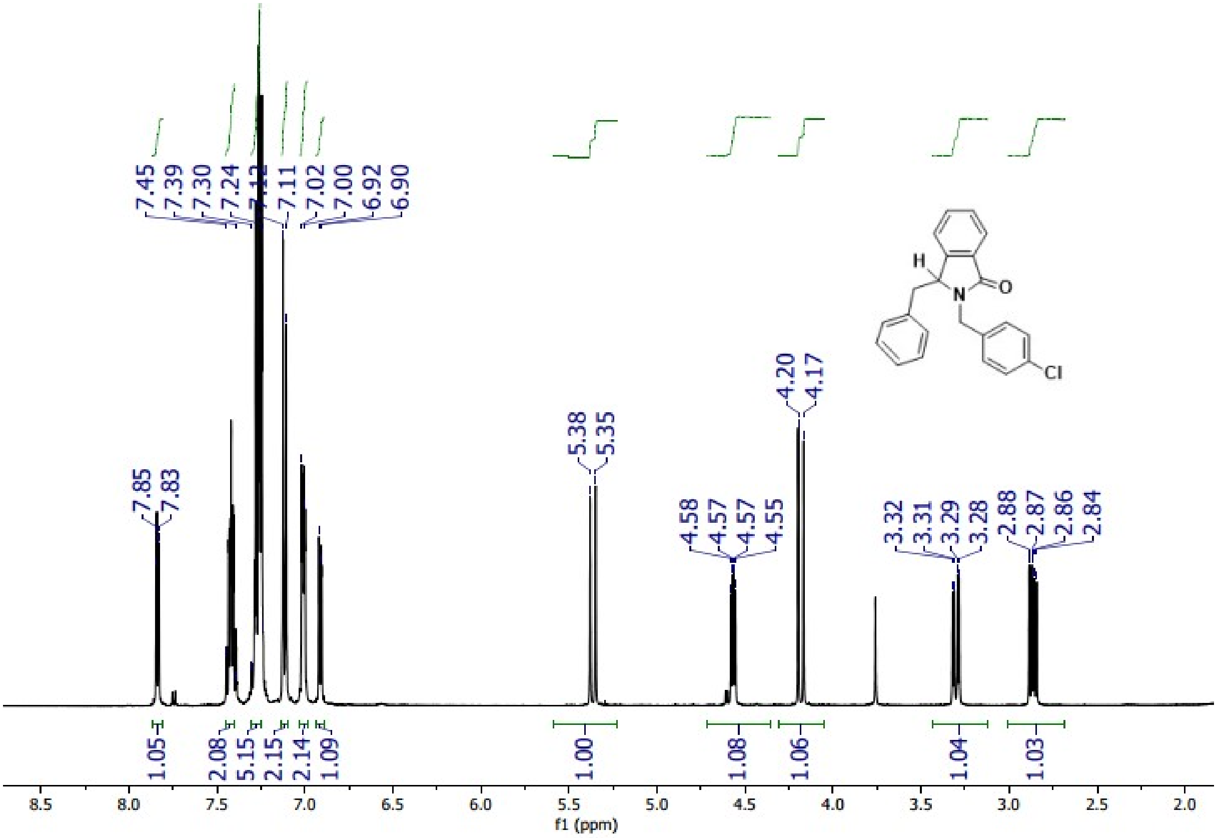

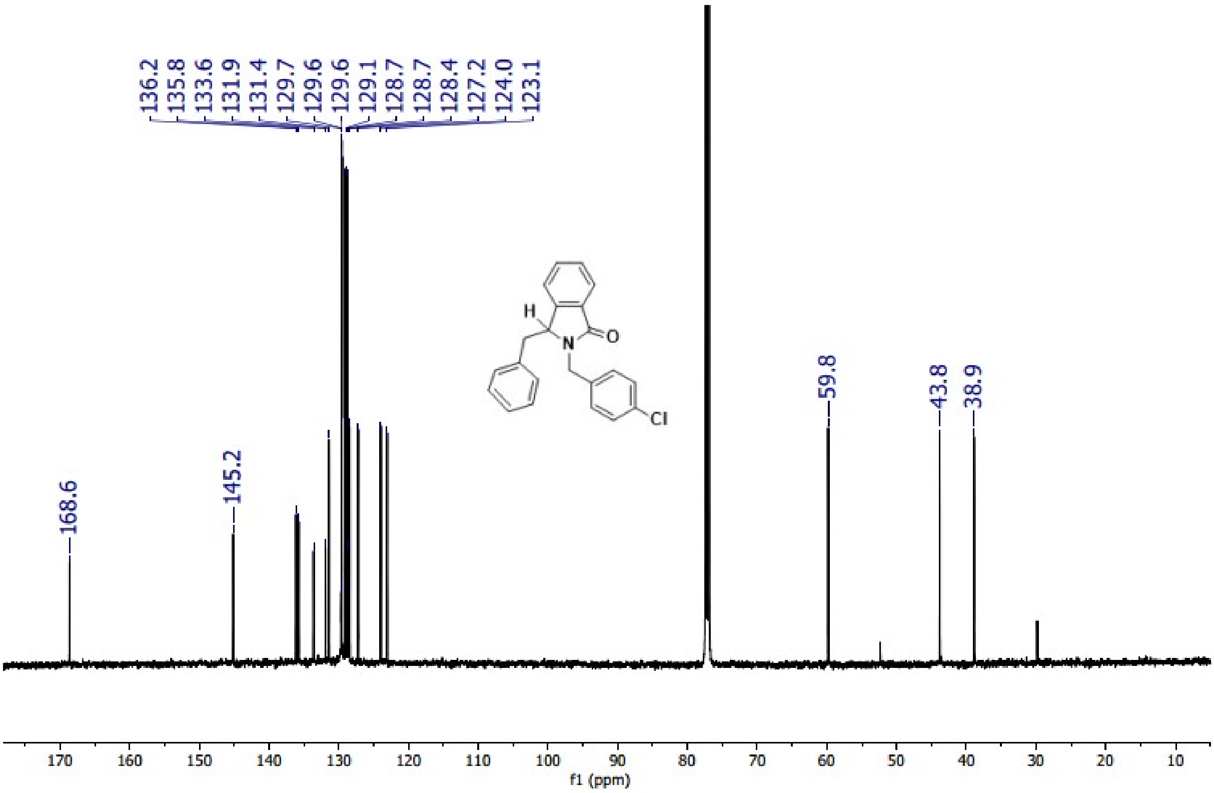
136.2
135.8
133.6
131.9
131.4
129.7
129.6
129.6
129.1
128.7
128.7
128.4
127.2
124.0
123.1
168.6
145.2
59.8
43.8
38.9
H
N
O
Cl
170
160
150
140
130
120
110
100
90
80
70
60
50
40
30
20
10
f1 (ppm)

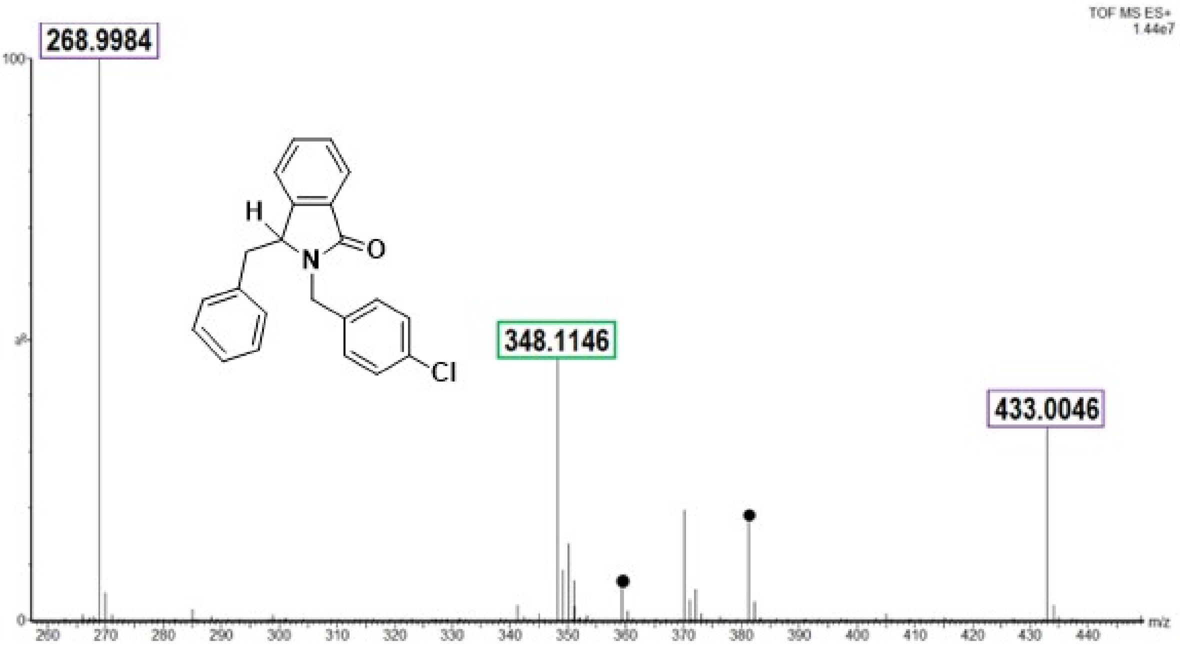
TOF MS ES+
1.44e7
268.9984
348.1146
433.0046
H
N
O
Cl
100
%
0
260
270
280
290
300
310
320
330
340
350
360
370
380
390
400
410
420
430
440
m/z

Synthesis of 3-Benzyl-2-(4-fluorobenzyl)isoindolin-1-one (1e)

Following the procedure for the synthesis of 3-alkylisoindolin-1-ones **1** using (*Z*)-3-benzylideneisobenzofuran-1(3*H*)-one **2a** (111 mg, 0.5 mmol, 1 equiv.) and 4-fluorobenzylamine (0.114 mL, 1 mmol, 2 equiv.) with purification using column chromatography (*n*-hexane/ethyl acetate, 9:1), afforded the desired compound **1e.**
Yellow solid; mp: 108-112°C; yield: 119 mg (72%).

IR (KBr): 3024, 2975, 2924, 2865, 1682, 1603, 1404, 1219, 1072, 764, 702 cm^{-1}.

^{1}H-NMR ($CDCl_3$, 500 MHz): δ = 7.85-7.83 (m, 1H, CH_{Ar}), 7.44-7.39 (m, 2H, CH_{Ar}), 7.28-7.23 (m, 3H, CH_{Ar}), 7.18-7.15 (m, 2H, CH_{Ar}), 7.02-6.98 (m, 4H, CH_{Ar}), 6.90 (d, *J* = 8.0 Hz, 1H, CH_{Ar}), 5.37 (d, *J* = 15.0 Hz, 1H, C*H*$_2$), 4.56 (dd, *J* = 8.0 and 5.0 Hz, 1H, CH), 4.19 (d, *J* = 15.0 Hz, 1H, C*H*$_2$), 3.32 (dd, *J* = 14.0 and 5.5 Hz, 1H, C*H*$_2$), 2.86 (dd, *J* = 14.0 and 7.5 Hz, 1H, C*H*$_2$).

^{13}C-NMR ($CDCl_3$, 125 MHz): δ = 168.6 (C=O), 162.4 (d, *J* = 245 Hz, C_{Ar}), 145.2 (C_{Ar}), 136.2 (C_{Ar}), 133.0 (d, *J* = 3.0 Hz, C_{Ar}), 132.0 (C_{Ar}), 131.4 (CH_{Ar}), 129.9 (d, *J* = 8.0 Hz, 2CH_{Ar}), 129.6 (2CH_{Ar}), 128.7 (2CH_{Ar}), 128.4 (CH_{Ar}), 127.2 (CH_{Ar}), 124.0 (CH_{Ar}), 123.1 (CH_{Ar}), 115.7 (d, *J* = 21.5 Hz,2CH_{Ar}), 59.7 (CH), 43.7 (CH_2), 38.8 (CH_2).

^{13}F-NMR ($CDCl_3$, 470 MHz): δ = -114.7.

HR-MS (ESI): m/z $[M+H]^+$ cald for $C_{22}H_{19}FNO^+$: 332.1445, found: 332.1443.

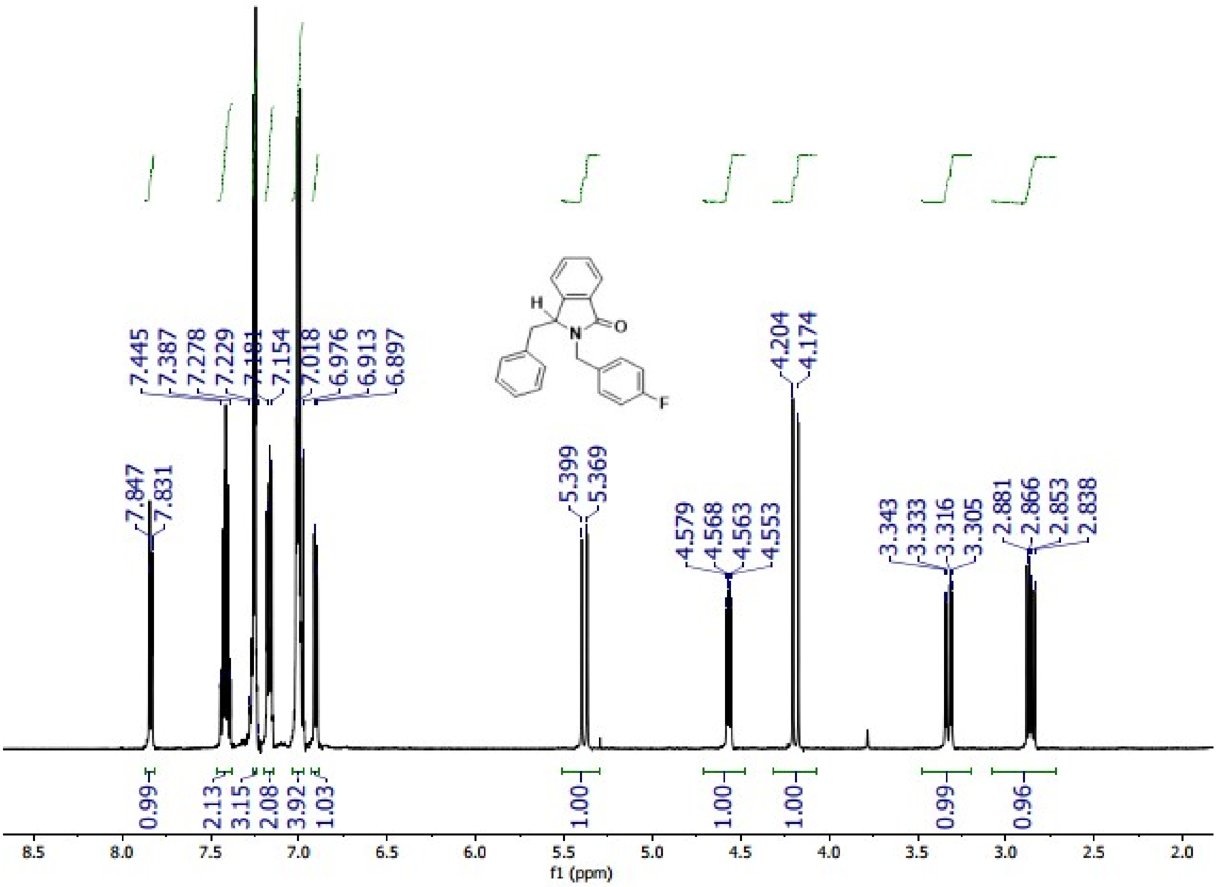

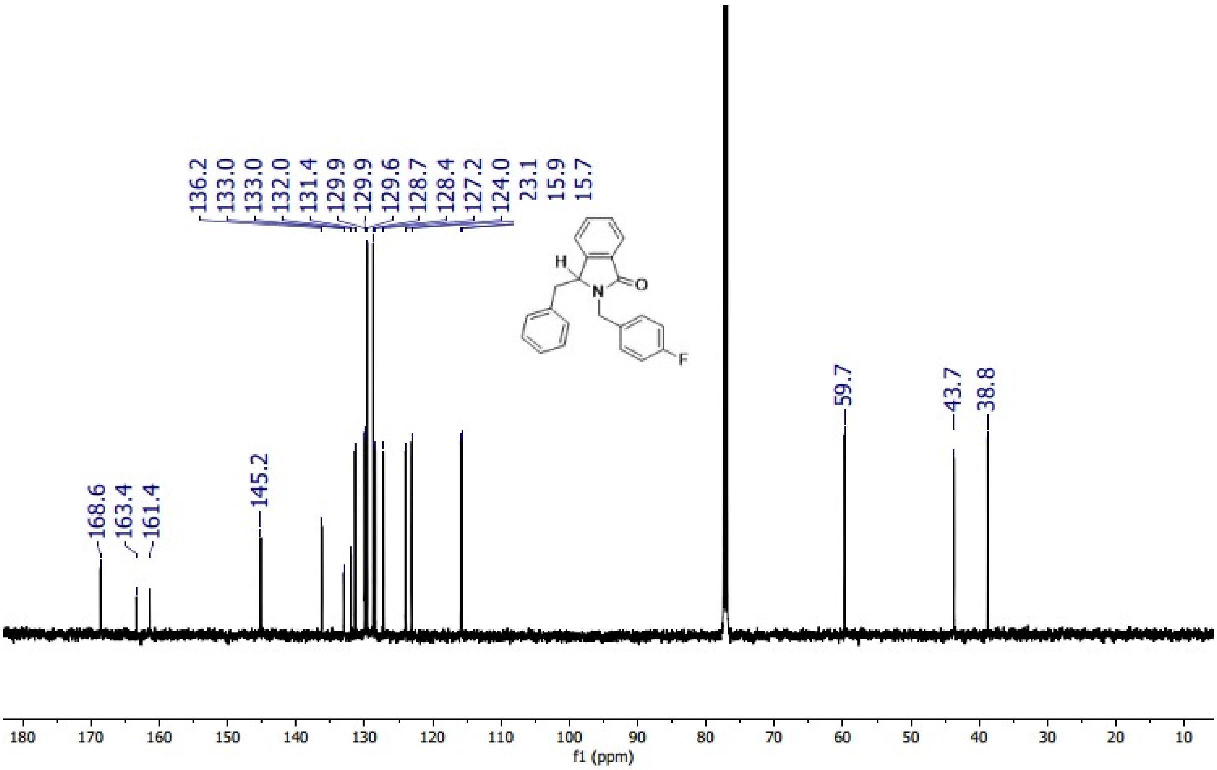
136.2
133.0
133.0
132.0
131.4
129.9
129.9
129.6
128.7
128.4
127.2
124.0
23.1
15.9
15.7
168.6
163.4
161.4
145.2
59.7
43.7
38.8
H
N
O
F
180 170 160 150 140 130 120 110 100 90 80 70 60 50 40 30 20 10
f1 (ppm)

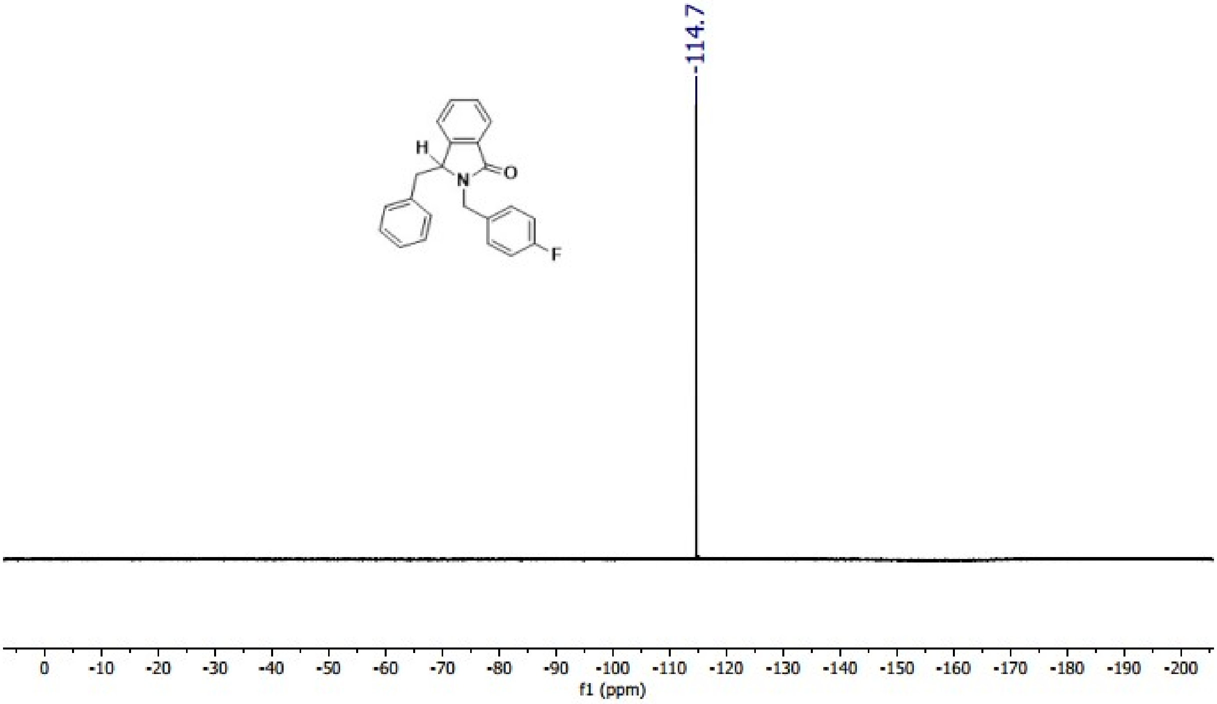
-114.7
H
N
O
F
0 -10 -20 -30 -40 -50 -60 -70 -80 -90 -100 -110 -120 -130 -140 -150 -160 -170 -180 -190 -200
f1 (ppm)

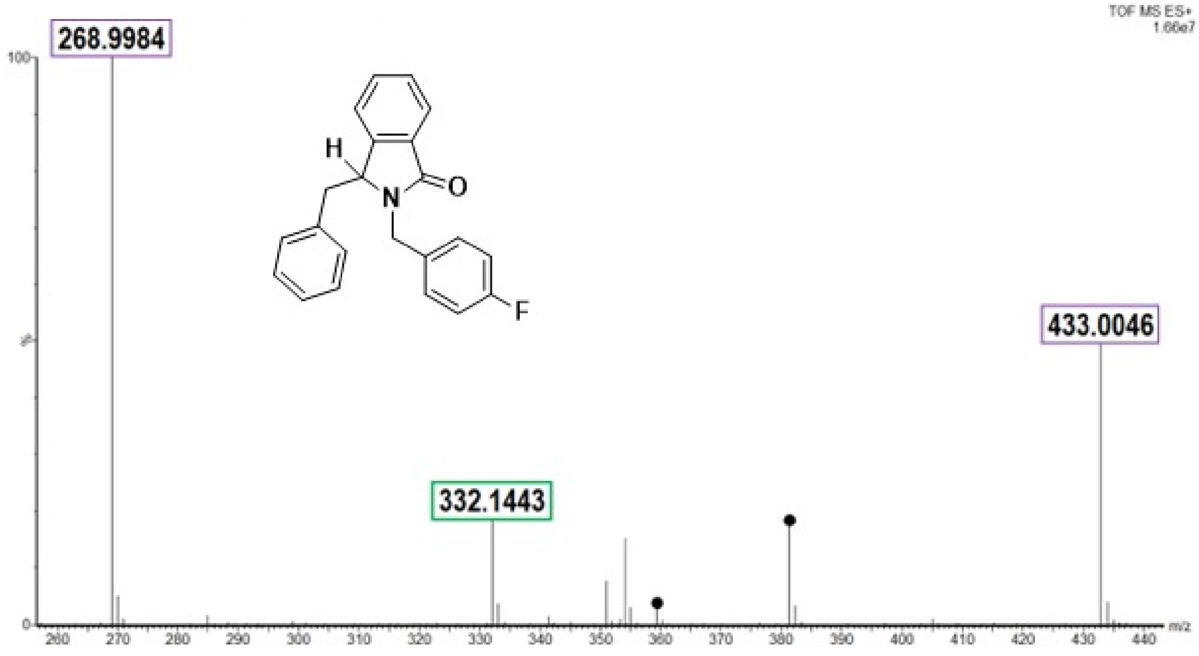
TOF MS ES+
1.66e7
268.9984
332.1443
433.0046
H
N
O
F
100
%
0
m/z

Synthesis of 3-Benzyl-2-(4-(trifluoromethyl)benzyl)isoindolin-1-one (1f)

Following the procedure for the synthesis of 3-alkylisoindolin-1-ones **1** using (*Z*)-3-benzylideneisobenzofuran-1(3*H*)-one **2a** (111 mg, 0.5 mmol, 1 equiv.) and 4-(trifluoromethyl) benzylamine (0.143 mL, 1 mmol, 2 equiv.) with purification using column chromatography (*n*-hexane/ethyl acetate, 9:1), afforded the desired compound **1f.**
Yellow oil; yield: 160 mg (84%).

IR (nujol): 3022, 2974, 2924, 2860, 1683, 1404, 1217, 1072, 764, 702 cm^{-1}.

^{1}H-NMR ($CDCl_3$, 500 MHz): δ = 7.87-7.85 (m, 1H, CH_{Ar}), 7.55 (d, *J* = 8.0 Hz, 2H, CH_{Ar}), 7.46-7.42 (m, 2H, CH_{Ar}), 7.28-7.25 (m, 5H, CH_{Ar}), 7.02-7.00 (m, 2H, CH_{Ar}), 6.95-6.96 (m, 1H, CH_{Ar}), 5.41 (d, *J* = 15.0 Hz, 1H, CH_2), 4.59 (dd, *J* = 7.5 and 5.0 Hz, 1H, CH), 4.28 (d, *J* = 15.0 Hz, 1H, CH_2), 3.28 (dd, *J* = 14.0 and 5.5 Hz, 1H, CH_2), 2.91 (dd, *J* = 14.0 and 7.5 Hz, 1H, CH_2).

^{13}C-NMR ($CDCl_3$, 125 MHz): δ = 168.8 (C=O), 145.2 (C_{Ar}), 141.5 (C_{Ar}), 136.1 (C_{Ar}), 131.8 (C_{Ar}), 131.6 (CH_{Ar}), 130.1 (q, *J* = 32.5 Hz, C_{Ar}), 129.5 ($2CH_{Ar}$), 128.8 ($2CH_{Ar}$), 128.5 (CH_{Ar}), 128.4 ($2CH_{Ar}$), 127.4 (CH_{Ar}), 125.9 (CH_{Ar}), 125.8 (CH_{Ar}), 124.1 (CH_{Ar}), 123.1 (CH_{Ar}), 122.1 (q, *J* = 270.0 Hz, CF_3), 60.0 (CH), 44.1 (CH_2), 39.0 (CH_2).

^{13}F-NMR ($CDCl_3$, 470 MHz): δ = -62.5.

HR-MS (ESI): m/z $[M+H]^+$ cald for $C_{23}H_{19}F_3NO^+$: 382.1413, found: 382.1415.

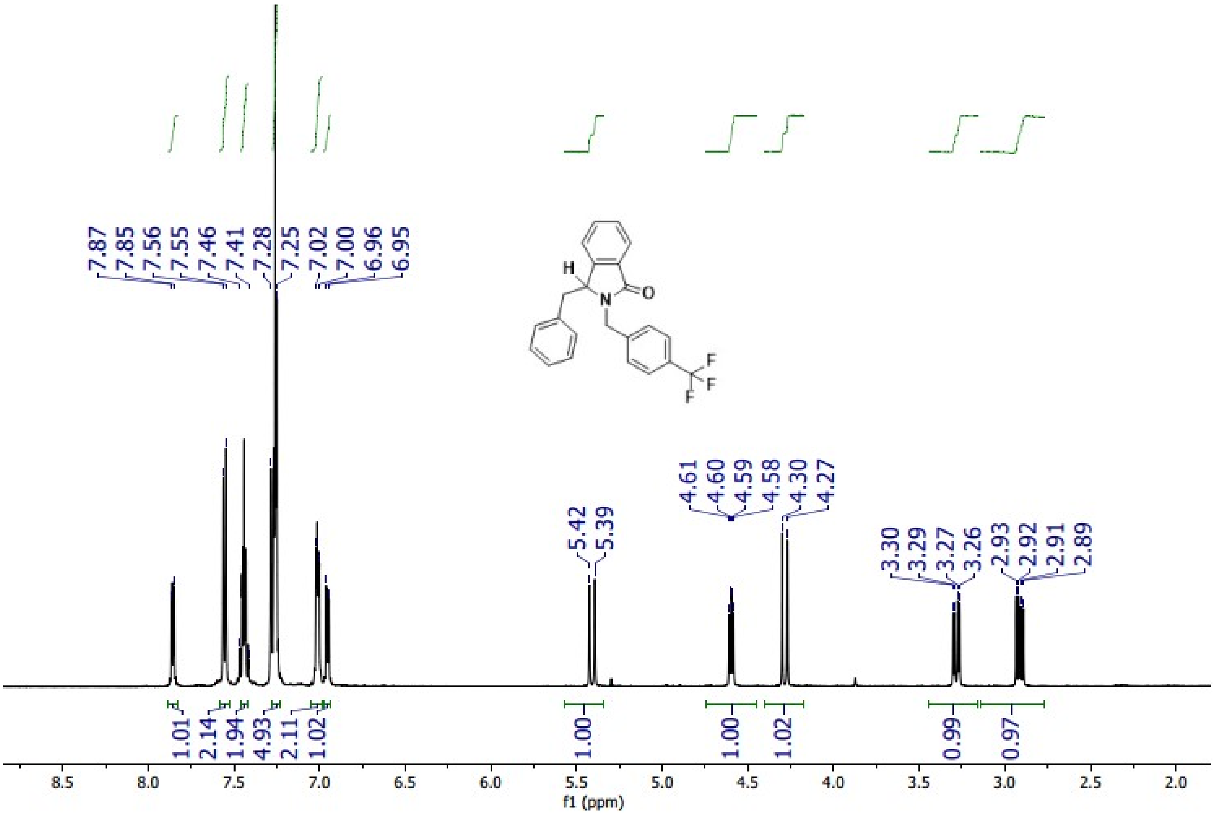

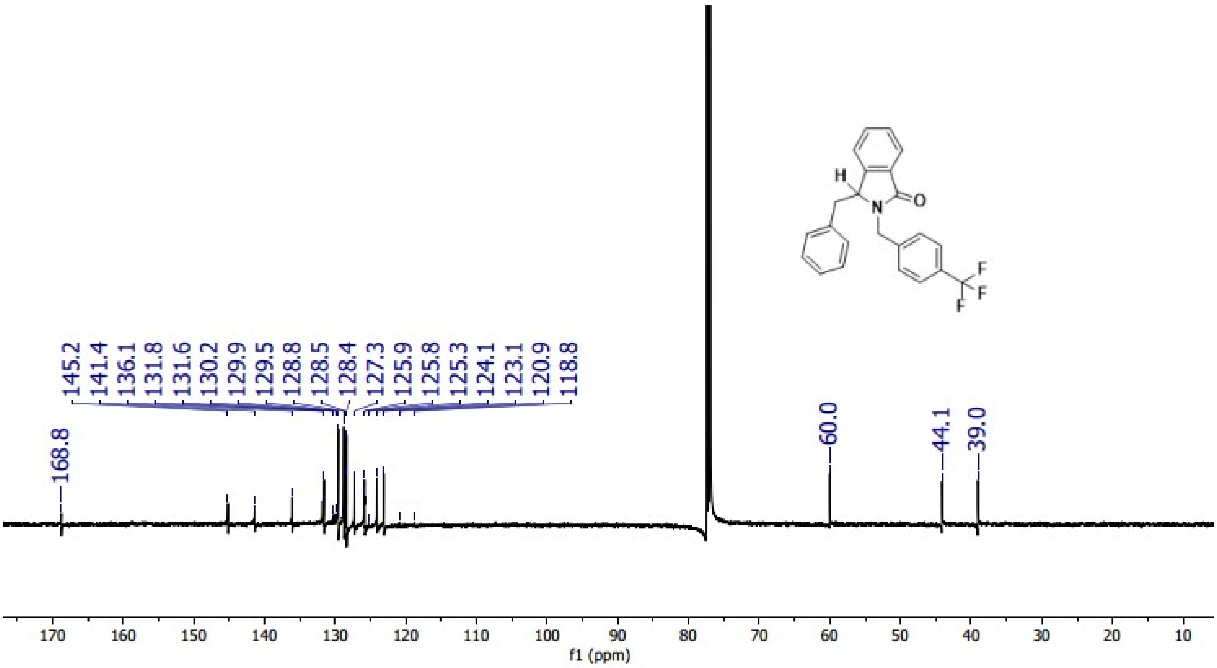
145.2
141.4
136.1
131.8
131.6
130.2
129.9
129.5
128.8
128.5
128.4
127.3
125.9
125.8
125.3
124.1
123.1
120.9
118.8
168.8
60.0
44.1
39.0
H
N
O
F
F
F
170 160 150 140 130 120 110 100 90 80 70 60 50 40 30 20 10
f1 (ppm)

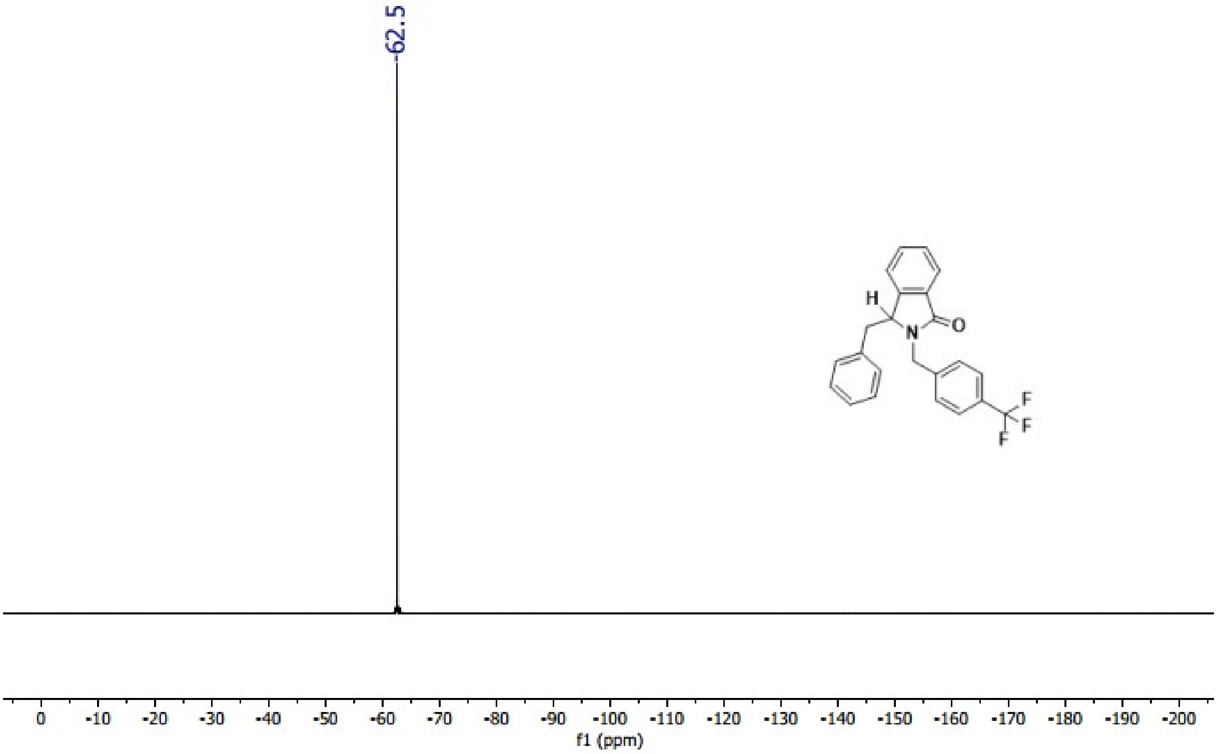
-62.5
H
N
O
F
F
F
0 -10 -20 -30 -40 -50 -60 -70 -80 -90 -100 -110 -120 -130 -140 -150 -160 -170 -180 -190 -200
f1 (ppm)

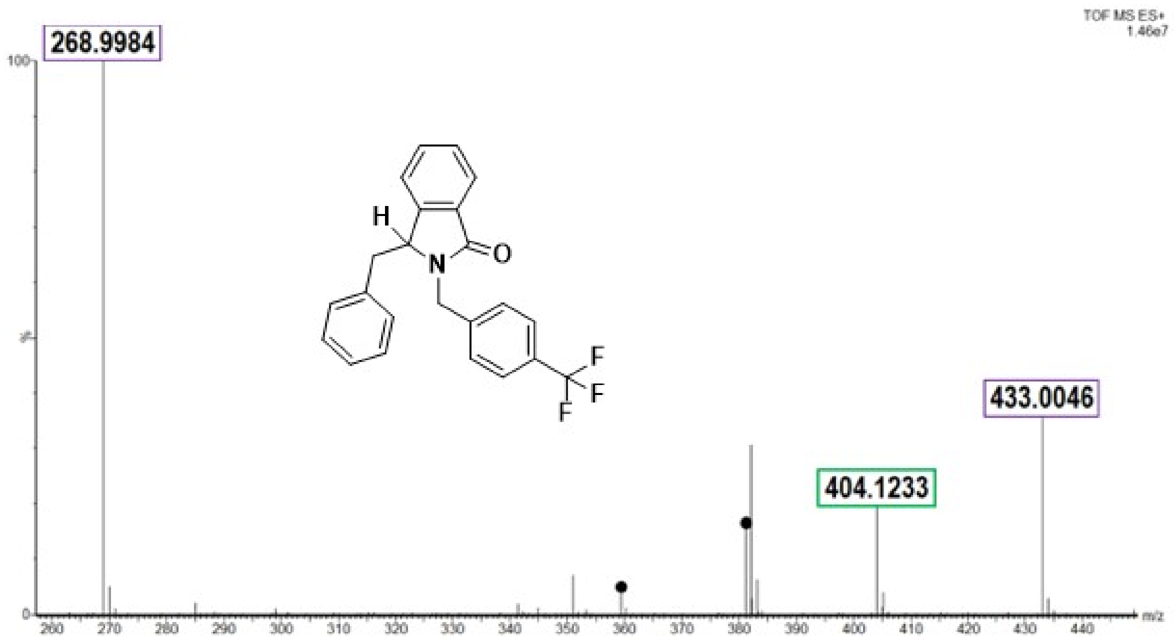

TOF MS ES+
1.46e7
268.9984
H
N
O
F
F
F
433.0046
404.1233
100
%
0
260
270
280
290
300
310
320
330
340
350
360
370
380
390
400
410
420
430
440
m/z

Synthesis of 3-Benzyl-2-(4-methylbenzyl)isoindolin-1-one (1g)

Following the procedure for the synthesis of 3-alkylisoindolin-1-ones **1** using (*Z*)-3-benzylideneisobenzofuran-1(3*H*)-one **2a** (111 mg, 0.5 mmol, 1 equiv.) and 4-methylbenzylamine (0.127 mL, 1 mmol, 2 equiv.) with purification using column chromatography (*n*-hexane/ethyl acetate, 9:1), afforded the desired compound **1g.**
White solid; mp: 118-120°C; yield: 130 mg (79%).

IR (KBr): 3025, 2973, 2924, 2865, 1681, 1404, 1072, 702 cm^{-1}.

^{1}H-NMR ($CDCl_3$, 500 MHz): δ = 7.81 (d, *J* = 8.0 Hz, 1H, CH_{Ar}), 7.41-7.34 (m, 2H, CH_{Ar}), 7.24-7.21 (m, 3H, CH_{Ar}), 7.14-7.10 (m, 4H, CH_{Ar}), 7.00-6.97 (m, 2H, CH_{Ar}), 6.83 (d, *J* = 8.0 Hz, 1H, CH_{Ar}), 5.42 (d, *J* = 15.0 Hz, 1H, CH_2), 4.54 (dd, *J* = 8.0 and 5.0 Hz, 1H, CH), 4.16 (d, *J* = 15.0 Hz, 1H, CH_2), 3.36 (dd, *J* = 14.0 and 5.0 Hz, 1H, CH_2), 2.80 (dd, *J* = 14.0 and 8.0 Hz, 1H, CH_2), 2.32 (s, 3H, CH_3).

^{13}C-NMR ($CDCl_3$, 125 MHz): δ = 168.5 (C=O), 145.2 (C_{Ar}), 137.5 (C_{Ar}), 136.2 (C_{Ar}), 134.3 (C_{Ar}), 132.2 (C_{Ar}), 131.2 (CH_{Ar}), 129.6 ($2CH_{Ar}$), 129.5 ($2CH_{Ar}$), 128.6 ($2CH_{Ar}$), 128.3_3 ($2CH_{Ar}$), 128.3_2 (CH_{Ar}), 127.1 (CH_{Ar}), 123.9 (CH_{Ar}), 123.1 (CH_{Ar}), 59.6 (CH), 44.1 (CH_2), 38.5 (CH_2), 21.3 (CH_3).

HR-MS (ESI): m/z $[M+H]^+$ cald for $C_{23}H_{22}NO^+$: 328.1696, found: 328.1693.

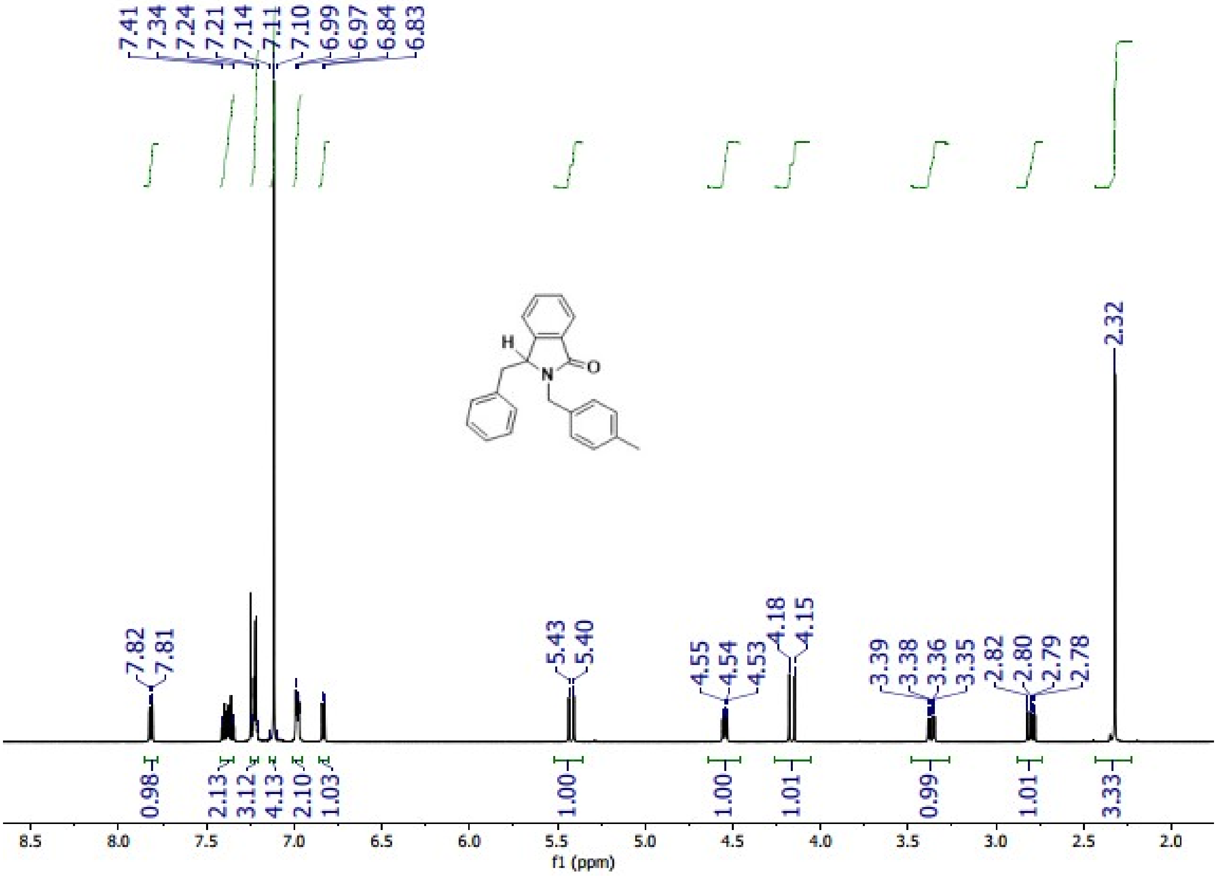

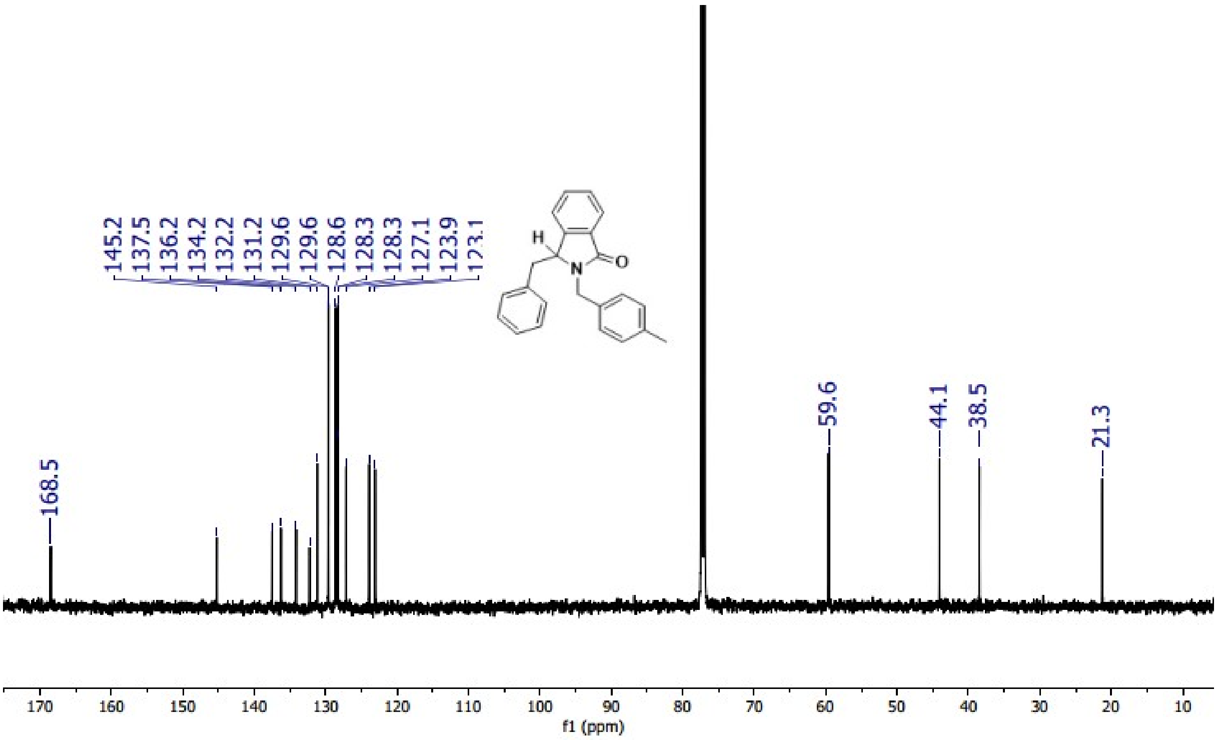

145.2
137.5
136.2
134.2
132.2
131.2
129.6
129.6
128.6
128.3
128.3
127.1
123.9
123.1
168.5
59.6
44.1
38.5
21.3
H
N
O
170
160
150
140
130
120
110
100
90
80
70
60
50
40
30
20
10
f1 (ppm)

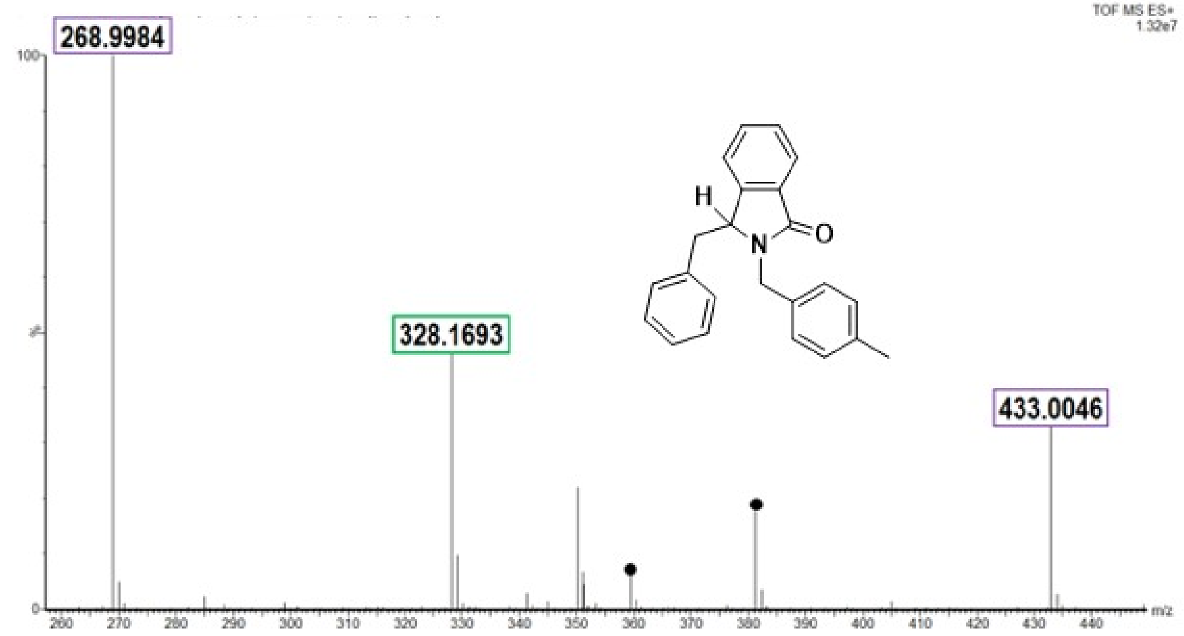

TOF MS ES+
1.32e7
268.9984
328.1693
433.0046
100
%
0
H
N
O
260
270
280
290
300
310
320
330
340
350
360
370
380
390
400
410
420
430
440
m/z

Synthesis of 3-Benzyl-2-(4-methoxybenzyl)isoindolin-1-one (1h)

Following the procedure for the synthesis of 3-alkylisoindolin-1-ones **1** using (*Z*)-3-benzylideneisobenzofuran-1(3*H*)-one **2a** (111 mg, 0.5 mmol, 1 equiv.) and 4-methoxybenzylamine (0.130 mL, 1 mmol, 2 equiv.) with purification using column chromatography (*n*-hexane/ethyl acetate, 9:1), afforded the desired compound **1h.**
White solid; mp: 92-98°C; yield: 148 mg (86%).

IR (KBr): 3025, 2973, 2924, 2860, 1682, 1512, 1404, 1242, 1180, 1072, 756, 702 cm^{-1}.

^{1}H-NMR ($CDCl_3$, 500 MHz): δ = 7.82 (d, *J* = 7.0 Hz, 1H, CH_{Ar}), 7.41 (td, *J* = 7.0 and 1 Hz, 1H, CH_{Ar}), 7.37 (td, *J* = 7.0 and 1 Hz, 1H, CH_{Ar}), 7.25-7.23 (m, 3H, CH_{Ar}), 7.16 (d, *J* = 8.0 Hz, 2H, CH_{Ar}), 7.01 (d, *J* = 8.0 Hz, 1H, CH_{Ar}), 7.00 (d, *J* = 7.0 Hz, 1H, CH_{Ar}), 6.86-6.84 (m, 1H, CH_{Ar}), 6.84 (d, *J* = 8.0 Hz, 2H, CH_{Ar}), 5.41 (d, *J* = 15.0 Hz, 1H, CH_2), 4.55 (dd, *J* = 8.0 and 5.0 Hz, 1H, CH), 4.15 (d, *J* = 15.0 Hz, 1H, CH_2), 3.79 (s, 3H, CH_3) 3.37 (dd, *J* = 13.5 and 5.0 Hz, 1H, CH_2), 2.82 (dd, *J* = 13.5 and 8.0 Hz, 1H, CH_2).

^{13}C-NMR ($CDCl_3$, 125 MHz): δ = 168.5 (C=O), 159.2 (C_{Ar}), 145.2 (C_{Ar}), 136.3 (C_{Ar}), 132.3 (C_{Ar}), 131.2 (CH_{Ar}), 129.6 ($4CH_{Ar}$), 129.3 (C_{Ar}), 128.7 ($2CH_{Ar}$), 128.3 (CH_{Ar}), 127.1 (CH_{Ar}), 123.9 (CH_{Ar}), 123.1 (CH_{Ar}), 114.3 ($2CH_{Ar}$), 59.6 (CH), 55.4 (CH_3), 43.7 (CH_2), 38.5 (CH_2).

HR-MS (ESI): m/z $[M+H]^+$ cald for $C_{23}H_{22}NO_2^+$: 344.1645, found: 344.1645.

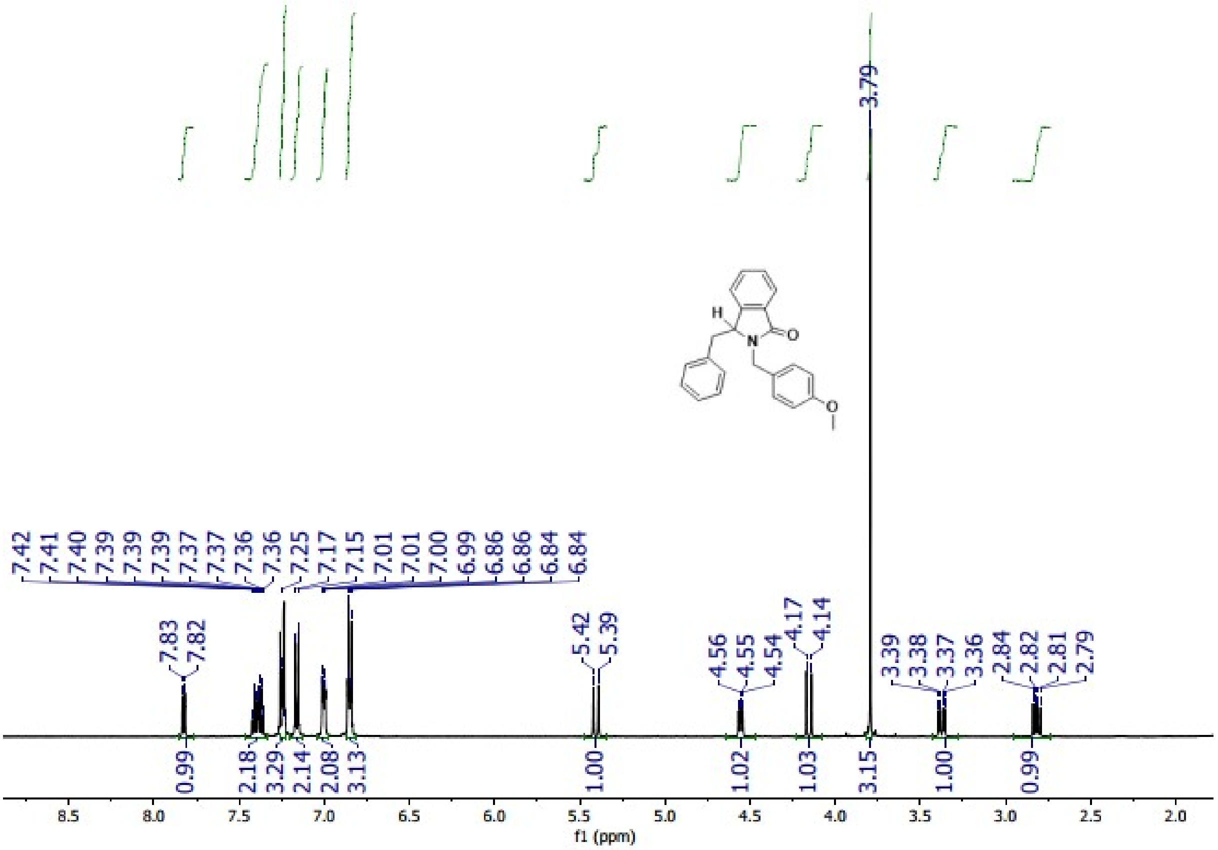

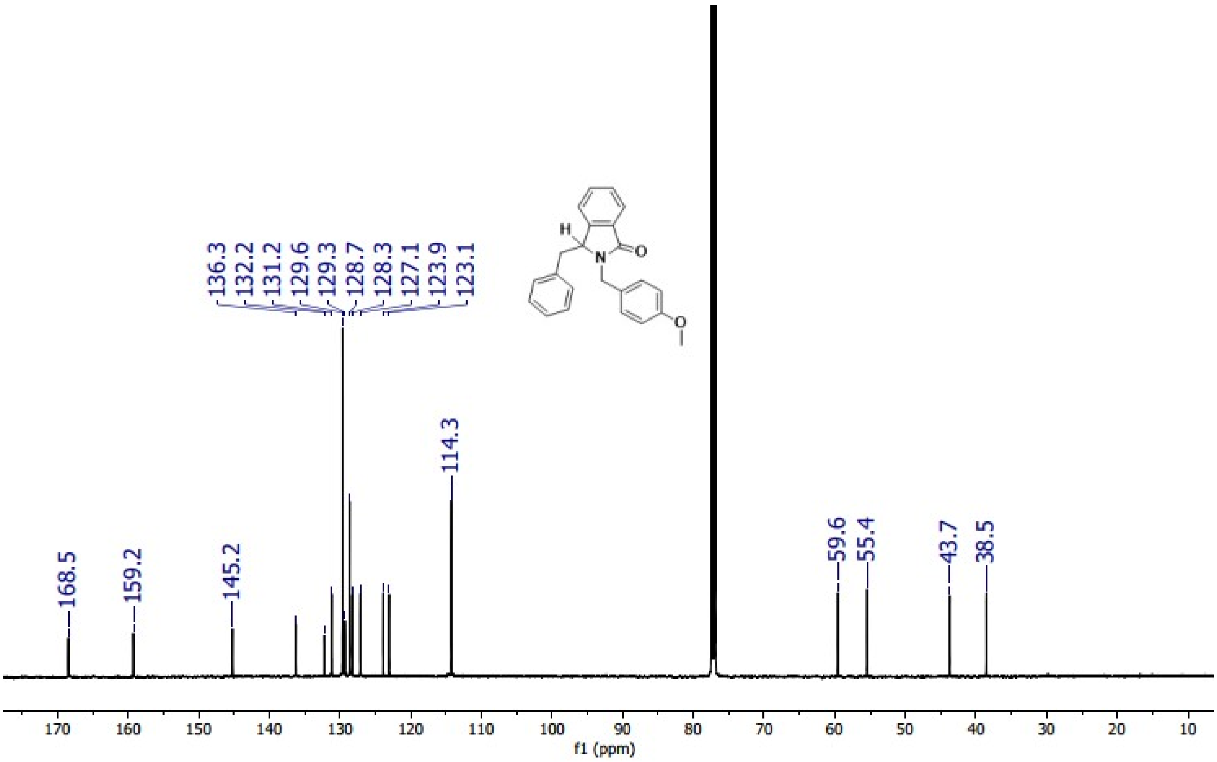

136.3
132.2
131.2
129.6
129.3
128.7
128.3
127.1
123.9
123.1
114.3
168.5
159.2
145.2
59.6
55.4
43.7
38.5
H
N
O
170 160 150 140 130 120 110 100 90 80 70 60 50 40 30 20 10
f1 (ppm)

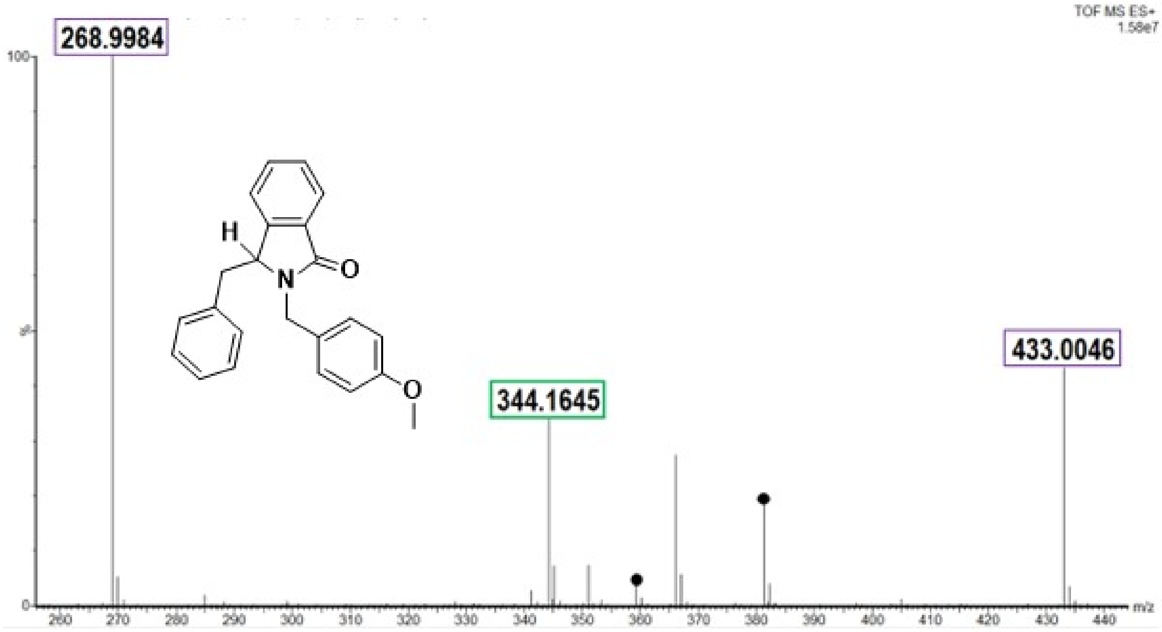

TOF MS ES+
1.58e7
268.9984
344.1645
433.0046
H
N
O
100
0
260 270 280 290 300 310 320 330 340 350 360 370 380 390 400 410 420 430 440
m/z

Synthesis of 3-Benzyl-2-(3-methoxybenzyl)isoindolin-1-one (1i)

Following the procedure for the synthesis of 3-alkylisoindolin-1-ones **1** using (*Z*)-3-benzylideneisobenzofuran-1(3*H*)-one **2a** (111 mg, 0.5 mmol, 1 equiv.) and 3-methoxybenzylamine (0.128 mL, 1 mmol, 2 equiv.) with purification using column chromatography (*n*-hexane/ethyl acetate, 9:1), afforded the desired compound **1i.**
Yellow oil; yield: 131 mg (76%).

IR (nujol): 3025, 2973, 2924, 2860, 1682, 1512, 1411, 1257, 1049, 702 cm^{-1}.

^{1}H-NMR ($CDCl_3$, 500 MHz): δ = 7.83 (d, *J* = 6.5 Hz, 1H, CH_{Ar}), 7.42 (td, *J* = 6.5 and 1 Hz, 1H, CH_{Ar}), 7.38 (td, *J* = 6.5 and 1 Hz, 1H, CH_{Ar}), 7.25-7.22 (m, 4H, CH_{Ar}), 7.00 (dd, *J* = 7.5 and 2 Hz, 2H, CH_{Ar}), 6.87 (d, *J* = 6.5 Hz, 1H, CH_{Ar}), 6.83 (d, *J* = 2 Hz, 1H, CH_{Ar}), 6.81 (d, *J* = 2 Hz, 1H, CH_{Ar}), 6.76 (t, *J* = 2 Hz, 1H, CH_{Ar}), 5.43 (d, *J* = 15.0 Hz, 1H, C*H*$_2$), 4.60 (dd, *J* = 8.0 and 5.0 Hz, 1H, CH), 4.19 (d, *J* = 15.0 Hz, 1H, C*H*$_2$), 3.77 (s, 3H, CH_3), 3.37 (dd, *J* = 14.0 and 5.0 Hz, 1H, C*H*$_2$), 2.83 (dd, *J* = 14.0 and 8.0 Hz, 1H, C*H*$_2$).

^{13}C-NMR ($CDCl_3$, 125 MHz): δ = 168.5 (C=O), 160.1 (C_{Ar}), 145.2 (C_{Ar}), 138.8 (C_{Ar}), 136.2 (C_{Ar}), 132.1 (C_{Ar}), 131.2 (CH_{Ar}), 129.9 (CH_{Ar}), 129.6 ($2CH_{Ar}$), 128.7 ($2CH_{Ar}$), 128.3 (CH_{Ar}), 127.1 (CH_{Ar}), 124.0 (CH_{Ar}), 123.1 (CH_{Ar}), 120.6 (CH_{Ar}), 113.8 (CH_{Ar}), 113.3 (CH_{Ar}), 59.7 (CH), 55.4 (CH_3), 44.3 (CH_2), 38.6 (CH_2).

HR-MS (ESI): m/z $[M+H]^+$ cald for $C_{23}H_{22}NO_2^+$: 344.1645, found: 344.1646.

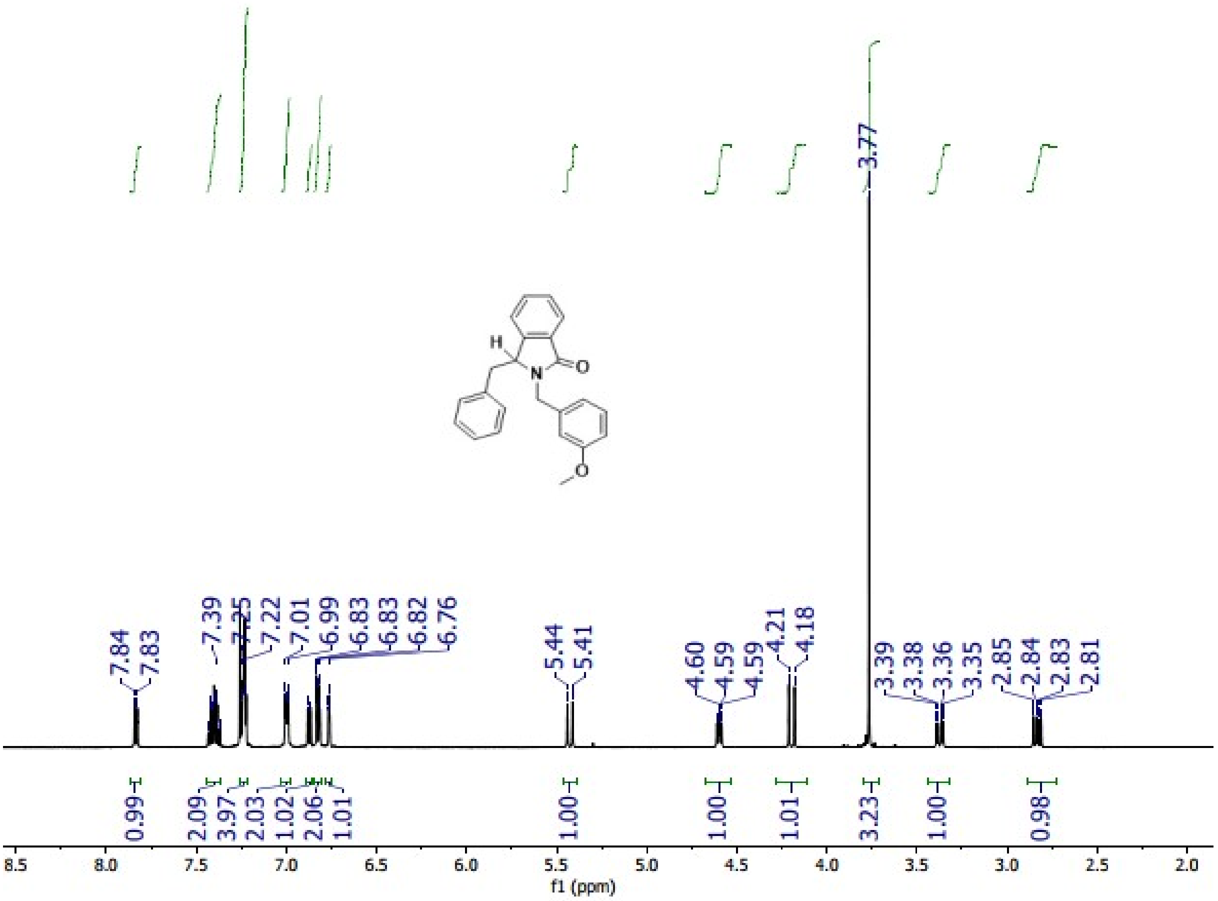

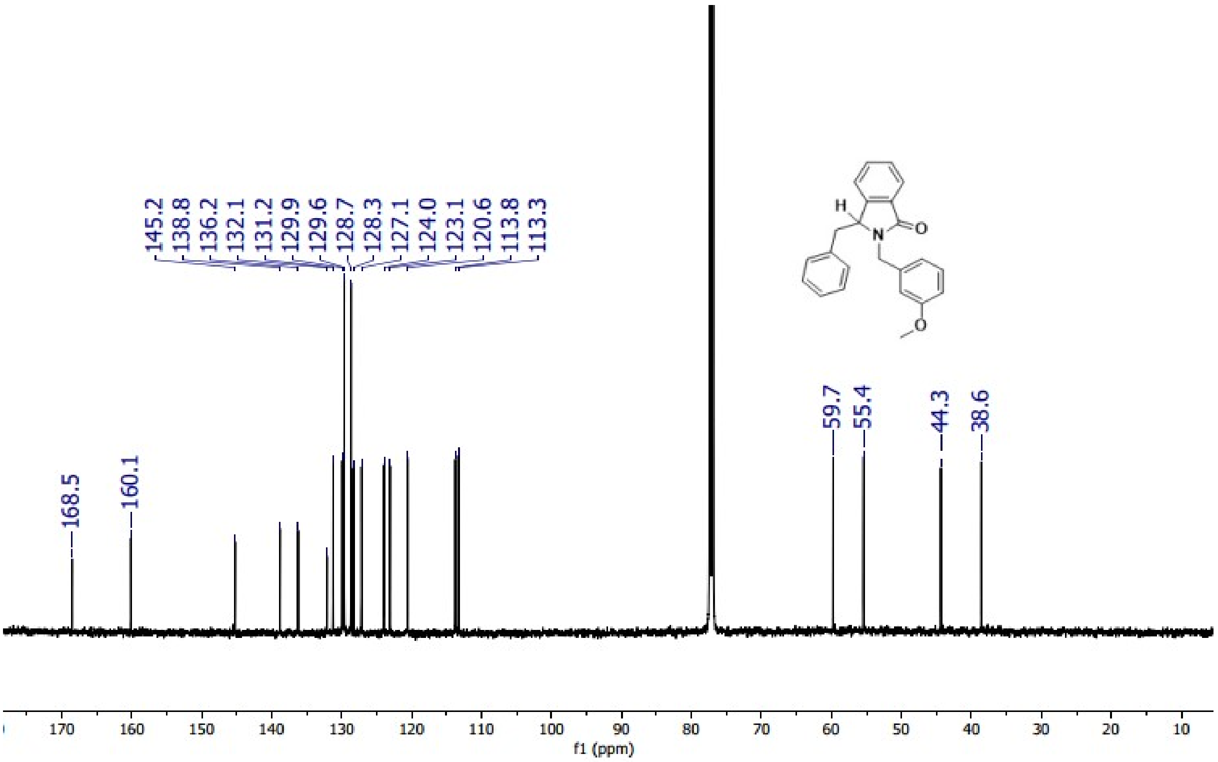
145.2
138.8
136.2
132.1
131.2
129.9
129.6
128.7
128.3
127.1
124.0
123.1
120.6
113.8
113.3
168.5
160.1
59.7
55.4
44.3
38.6
H
N
O
170
160
150
140
130
120
110
100
90
80
70
60
50
40
30
20
10
f1 (ppm)

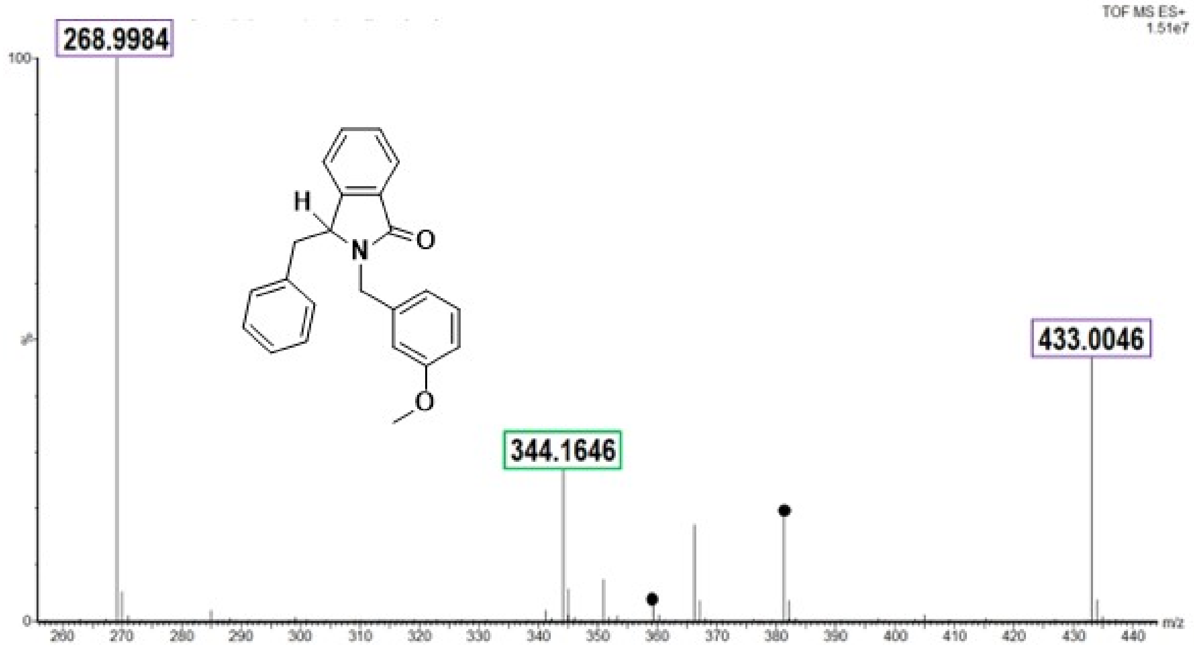
TOF MS ES+
1.51e7
268.9984
344.1646
433.0046
100
%
0
H
N
O
O
260
270
280
290
300
310
320
330
340
350
360
370
380
390
400
410
420
430
440
m/z

Synthesis of 3-Benzyl-2-(2-methoxybenzyl)isoindolin-1-one (1j)

Following the procedure for the synthesis of 3-alkylisoindolin-1-ones **1** using (*Z*)-3-benzylideneisobenzofuran-1(3*H*)-one **2a** (111 mg, 0.5 mmol, 1 equiv.) and 2-methoxybenzylamine (0.130 mL, 1 mmol, 2 equiv.) with purification using column chromatography (*n*-hexane/ethyl acetate, 9:1), afforded the desired compound **1j.**
Yellow oil; yield: 130 mg (76%).

IR (nujol): 3032, 2973, 2924, 2860, 1682, 1412, 1103, 756, 702 cm^{-1}.

^{1}H-NMR ($CDCl_3$, 500 MHz): δ = 7.77 (d, *J* = 7.0 Hz, 1H, CH_{Ar}), 7.37-7.32 (m, 2H, CH_{Ar}), 7.28-7.20 (m, 5H, CH_{Ar}), 6.99-6.98 (m, 2H, CH_{Ar}), 6.91-6.90 (m, 2H, CH_{Ar}), 6.78 (d, *J* = 7 Hz, 1H, CH_{Ar}), 5.23 (d, *J* = 15.0 Hz, 1H, CH_2), 4.59 (m, 2H, CH and CH_2), 3.87 (s, 3H, CH_3) 3.56-3.53 (m, 1H, CH_2), 2.80-2.75 (m, 1H, CH_2).

^{13}C-NMR ($CDCl_3$, 125 MHz): δ = 168.6 (C=O), 157.5 (C_{Ar}), 145.3 (C_{Ar}), 136.4 (C_{Ar}), 132.4 (C_{Ar}), 130.9 (CH_{Ar}), 130.5 (CH_{Ar}), 129.7 (2CH_{Ar}), 129.1 (CH_{Ar}), 128.5 (2CH_{Ar}), 128.1 (CH_{Ar}), 127.0 (CH_{Ar}), 125.5 (C_{Ar}), 123.7 (CH_{Ar}), 123.1 (CH_{Ar}), 121.1 (CH_{Ar}), 110.6 (CH_{Ar}), 60.2 (CH), 55.6 (CH_3), 38.6 (CH_2), 38.1 (CH_2).

HR-MS (ESI): m/z $[M+H]^+$ cald for $C_{23}H_{22}NO_2^+$: 344.1645, found: 344.1642.

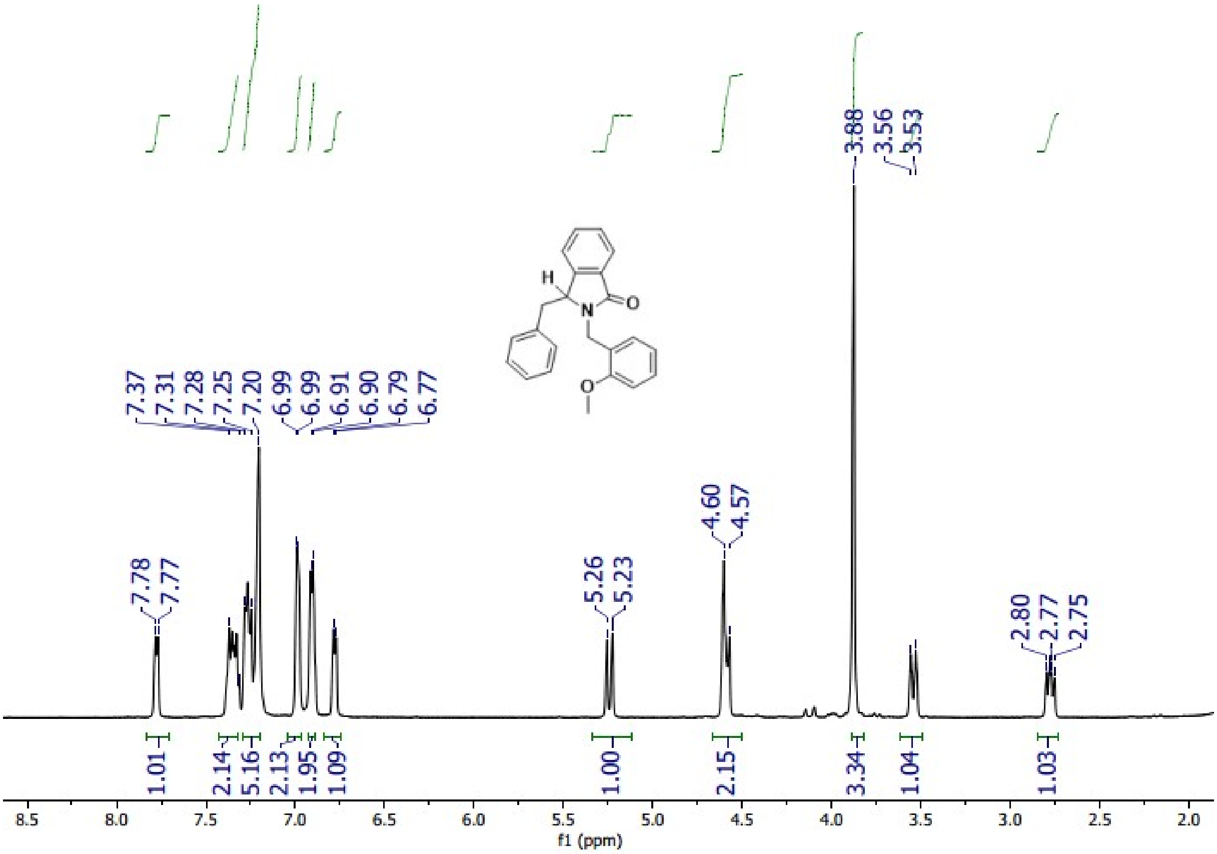

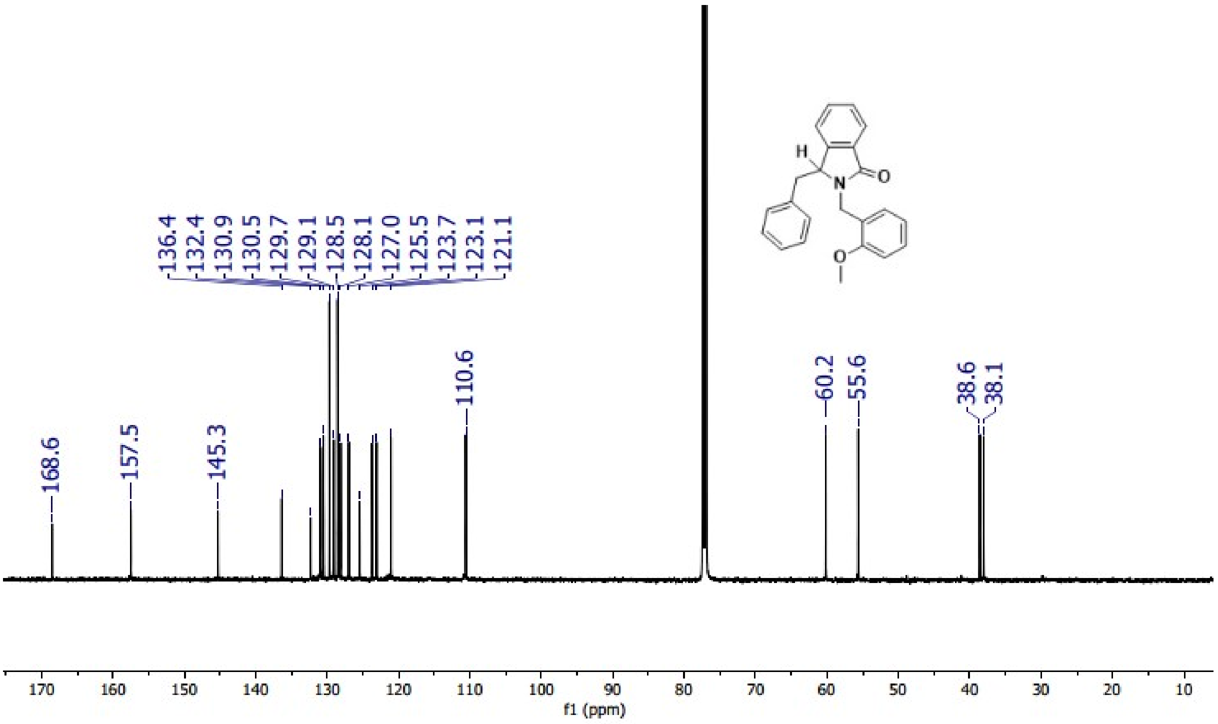

136.4
132.4
130.9
130.5
129.7
129.1
128.5
128.1
127.0
125.5
123.7
123.1
121.1
168.6
157.5
145.3
110.6
60.2
55.6
38.6
38.1
H
N
O
O
170
160
150
140
130
120
110
100
90
80
70
60
50
40
30
20
10
f1 (ppm)

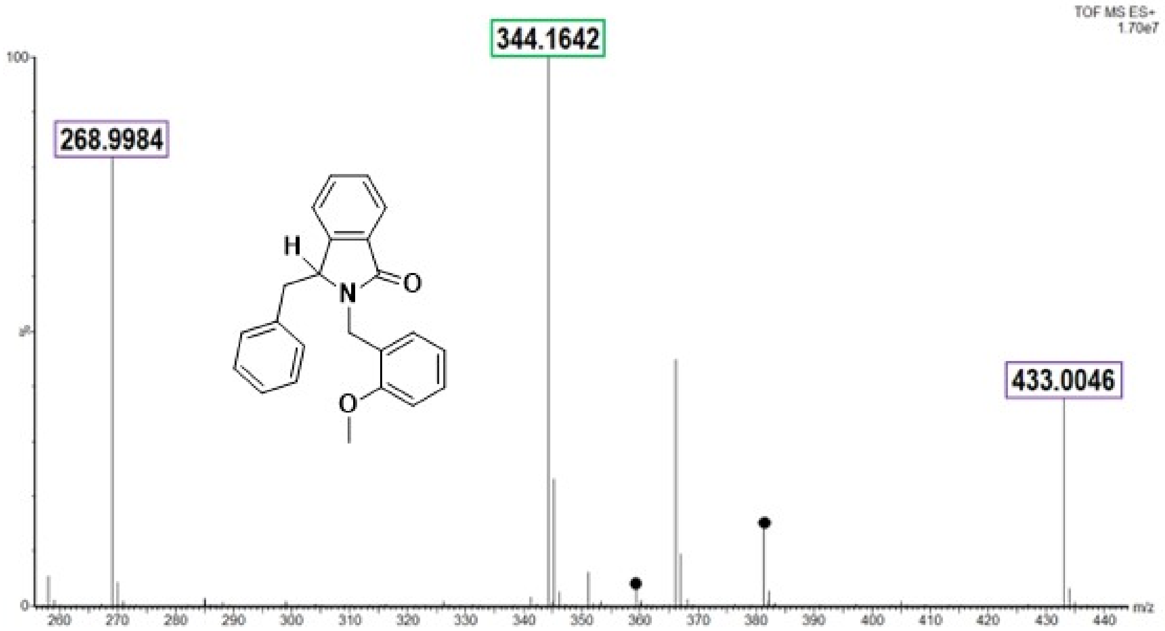

TOF MS ES+
1.70e7
344.1642
268.9984
433.0046
100
%
0
H
N
O
O
260
270
280
290
300
310
320
330
340
350
360
370
380
390
400
410
420
430
440
m/z

Synthesis of 3-Benzyl-2-(furan-2-ylmethyl)isoindolin-1-one (1k)

Following the procedure for the synthesis of 3-alkylisoindolin-1-ones **1** using (*Z*)-3-benzylideneisobenzofuran-1(3*H*)-one **2a** (111 mg, 0.5 mmol, 1 equiv.) and furfurylamine (0.088 mL, 1 mmol, 2 equiv.) with purification using column chromatography (*n*-hexane/ethyl acetate, 9:1), afforded the desired compound **1k.**
Orange solid; mp: 98-101 °C; yield: 120 mg (79%).

IR (KBr): 3032, 2973, 2932, 2860, 1682, 1466, 1420, 1211, 1103, 756, 710 cm^{-1}.

^{1}H-NMR ($CDCl_3$, 500 MHz): δ = 7.81-7.79 (m, 1H, CH_{Ar}), 7.41-7.35 (m, 3H, CH_{Ar}), 7.28-7.22 (m, 3H, CH_{Ar}), 7.06-7.04 (m, 2H, CH_{Ar}), 6.87-6.85 (m, 1H, CH_{Ar}), 6.33 (dd, *J* = 3.5 and 1.0 Hz, 1H, CH_{Ar}), 6.27 (d, *J* = 3.5 Hz, 1H, CH_{Ar}), 5.33 (d, *J* = 16.0 Hz, 1H, CH_2), 4.67 (dd, *J* = 8.0 and 5.0 Hz, 1H, CH), 4.30 (d, *J* = 15.0 Hz, 1H, CH_2), 3.47 (dd, *J* = 14.0 and 5.0 Hz, 1H, CH_2), 2.82 (dd, *J* = 14.0 and 8.5 Hz, 1H, CH_2).

^{13}C-NMR ($CDCl_3$, 125 MHz): δ = 168.2 (C=O), 150.6 (C_{Ar}), 145.2 (C_{Ar}), 142.6 (CH_{Ar}), 136.2 (C_{Ar}), 132.0 (C_{Ar}), 131.3 (CH_{Ar}), 129.7 (2CH_{Ar}), 128.7 (2CH_{Ar}), 128.3 (CH_{Ar}), 127.2 (CH_{Ar}), 123.9 (CH_{Ar}), 123.1 (CH_{Ar}), 110.6 (CH_{Ar}), 108.8 (CH_{Ar}), 60.3 (CH), 38.4 (CH_2), 37.2 (CH_2).

HR-MS (ESI): m/z $[M+H]^+$ cald for $C_{20}H_{18}NO_2^+$: 304.1332, found: 304.1330.

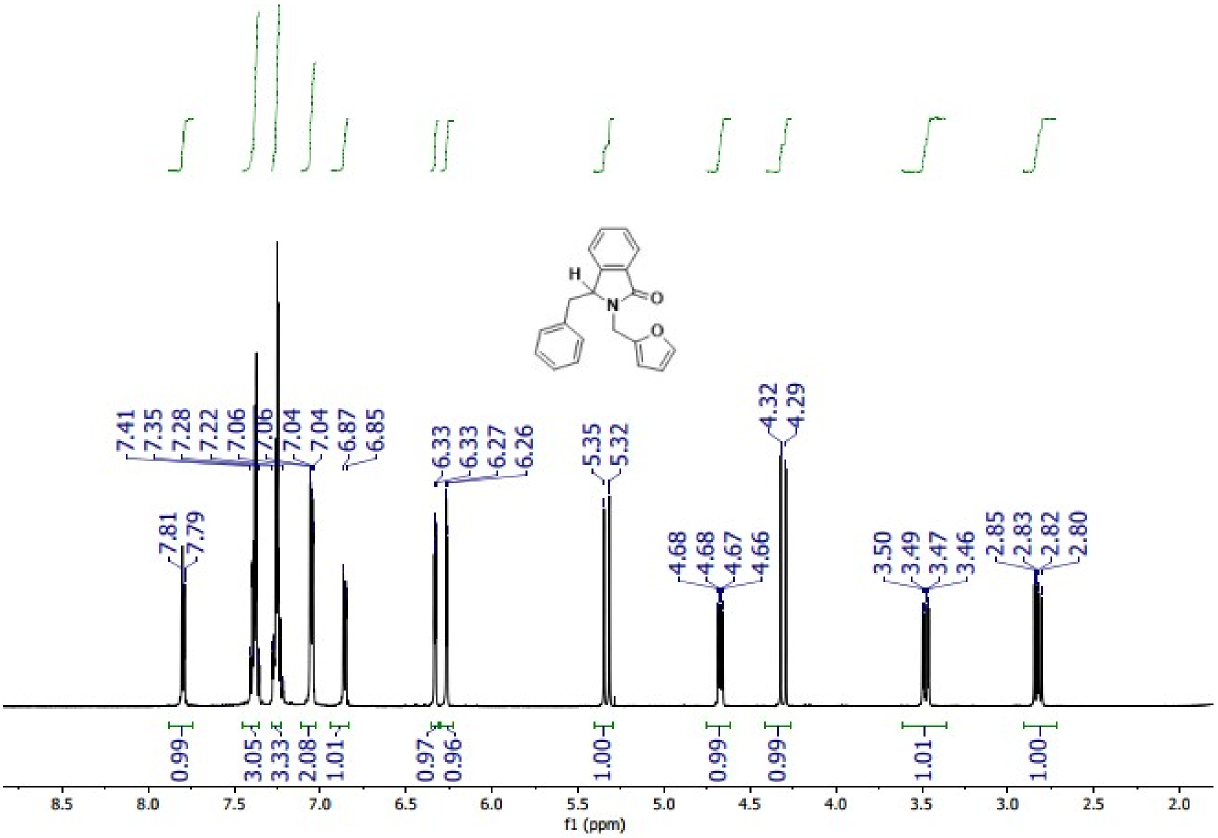

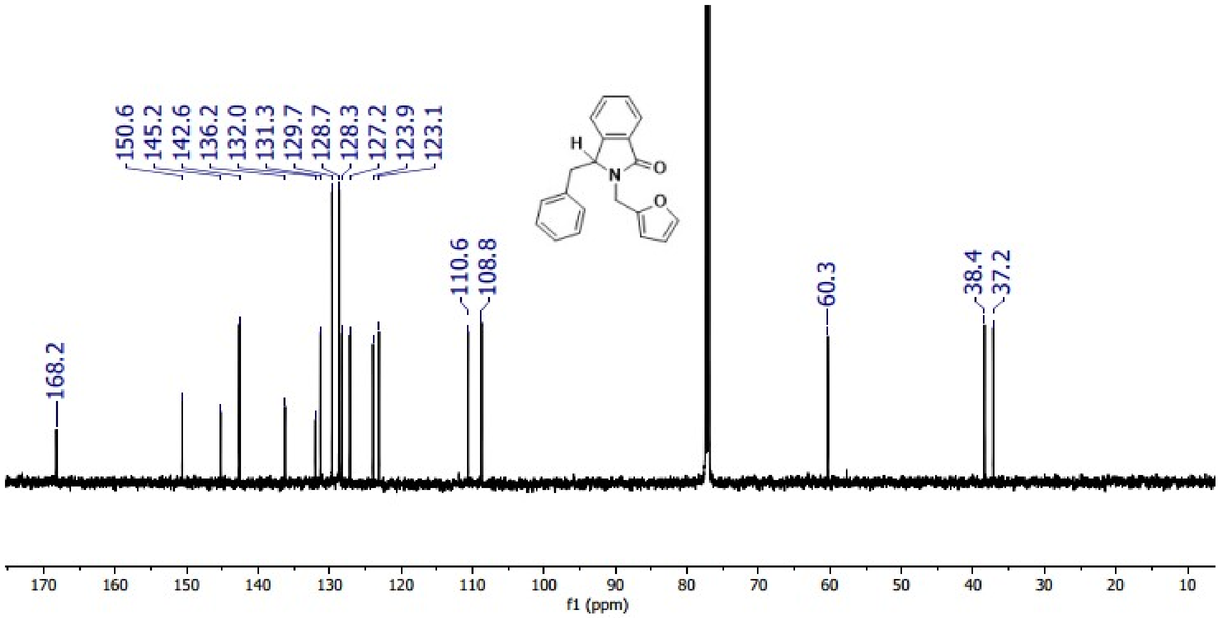
150.6
145.2
142.6
136.2
132.0
131.3
129.7
128.7
128.3
127.2
123.9
123.1
H
N
O
O
110.6
108.8
60.3
38.4
37.2
168.2
170
160
150
140
130
120
110
100
90
80
70
60
50
40
30
20
10
f1 (ppm)

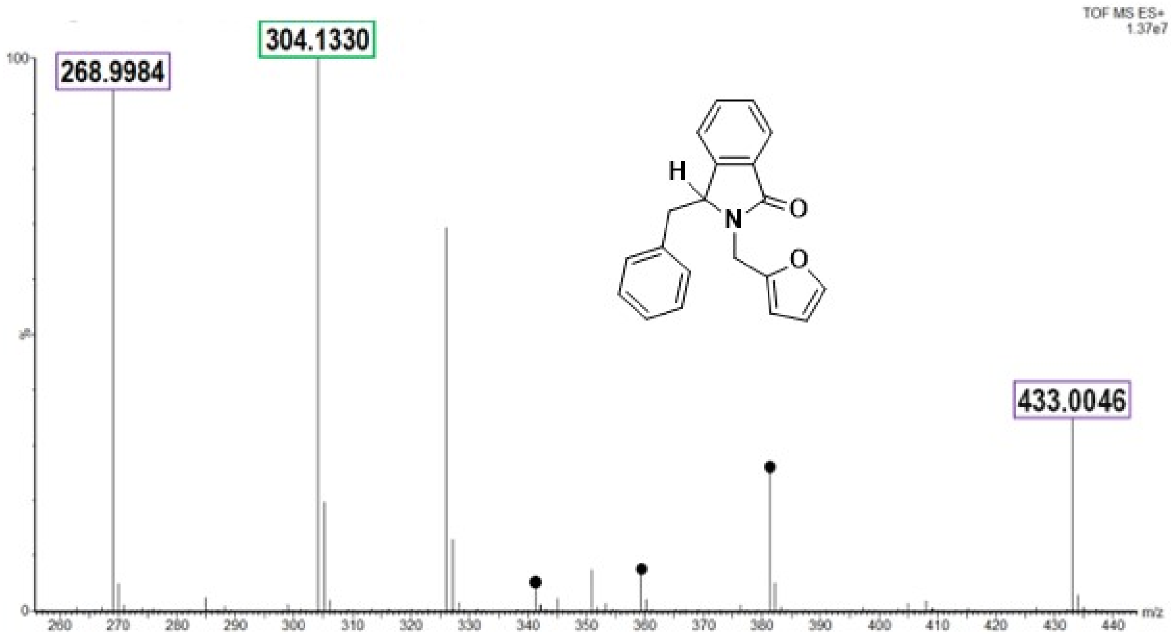
TOF MS ES+
1.37e7
304.1330
268.9984
433.0046
H
N
O
O
100
%
0
m/z

Synthesis of 3-Benzyl-2-(thiophen-2-ylmethyl)isoindolin-1-one (1l)

Following the procedure for the synthesis of 3-alkylisoindolin-1-ones **1** using (*Z*)-3-benzylideneisobenzofuran-1(3*H*)-one **2a** (111 mg, 0.5 mmol, 1 equiv.) and 2-thiophenemethylamine (0.103 mL, 1 mmol, 2 equiv.) with purification using column chromatography (*n*-hexane/ethyl acetate, 9:1), afforded the desired compound **1l.**
White solid; mp: 106-108 °C; yield: 120 mg (75%).

IR (KBr): 3032, 2973, 2928, 2860, 1682, 1420, 1080, 694, 570 cm^{-1}.

^{1}H-NMR ($CDCl_3$, 500 MHz): δ = 7.82 (d, *J* = 8.5 Hz, 1H, CH_{Ar}), 7.43-7.38 (m, 2H, CH_{Ar}), 7.29-7.23 (m, 4H, CH_{Ar}), 7.07 (d, *J* = 7.0 Hz, 2H, CH_{Ar}), 6.97-6.95 (m, 2H, CH_{Ar}), 6.89 (dd, *J* = 3.5 Hz, 1H, CH_{Ar}), 6.27 (d, *J* = 3.5 Hz, 1H, CH_{Ar}), 5.53 (d, *J* = 16.0 Hz, 1H, CH_2), 4.71 (dd, *J* = 7.5 and 5.5 Hz, 1H, CH), 4.42 (d, *J* = 15.0 Hz, 1H, CH_2), 3.41 (dd, *J* = 13.5 and 5.0 Hz, 1H, CH_2), 2.88 (dd, *J* = 13.5 and 8.0 Hz, 1H, CH_2).

^{13}C-NMR ($CDCl_3$, 125 MHz): δ = 168.3 (C=O), 145.3 (C_{Ar}), 139.6 (C_{Ar}), 136.2 (C_{Ar}), 131.9 (C_{Ar}), 131.4 (CH_{Ar}), 129.6 (2CH_{Ar}), 128.7 (2CH_{Ar}), 128.4 (CH_{Ar}), 127.2 (CH_{Ar}), 127.1 (CH_{Ar}), 126.9 (CH_{Ar}), 125.8 (CH_{Ar}), 124.0 (CH_{Ar}), 123.1 (CH_{Ar}), 59.6 (CH), 38.9 (CH_2), 38.7 (CH_2).

HR-MS (ESI): m/z $[M+H]^+$ cald for $C_{20}H_{18}NOS^+$: 320.1104, found: 320.1102.

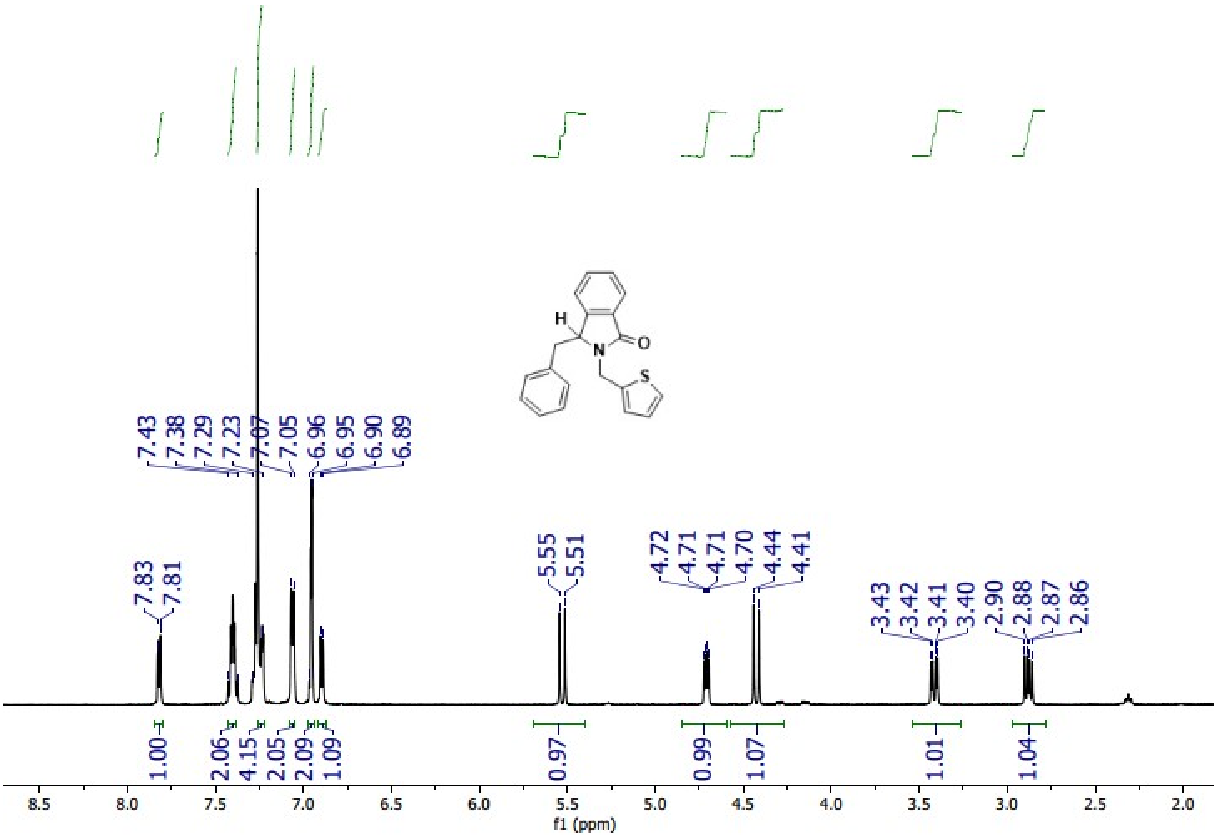

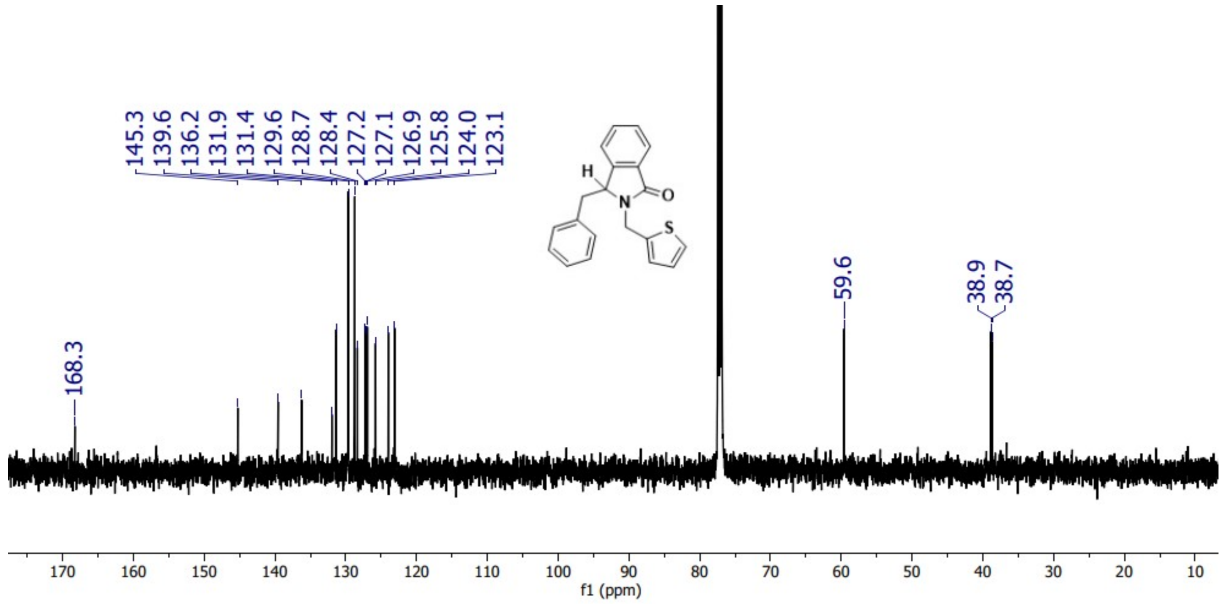
145.3
139.6
136.2
131.9
131.4
129.6
128.7
128.4
127.2
127.1
126.9
125.8
124.0
123.1
168.3
59.6
38.9
38.7
H
N
O
S
170 160 150 140 130 120 110 100 90 80 70 60 50 40 30 20 10
f1 (ppm)

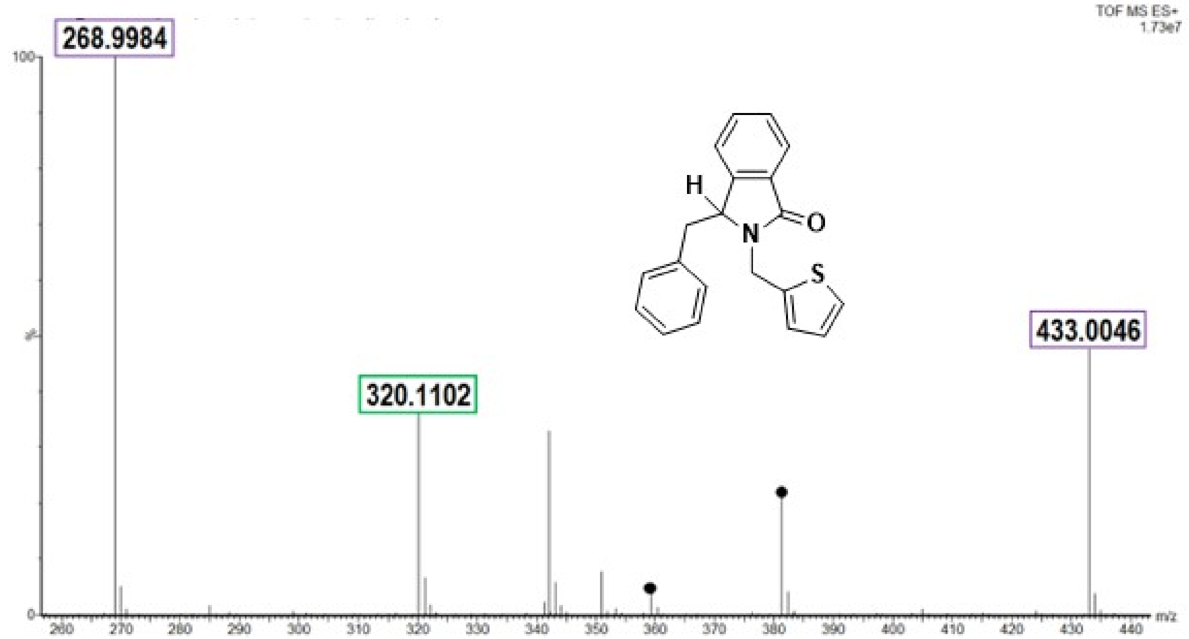
TOF MS ES+
1.73e7
268.9984
320.1102
433.0046
H
N
O
S
100
%
0
260 270 280 290 300 310 320 330 340 350 360 370 380 390 400 410 420 430 440
m/z

Synthesis of 2-Butyl-3-(4-methylbenzyl)isoindolin-1-one (1m)

Following the procedure for the synthesis of 3-alkylisoindolin-1-ones **1** using (*Z*)-3-(4-methylbenzylidene)isobenzofuran-1(3H)-one **2b** (118 mg, 0.5 mmol, 1 equiv.) and *n*-butylamine (0.099 mL, 1 mmol, 2 equiv.) with purification using column chromatography (*n*-hexane/ethyl acetate, 9:1), afforded the desired compound **1m.**
White solid; mp: 96-101 °C; yield: 121 mg (82%).

IR (KBr): 3031, 2973, 2925, 2865, 1682, 1404, 1072, 702 cm^{-1}.

^{1}H-NMR ($CDCl_3$, 500 MHz): δ = 7.77-7.76 (m, 1H, CH_{Ar}), 7.39-7.37 (m, 2H, CH_{Ar}), 7.05 (d, *J* = 8 Hz, 2H, CH_{Ar}), 6.95 (d, *J* = 8 Hz, 2H, CH_{Ar}), 6.94-6.93 (m, 1H, CH_{Ar}), 4.75 (dd, *J* = 8 and 5.0 Hz, 1H, CH), 4.10-4.04 (m, 1H, CH_2), 3.34 (dd, *J* = 14 and 4.5 Hz, 1H, CH_2), 3.20 (ddd, *J* = 14, 8.5, 5.5 Hz, 1H, CH_2), 2.77 (dd, *J* = 14 and 8 Hz, 1H, CH_2), 2.31 (s, 3H, CH_3), 1.69-1.58 (m, 2H, CH_2), 1.39-1.31 (m, 2H, CH_2), 0.94 (t, *J* = 7.0 Hz, 3H, CH_3).

^{13}C-NMR ($CDCl_3$, 125 MHz): δ = 168.4 (C=O), 145.1 (C_{Ar}), 136.7 (C_{Ar}), 133.0 (C_{Ar}), 132.6 (C_{Ar}), 130.8 (CH_{Ar}), 129.4 ($2CH_{Ar}$), 129.3 ($2CH_{Ar}$), 128.2 (CH_{Ar}), 123.6 (CH_{Ar}), 123.0 (CH_{Ar}), 60.1 (CH), 40.0 (CH_2), 38.0 (CH_2), 30.6 (CH_2), 21.2 (CH_3), 20.3 (CH_2), 13.9 (CH_3).

HR-MS (ESI): m/z $[M+H]^+$ cald for $C_{20}H_{24}NO^+$: 294.1852, found: 294.1854.

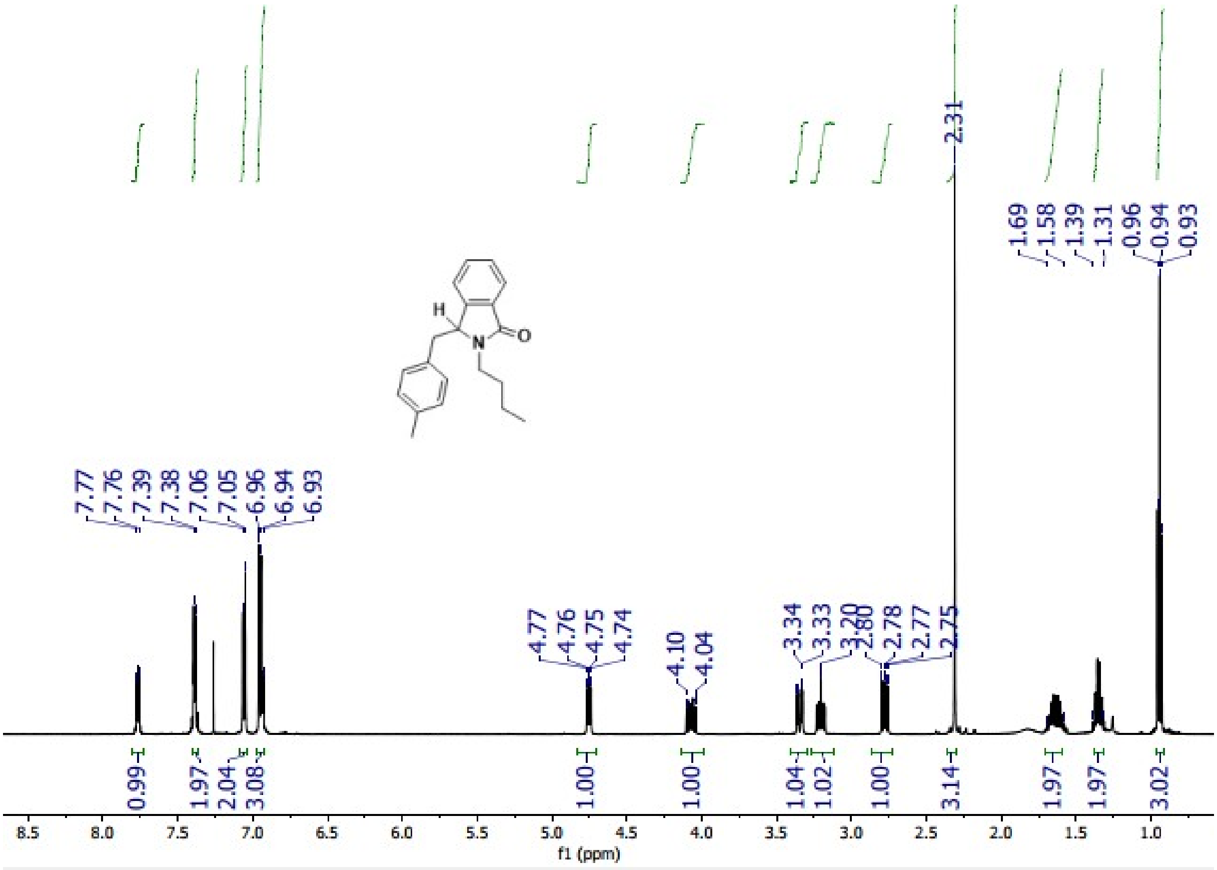

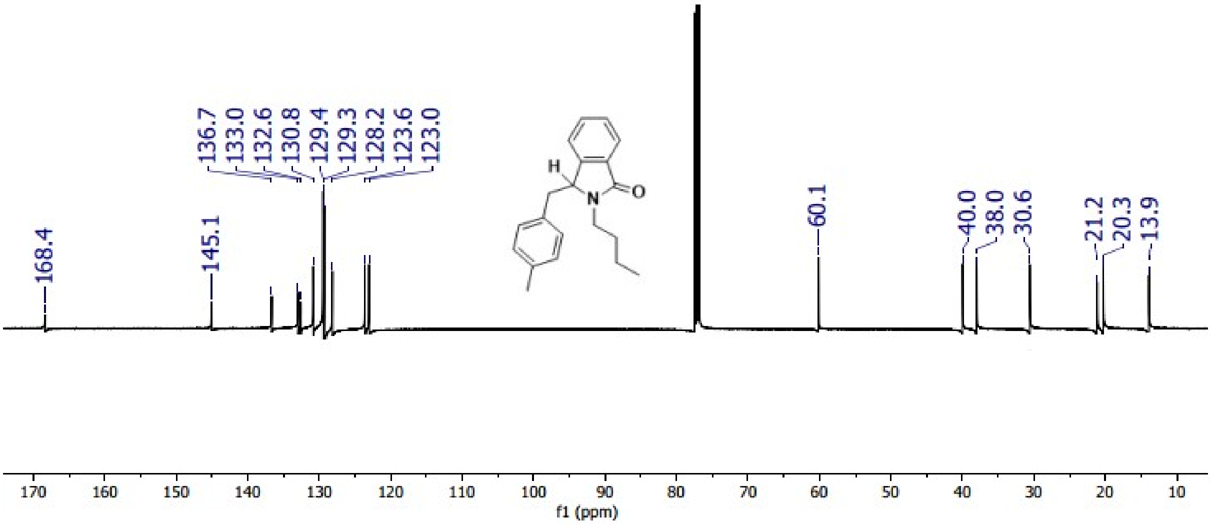
168.4
145.1
136.7
133.0
132.6
130.8
129.4
129.3
128.2
123.6
123.0
H
N
O
60.1
40.0
38.0
30.6
21.2
20.3
13.9
170 160 150 140 130 120 110 100 90 80 70 60 50 40 30 20 10
f1 (ppm)

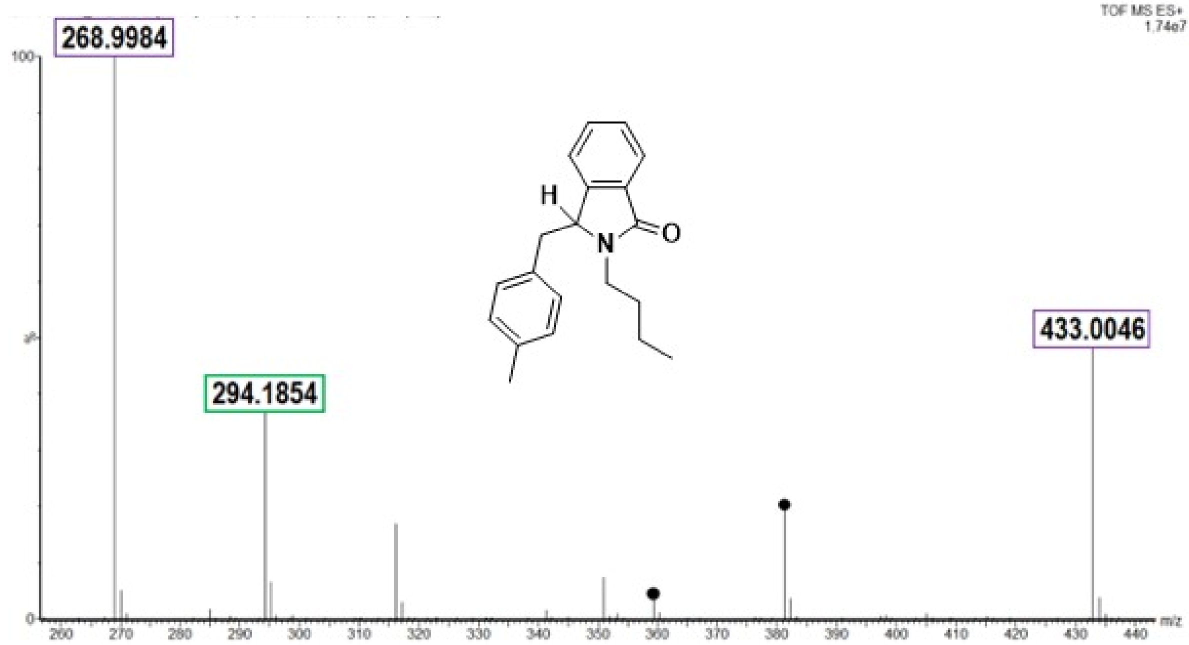
TOF MS ES+
1.74e7
268.9984
294.1854
433.0046
H
N
O
100
%
0
260 270 280 290 300 310 320 330 340 350 360 370 380 390 400 410 420 430 440
m/z

Antiplasmodium activity of 3-Benzyl-2-butylisoindolin-1-one (1a)

Strain	[M (µg/mL)]	Repetition	Infected cell	Found cell	%parasitemia	Average of %parasitemia	% inhibition	IC_{50} (µg/mL)	IC_{50} (µM)
3D7	10	1	3	1144	0.262	0.177	95.121	2.284	8.18
		2	1	1045	0.096				
		3	2	1156	0.173				
	5	1	14	1098	1.275	1.222	66.324		
		2	11	1058	1.040				
		3	14	1037	1.350				
	2.5	1	18	1061	1.697	2.138	41.059		
		2	18	1098	1.639				
		3	33	1072	3.078				
	1.25	1	17	1039	1.636	2.541	29.940		
		2	33	1076	3.067				
		3	30	1027	2.921				
	0.625	1	32	1018	3.143	2.691	25.804		
		2	29	1014	2.860				
		3	21	1014	2.071				
FCR3	10	1	2	1090	0.18	0.12	98.839	1.670	5.98
		2	0	1045	0.00				
		3	2	1048	0.19				
	5	1	16	1044	1.53	1.90	82.294		
		2	22	1054	2.09				
		3	22	1054	2.09				
	2.5	1	39	1038	3.76	4.41	58.937		
		2	44	1001	4.40				
		3	52	1023	5.08				
	1.25	1	63	1080	5.83	6.63	38.301		
		2	71	1059	6.70				
		3	76	1034	7.35				
	0.625	1	101	1105	9.14	8.42	21.637		
		2	76	1045	7.27				
		3	92	1040	8.85				

Antiplasmodium activity of 3-Benzyl-2-phenethylisoindolin-1-one (1b)

Strain	[M (μg/mL)]	Repetition	Infected cell	Found cell	%parasitemia	Average of %parasitemia	% inhibition	IC_{50} (μg/mL)	IC_{50} (μM)
3D7	10	1	70	1005	6.97	5.94	51.772	10.665	32.60
		2	66	1061	6.22				
		3	50	1081	4.63				
	5	1	71	1053	6.74	7.91	35.771		
		2	93	1000	9.30				
		3	80	1042	7.68				
	2.5	1	121	1219	9.93	9.74	20.841		
		2	94	1104	8.51				
		3	117	1084	10.79				
	1.25	1	105	1083	9.70	9.54	22.478		
		2	103	1091	9.44				
		3	103	1085	9.49				
	0.625	1	113	1091	10.36	10.99	10.753		
		2	129	1107	11.65				
		3	120	1096	10.95				
FCR3	10	1	73	1056	6.91	6.78	50.162	8.802	26.90
		2	77	1076	7.16				
		3	71	1131	6.28				
	5	1	80	1190	6.72	7.30	46.325		
		2	89	1140	7.81				
		3	79	1070	7.38				
	2.5	1	93	1132	8.22	8.33	38.770		
		2	95	1170	8.12				
		3	90	1039	8.66				
	1.25	1	109	1093	9.97	9.58	29.609		
		2	99	1020	9.71				
		3	103	1137	9.06				
	0.625	1	101	1143	8.84	10.08	25.910		
		2	111	1114	9.96				
		3	125	1092	11.45				

Antiplasmodium activity of 2,3-Dibenzylisoindolin-1-one (1c)

Strain	[M (µg/mL)]	Repetition	Infected cell	Found cell	%parasitemia	Average of %parasitemia	% inhibition	IC_{50} (µg/mL)	IC_{50} (µM)
3D7	10	1	14	1114	1.26	1.148	68.342	4.979	15.90
		2	7	1077	0.65				
		3	16	1040	1.54				
	5	1	17	1028	1.65	1.927	46.889		
		2	23	1107	2.08				
		3	22	1074	2.05				
	2.5	1	20	1020	1.96	2.203	39.275		
		2	28	1071	2.61				
		3	22	1082	2.03				
	1.25	1	38	1058	3.59	3.201	11.745		
		2	30	1126	2.66				
		3	37	1105	3.35				
	0.625	1	36	1020	3.53	3.206	11.607		
		2	27	1054	2.56				
		3	38	1077	3.53				
FCR3	10	1	48	1056	4.55	4.71	56.207	8.621	27.53
		2	50	1109	4.51				
		3	53	1047	5.06				
	5	1	62	1035	5.99	6.78	36.869		
		2	73	1095	6.67				
		3	85	1105	7.69				
	2.5	1	92	1037	8.87	7.78	27.568		
		2	95	1065	8.92				
		3	60	1080	5.56				
	1.25	1	104	1052	9.89	8.64	19.612		
		2	80	1041	7.68				
		3	90	1079	8.34				
	0.625	1	103	1109	9.29	9.26	13.819		
		2	106	1134	9.35				
		3	94	1028	9.14				

Antiplasmodium activity of 3-Benzyl-2-(4-chlorobenzyl)isoindolin-1-one (1d)

Strain	[M (μg/mL)]	Repetition	Infected cell	Found cell	%parasitemia	Average of %parasitemia	% inhibition	IC_{50} (μg/mL)	IC_{50} (μM)
3D7	10	1	17	1041	1.63	1.526	57.931	5.516	15.89
		2	19	1073	1.77				
		3	13	1107	1.17				
	5	1	21	1006	2.09	1.985	45.285		
		2	18	1050	1.71				
		3	22	1022	2.15				
	2.5	1	21	1061	1.98	2.087	42.465		
		2	18	1079	1.67				
		3	27	1033	2.61				
	1.25	1	25	1100	2.27	2.262	37.645		
		2	18	1026	1.75				
		3	28	1015	2.76				
	0.625	1	30	1109	2.71	2.636	27.324		
		2	25	1024	2.44				
		3	30	1086	2.76				
FCR3	10	1	3	1070	0.28	0.16	98.536	2.598	7.48
		2	0	1088	0.00				
		3	2	1045	0.19				
	5	1	44	1041	4.23	3.94	63.368		
		2	31	1096	2.83				
		3	48	1010	4.75				
	2.5	1	63	1022	6.16	5.97	44.456		
		2	62	1013	6.12				
		3	59	1050	5.62				
	1.25	1	101	1082	9.33	8.76	18.507		
		2	90	1113	8.09				
		3	99	1119	8.85				
	0.625	1	103	1106	9.31	8.89	17.237		
		2	106	1050	10.10				
		3	78	1073	7.27				

Antiplasmodium activity of 3-Benzyl-2-(4-fluorobenzyl)isoindolin-1-one (1e)

Strain	[M (µg/mL)]	Repetition	Infected cell	Found cell	%parasitemia	Average of %parasitemia	% inhibition	IC_{50} (µg/mL)	IC_{50} (µM)
3D7	10	1	8	1046	0.76	0.852	76.507	2.891	8.73
		2	11	1091	1.01				
		3	8	1021	0.78				
	5	1	14	1067	1.31	1.454	59.925		
		2	15	1022	1.47				
		3	17	1075	1.58				
	2.5	1	21	1077	1.95	1.746	51.858		
		2	18	1115	1.61				
		3	17	1015	1.67				
	1.25	1	22	1101	2.00	2.411	33.543		
		2	31	1092	2.84				
		3	24	1002	2.40				
	0.625	1	30	1018	2.95	3.131	13.694		
		2	34	1041	3.27				
		3	33	1038	3.18				
FCR3	10	1	16	1006	1.59	2.04	80.977	3.459	10.45
		2	15	1017	1.47				
		3	31	1011	3.07				
	5	1	67	1098	6.10	5.35	50.229		
		2	50	1011	4.95				
		3	52	1041	5.00				
	2.5	1	75	1038	7.23	6.31	41.253		
		2	62	1020	6.08				
		3	61	1083	5.63				
	1.25	1	99	1083	9.14	8.18	23.859		
		2	90	1068	8.43				
		3	80	1147	6.97				
	0.625	1	101	1066	9.47	8.57	20.213		
		2	86	1099	7.83				
		3	91	1081	8.42				

Antiplasmodium activity of 3-Benzyl-2-(4-(trifluoromethyl)benzyl)isoindolin-1-one (1f)

Strain	[M (μg/mL)]	Repetition	Infected cell	Found cell	%parasitemia	Average of %parasitemia	% inhibition	IC_{50} (μg/mL)	IC_{50} (μM)
3D7	10	1	3	1078	0.28	1.29	87.417	5.278	13.85
		2	13	1033	1.26				
		3	31	1325	2.34				
	5	1	73	1023	7.14	7.55	26.431		
		2	90	1110	8.11				
		3	86	1159	7.42				
	2.5	1	91	1069	8.51	8.21	20.003		
		2	92	1092	8.42				
		3	81	1051	7.71				
	1.25	1	98	1046	9.37	8.30	19.130		
		2	68	1022	6.65				
		3	101	1136	8.89				
	0.625	1	105	1069	9.82	9.15	10.903		
		2	100	1092	9.16				
		3	89	1051	8.47				
FCR3	10	1	20	1050	1.90	2.66	76.662	1.603	4.21
		2	27	1059	2.55				
		3	37	1047	3.53				
	5	1	49	1007	4.87	4.78	58.135		
		2	62	1070	5.79				
		3	40	1090	3.67				
	2.5	1	64	1033	6.20	5.78	49.351		
		2	54	1016	5.31				
		3	61	1047	5.83				
	1.25	1	63	1016	6.20	5.81	49.041		
		2	62	1037	5.98				
		3	56	1064	5.26				
	0.625	1	73	1046	6.98	6.65	41.744		
		2	77	1032	7.46				
		3	61	1109	5.50				

Antiplasmodium activity of 3-Benzyl-2-(4-methylbenzyl)isoindolin-1-one (1g)

Strain	[M (µg/mL)]	Repetition	Infected cell	Found cell	%parasitemia	Average of %parasitemia	% inhibition	IC_{50} (µg/mL)	IC_{50} (µM)
3D7	10	1	9	1063	0.85	0.747	79.419	2.699	8.25
		2	9	1074	0.84				
		3	6	1081	0.56				
	5	1	21	1011	2.08	1.643	54.707		
		2	10	1000	1.00				
		3	19	1026	1.85				
	2.5	1	18	1036	1.74	1.979	45.454		
		2	25	1025	2.44				
		3	18	1023	1.76				
	1.25	1	28	1022	2.74	2.230	38.538		
		2	25	2063	1.21				
		3	28	1023	2.74				
	0.625	1	28	1050	2.67	2.724	24.917		
		2	29	1071	2.71				
		3	29	1037	2.80				
FCR3	10	1	1	1084	0.09	0.18	98.314	2.214	6.77
		2	4	1120	0.36				
		3	1	1063	0.09				
	5	1	21	1018	2.06	1.64	84.737		
		2	21	1087	1.93				
		3	10	1081	0.93				
	2.5	1	63	1015	6.21	6.94	35.435		
		2	79	1109	7.12				
		3	79	1056	7.48				
	1.25	1	78	1028	7.59	7.49	30.290		
		2	83	1112	7.46				
		3	75	1011	7.42				
	0.625	1	105	1017	10.32	9.19	14.512		
		2	89	1091	8.16				
		3	96	1058	9.07				

Antiplasmodium activity of 3-Benzyl-2-(4-methoxybenzyl)isoindolin-1-one (1h)

Strain	[M (μg/mL)]	Repetition	Infected cell	Found cell	%parasitemia	Average of %parasitemia	% inhibition	IC_{50} (μg/mL)	IC_{50} (μM)
3D7	10	1	7	1209	0.58	0.821	92.008	4.213	12.28
		2	7	1003	0.70				
		3	13	1097	1.19				
	5	1	81	1031	7.86	6.606	35.670		
		2	72	1065	6.76				
		3	53	1019	5.20				
	2.5	1	74	1014	7.30	7.481	27.146		
		2	94	1129	8.33				
		3	74	1085	6.82				
	1.25	1	101	1046	9.66	8.34	18.777		
		2	93	1046	8.89				
		3	70	1081	6.48				
	0.625	1	112	1005	11.14	9.06	11.735		
		2	91	1076	8.46				
		3	80	1054	7.59				
FCR3	10	1	34	1114	3.05	3.69	65.660	4.936	14.38
		2	48	1064	4.51				
		3	38	1084	3.51				
	5	1	54	1004	5.38	5.39	49.834		
		2	59	1112	5.31				
		3	57	1039	5.49				
	2.5	1	63	1048	6.01	6.64	38.243		
		2	73	1073	6.80				
		3	79	1114	7.09				
	1.25	1	85	1060	8.02	8.94	16.787		
		2	102	1004	10.16				
		3	95	1099	8.64				
	0.625	1	104	1060	9.81	9.37	12.766		
		2	85	1048	8.11				
		3	109	1069	10.20				

Antiplasmodium activity of 3-Benzyl-2-(3-methoxybenzyl)isoindolin-1-one (1i)

Strain	[M (μg/mL)]	Repetition	Infected cell	Found cell	%parasitemia	Average of %parasitemia	% inhibition	IC_{50} (μg/mL)	IC_{50} (μM)
3D7	10	1	59	1053	5.60	5.78	53.085	9.465	27.58
		2	57	1000	5.70				
		3	63	1046	6.02				
	5	1	83	1020	8.14	8.34	32.277		
		2	83	1028	8.07				
		3	96	1091	8.80				
	2.5	1	101	1073	9.41	10.15	17.524		
		2	112	1074	10.43				
		3	110	1036	10.62				
	1.25	1	116	1000	11.60	11.47	6.831		
		2	110	1015	10.84				
		3	130	1086	11.97				
	0.625	1	124	1024	12.11	11.75	4.571		
		2	118	1037	11.38				
		3	130	1106	11.75				
FCR3	10	1	70	1081	6.48	6.55	51.879	9.686	28.23
		2	61	1041	5.86				
		3	81	1108	7.31				
	5	1	88	1085	8.11	8.20	39.722		
		2	93	1027	9.06				
		3	78	1048	7.44				
	2.5	1	112	1061	10.56	10.10	25.789		
		2	108	1053	10.26				
		3	114	1202	9.48				
	1.25	1	130	1184	10.98	10.24	24.750		
		2	109	1098	9.93				
		3	111	1131	9.81				
	0.625	1	128	1064	12.03	11.71	13.953		
		2	129	1137	11.35				
		3	120	1021	11.75				

Antiplasmodium activity of 3-Benzyl-2-(2-methoxybenzyl)isoindolin-1-one (1j)

Strain	[M (μg/mL)]	Repetition	Infected cell	Found cell	%parasitemia	Average of %parasitemia	% inhibition	IC_{50} (μg/mL)	IC_{50} (μM)
3D7	10	1	70	1041	6.72	6.01	51.190	11.941	34.80
		2	66	1083	6.09				
		3	54	1037	5.21				
	5	1	93	1019	9.13	8.95	27.296		
		2	88	1007	8.74				
		3	100	1113	8.98				
	2.5	1	103	1057	9.74	9.76	20.716		
		2	96	1012	9.49				
		3	102	1015	10.05				
	1.25	1	123	1030	11.94	10.84	11.977		
		2	105	1049	10.01				
		3	112	1061	10.56				
	0.625	1	123	1035	11.88	11.28	8.394		
		2	110	1024	10.74				
		3	120	1071	11.20				
FCR3	10	1	80	1116	7.17	6.17	54.681	10.421	30.37
		2	66	1027	6.43				
		3	50	1019	4.91				
	5	1	100	1088	9.19	8.96	34.133		
		2	96	1025	9.37				
		3	94	1128	8.33				
	2.5	1	103	1145	9.00	10.05	26.184		
		2	110	1066	10.32				
		3	116	1072	10.82				
	1.25	1	123	1142	10.77	10.74	21.070		
		2	117	1067	10.97				
		3	114	1087	10.49				
	0.625	1	130	1088	11.95	11.35	16.630		
		2	117	1039	11.26				
		3	110	1016	10.83				

Antiplasmodium activity of 3-Benzyl-2-(furan-2-ylmethyl)isoindolin-1-one (1k)

Strain	[M (µg/mL)]	Repetition	Infected cell	Found cell	%parasitemia	Average of %parasitemia	% inhibition	IC_{50} (µg/mL)	IC_{50} (µM)
3D7	10	1	4	1106	0.36	0.38	96.284	3.964	13.08
		2	3	1043	0.29				
		3	5	1009	0.50				
	5	1	70	1081	6.48	6.98	32.063		
		2	80	1037	7.71				
		3	68	1009	6.74				
	2.5	1	79	1096	7.21	7.35	28.455		
		2	78	1049	7.44				
		3	79	1068	7.40				
	1.25	1	78	1121	6.96	7.73	24.737		
		2	90	1110	8.11				
		3	92	1133	8.12				
	0.625	1	92	1038	8.86	9.19	10.528		
		2	90	1008	8.93				
		3	107	1095	9.77				
FCR3	10	1	33	1026	3.22	3.46	69.639	1.583	5.22
		2	37	1071	3.45				
		3	39	1048	3.72				
	5	1	44	1027	4.28	4.63	59.423		
		2	52	1065	4.88				
		3	51	1080	4.72				
	2.5	1	67	1097	6.11	5.37	52.977		
		2	54	1128	4.79				
		3	57	1096	5.20				
	1.25	1	60	1104	5.43	5.70	50.054		
		2	65	1061	6.13				
		3	60	1084	5.54				
	0.625	1	72	1072	6.72	6.82	40.208		
		2	82	1043	7.86				
		3	63	1070	5.89				

Antiplasmodium activity of 3-Benzyl-2-(thiophen-2-ylmethyl)isoindolin-1-one (1l)

Strain	[M (μg/mL)]	Repetition	Infected cell	Found cell	%parasitemia	Average of %parasitemia	% inhibition	IC_{50} (μg/mL)	IC_{50} (μM)
3D7	10	1	2	1017	0.20	0.13	98.721	3.034	9.50
		2	2	1014	0.20				
		3	0	1037	0.00				
	5	1	67	1063	6.30	6.28	38.816		
		2	50	1001	5.00				
		3	78	1033	7.55				
	2.5	1	72	1016	7.09	6.35	38.165		
		2	62	1030	6.02				
		3	63	1060	5.94				
	1.25	1	63	1064	5.92	7.23	29.593		
		2	75	1037	7.23				
		3	91	1066	8.54				
	0.625	1	88	1005	8.76	8.12	20.929		
		2	80	1075	7.44				
		3	83	1017	8.16				
FCR3	10	1	40	1011	3.96	3.72	67.405	1.976	6.19
		2	45	1047	4.30				
		3	31	1068	2.90				
	5	1	57	1048	5.44	4.94	56.673		
		2	51	1072	4.76				
		3	50	1079	4.63				
	2.5	1	57	1113	5.12	5.20	54.447		
		2	59	1058	5.58				
		3	51	1042	4.89				
	1.25	1	60	1065	5.63	5.48	52.010		
		2	50	1088	4.60				
		3	66	1065	6.20				
	0.625	1	81	1116	7.26	7.74	32.150		
		2	87	1123	7.75				
		3	90	1095	8.22				

Antiplasmodium activity of 2-Butyl-3-(4-methylbenzyl)isoindolin-1-one (1m)

Strain	[M (µg/mL)]	Repetition	Infected cell	Found cell	%parasitemia	Average of %parasitemia	% inhibition	IC_{50} (µg/mL)	IC_{50} (µM)
3D7	10	1	47	1022	4.60	4.95	51.750	9.955	33.96
		2	43	1066	4.03				
		3	64	1027	6.23				
	5	1	71	1020	6.96	7.43	27.653		
		2	79	1025	7.71				
		3	89	1168	7.62				
	2.5	1	106	1203	8.81	8.72	15.084		
		2	109	1295	8.42				
		3	102	1142	8.93				
	1.25	1	94	1020	9.22	9.39	8.561		
		2	94	1110	8.47				
		3	108	1030	10.49				
	0.625	1	102	1142	8.93	10.15	1.127		
		2	118	1100	10.73				
		3	116	1074	10.80				
FCR3	10	1	28	1023	2.74	3.27	71.306	2.730	9.31
		2	33	1035	3.19				
		3	39	1001	3.90				
	5	1	56	1049	5.34	5.50	51.805		
		2	68	1045	6.51				
		3	48	1032	4.65				
	2.5	1	60	1032	5.81	5.70	50.060		
		2	66	1136	5.81				
		3	57	1042	5.47				
	1.25	1	72	1063	6.77	6.70	41.249		
		2	75	1105	6.79				
		3	67	1023	6.55				
	0.625	1	74	1002	7.39	8.02	29.696		
		2	85	1032	8.24				
		3	90	1066	8.44				
